# Design, Synthesis, and Biological Evaluation of Novel Phenoxy Acetic Acid Derivatives as Selective COX-2 Inhibitors Coupled with Comprehensive Bio-Pharmacological Inquiry, Histopathological Profiling, and Toxicological Scrutiny

**DOI:** 10.3390/molecules29061309

**Published:** 2024-03-15

**Authors:** Najla A. Alshaye, Mohamed K. Elgohary, Mahmoud S. Elkotamy, Hatem A. Abdel-Aziz

**Affiliations:** 1 Department of Chemistry, College of Science, Princess Nourah bint Abdulrahman University, P.O. Box 84428, Riyadh 11671, Saudi Arabia; naalshaye@pnu.edu.sa; 2 Pharmaceutical Chemistry Department, Faculty of Pharmacy, Egyptian-Russian University, Badr City 11829, Cairo, Egypt; mahmoud-elkotamy@eru.edu.eg; 3 Applied Organic Chemistry Department, National Research Center, Dokki 12622, Cairo, Egypt

**Keywords:** anti-inflammatory, COX-1/COX-2, paw edema model, PGE-2, ulcerogenic effects

## Abstract

COX-2 plays a key role in converting arachidonic acid into prostaglandins. This makes it a significant target for treating inflammation. Selective COX-2 inhibitors have marked a new phase in inflammatory treatment, providing significant effectiveness while reducing negative side effects. Herein, we aimed at the design and synthesis of new anti-inflammatory agents **5a**–**f**, **7a**–**b**, **10a**–**f**, and **13a**–**b** with expected selective inhibition for COX-2. Compounds **5d**–**f**, **7b**, and **10c**–**f** showed significant COX-2 inhibition with IC_50_ in the range of 0.06–0.09 μM, indicating powerful pharmacological potential. In light of this, eight compounds were selected for further testing in vivo to assess their selectivity toward COX-1/COX-2 enzymes with the ability to reduce paw thickness. Compounds **5f** and **7b** showed significant anti-inflammatory effects without causing stomach ulcers, as they showed significant in vivo inhibition for paw thickness at 63.35% and 46.51%, as well as paw weight at 68.26% and 64.84%. Additionally, the tested compounds lowered TNF-α by 61.04% and 64.88%, as well as PGE-2 by 60.58% and 57.07%, respectively. Furthermore, these potent compounds were thoroughly analyzed for their pain-relieving effects, histological changes, and toxicological properties. Assessing renal and stomach function, as well as measuring liver enzymes AST and ALT, together with kidney indicators creatinine and urea, offered valuable information on their safety profiles. Molecular modeling studies explain the complex ways in which the strong interacts with the COX-2 enzyme. This comprehensive strategy emphasizes the therapeutic potential and safety profiling of these new analogues for managing inflammation.

## 1. Introduction

Inflammation is a defensive physiological reaction that occurs when dangerous stimuli, infections, or traumas disrupt tissue homeostasis. A variety of pathophysiological problems, including asthma, cerebral diseases, rheumatoid arthritis, atherosclerosis, and cancer metastasis, can result from persistent and uncontrolled inflammation [1]. 

Non-steroidal anti-inflammatory drugs (NSAIDs) are regularly administered, and they are also among the most often suggested treatments to treat pain, fever, and inflammation [2]. An estimated thirty million people take aspirin, indomethacin, ibuprofen, or other anti-inflammatory medicines every day [3]. Because of the powerful effect of NSAIDs on the COX-1 enzyme, they cause serious side effects when taken for an extended period of time. These side effects can affect the gastrointestinal, renal, liver, and cardiovascular systems [4]. NSAIDs work by blocking the action of two cyclooxygenase enzymes—constitutive COX-1 and inducible COX-2—that are essential for prostaglandin synthesis [5]. 

COX-2 differs structurally from COX-1, most noticeably by having an extra side pocket. The difference between COX-1 and COX-2 is that COX-2 has smaller amino acids (Val523, Arg513, and Val434) in comparison to COX-1’s larger amino acids (Ile523, His513, and Ile 434) [6,7]. A lot of progress has been made recently in making painkillers and anti-inflammatory drugs that target COX-2. This is because of the differences between COX-1 and COX-2 [8]. 

**Figure 1:**
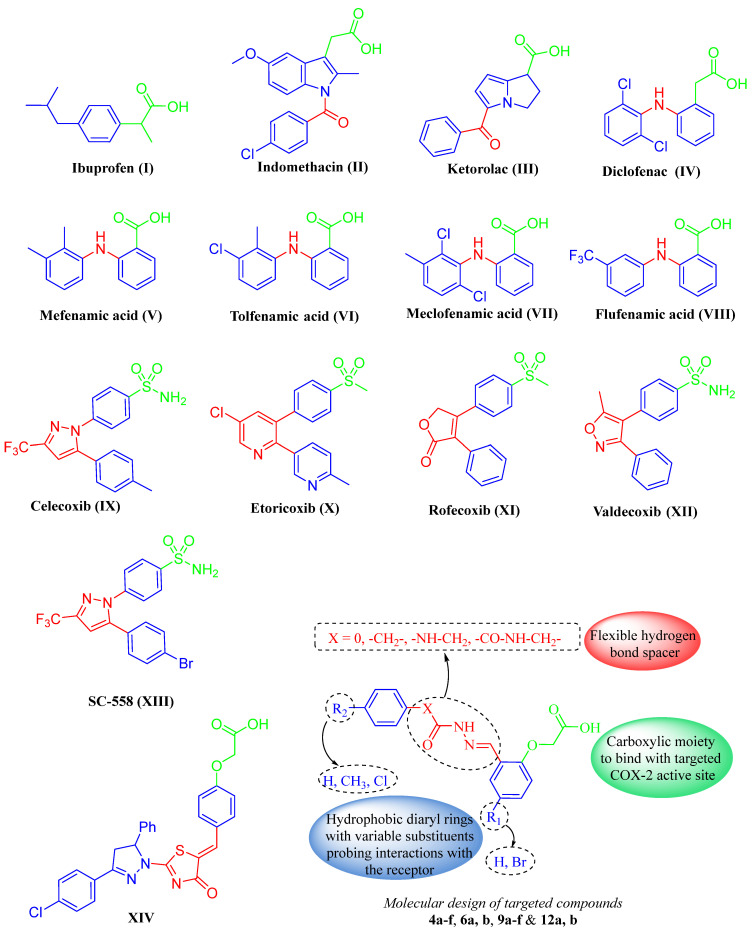
Schematic review of commercial COX-1 with marketed and reported selective COX-2 inhibitors, including the rationale for the design of new selective COX-2 inhibitors.

The selectivity of NSAIDs allows for their classification into two subclasses: non-selective NSAIDs make up the first class, such as ibuprofen (**I**) [9], indomethacin (**II**) [10], ketorolac (**III**) [11], and diclofenac (**IV**) [12]. These NSAIDs demonstrate a higher selectivity towards constitutive COX-1 than inducible COX-2, with IC_50_ = 2.9 μM, 2.9, 0.47 μM, and 0.076 μM, respectively [13] (Figure 1).

The fenamates are a class of NSAIDs that include numerous well-known pharmaceutical compounds like mefenamic acid (**V**), tolfenamic acid (**VI**), meclofenamic acid (**VII**), and flufenamic acid (**VIII**) that are derived from a fenamic acid scaffold (Figure 1). These chemicals, which differ in their carboxylic acid functionality and pKa values, show different selectivity patterns for different isoforms of cyclooxygenase. Mefenamic acid (**V**) is the most powerful COX-2 inhibitor with an IC_50_ of 5.3 μM [14], while (**VI**) inhibits COX-1 and COX-2 simultaneously because it is not selective [15]. This group of medications can reduce arthritis pain and inflammation by inhibiting the enzyme cyclooxygenase, which in turn prevents the metabolism of arachidonic acid and the production of prostaglandins [16].

Because of their specific inhibitory action on the COX-2 enzyme, this class of selective NSAIDs improves gastrointestinal tolerability and reduces the aforementioned adverse effects as compared to non-selective NSAIDs [17,18,19]. Highly selective inhibitors of COX-2 share a common structural theme when broken down into their parts: a core ring, either homocyclic or heterocyclic, with two aryl groups attached to it. One thing that makes COX-2 unique is an aryl group with a para-substitution, which is usually a methylsulfonyl (SO2Me) or aminosulfonyl (SO2NH2) group. The medication’s unique structure, which greatly increases the potential for interactions with the COX-2 enzyme, supports its exceptional selectivity and therapeutic efficacy [20]. Celecoxib (**IX**), etoricoxib (**X**), rofecoxib (**XI**), and valdecoxib (**XII**) (Figure 1) were extensively utilized for their selectivity in the COX-2 isozyme with IC_50_ = 0.05, 5.0, 0.5, and 0.005 μM, respectively [21]. Unfortunately, rofecoxib and valdecoxib were both taken off the market due to their negative consequences on cardiovascular health [22]. As a result, developing and producing more selective COX-2 inhibitors with improved safety profiles is an absolute necessity. One of the potent anti-inflammatory drugs is the 4-bromophenyl derivative SC-558 (**XIII**), which stands out for its incredible 1900-fold selectivity for COX-2 over COX-1 [23] (Figure 1).

In continuation of our previous research on developing selective COX-2 candidates [24,25], we reported phenoxyacetic acid (**XIV**), which revealed potent COX-2 activity compared to celecoxib with an IC_50_ of 0.06 μM, due to the coupling between the chlorophenyl structural motif and the *p*-phenoxy acetic acid moiety (Figure 1). 

In the current study, our objective is to develop several novel candidates, labeled **5a**–**f**, **7a**–**b**, **10a**–**f**, and **13a**–**b**, utilizing the aforementioned principles of drug design and pharmacophoric assessment of well-established pharmaceutical agents. To test their affinity toward the COX-2 active site, those substances have been carefully developed with respect to selective ligand binding interactions. This assessment involves the strategic integration of numerous chemical patterns, such as hydrogen bond spacers, carboxylic moieties, and hydrophobic di-aryl groups, one of which contains bromo substitution at position 4 (Figure 1).

## 2. Results and Discussion

### 2.1. Chemistry

**Scheme 1:**
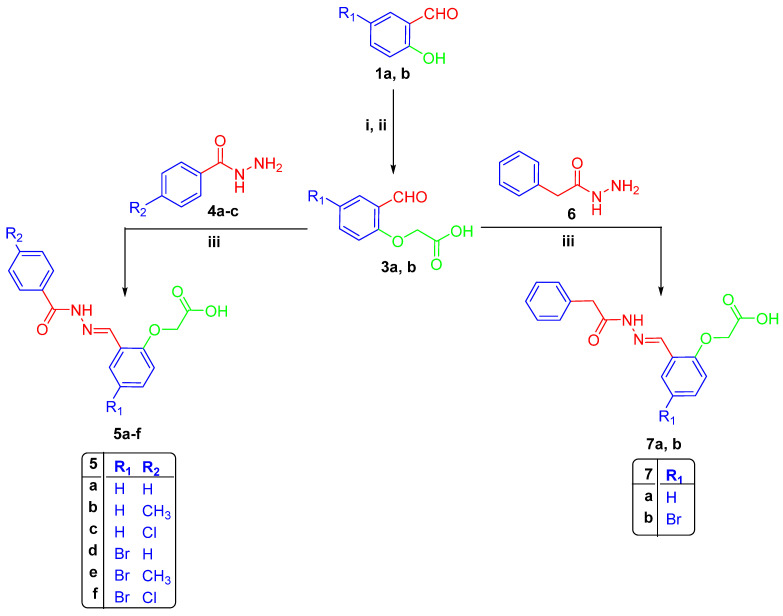
(i) DMF/K_2_CO_3_, stirring 12 h; (ii) NaOH/MeOH, reflux 12 h; (iii) EtOH/AcOH, reflux 6 h.

Aldehydes **1a**–**b** treated with ethyl bromoacetate yielded the corresponding ethyl phenoxyacetate derivatives **2a**–**b**, respectively (Scheme 1). Subsequent hydrolysis of esters **2a**–**b** resulted in the formation of phenoxyacetic acid derivatives **3a**–**b**. The latter aldehydes **3a**–**b** were then subjected to a reaction with benzohydrazide derivatives **4a**–**c** or 2-phenylacetohydrazide **6** in refluxed EtOH with a catalytic amount of acetic acid, leading to the synthesis of the targeted compounds **5a**–**f** and **7a**–**b**, respectively. 

The ^1^HNMR spectra of hydrazones **5a**–**f** exhibited three discernible peaks at approximately 4.84, 8.87, and 12.02 ppm, due to the -CH_2_-, -CH=N-, and CO_2_H protons, respectively. Additionally, methyl proton singlet signals were observed around 2.40 ppm for compounds **5b** and **5e**. As for hydrazones **7a**–**b**, a singlet signal indicative of the methylene group was observed around 4.83 ppm. Due to the presence of *cis/trans* conformers in hydrazones **7a**–**b**, their ^1^HNMR spectra revealed duplicated singlet signals for the extra methylene proton at approximately 3.54 and 4.01 ppm, -CH=N- at approximately 8.45 and 8.55 ppm, and two singlet signals for the CO_2_H proton around 11.50 and 11.85 ppm. In the ^13^CNMR spectra of hydrazones **5a**–**f** and **7a**–**b**, a signal attributed to the methylene carbon was observed around 65.45 ppm. Additionally, signals corresponding to the methyl carbon of compounds **5b** and **5e** appeared around 21.53 ppm. Furthermore, distinct signals for the carbonyl and carboxylic carbons were observed around 163.45, and 170.50 ppm for hydrazones **5a**–**f** and around 170.35, and 172.85 ppm for hydrazones **7a**–**b**.

**Scheme 2:**
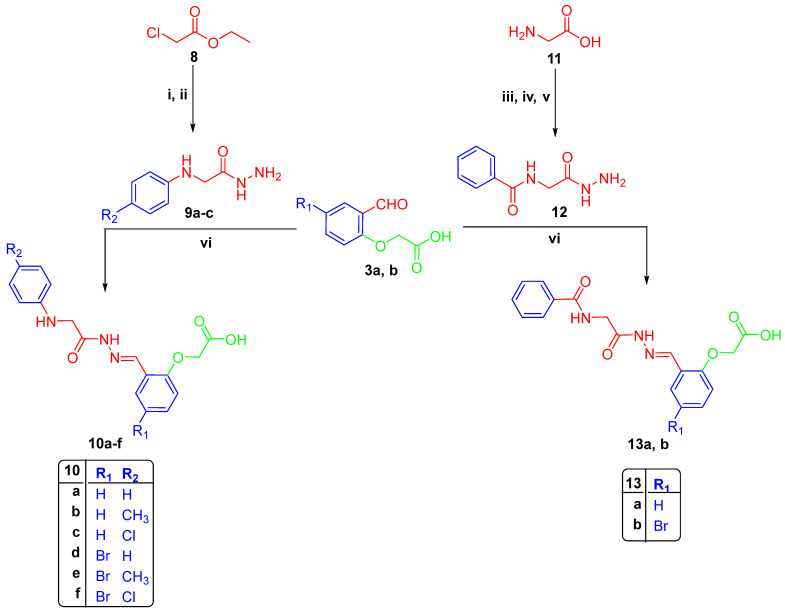
(i) Ar-NH_2_/DMF/K_2_CO_3_, stirring 12 h; (ii) NHNH_2_.H_2_O/MeOH, reflux 4 h; (iii) PhCOCl/NaOH, stirring at 0 °C 2 h; (iv) MeOH/H_2_SO_4_, reflux 6 h; (v) NHNH_2_/ MeOH, reflux 3 h; (vi) EtOH/AcOH, reflux 6 h.

The phenoxy aldehydes **3a**–**b** were subjected to treatment with 2 (phenylamino)acetohydrazide derivatives **9a**–**c** or *N*-(2-hydrazineyl-2-oxoethyl)benzamide **12** in refluxed EtOH with a catalytic amount of AcOH, resulting in the synthesis of the targeted hydrazones **10a**–**e** and **13a**–**b**, respectively (Scheme 2). The ^1^HNMR spectra of compounds **10a**–**e** and **13a**–**b** revealed the presence of a distinct signal of the methylene proton at approximately 4.85 ppm. Additionally, the presence of *cis/trans* isomers was evidenced by variant duplicated singlet signals of an extra methylene proton observed around 3.87 and 4.30 ppm, along with two singlet signals around 8.39 and 8.59 ppm for the -CH=N- proton and around 11.59, and 11.69 ppm for the CO_2_H proton. In the ^13^CNMR spectra of compounds **10a**–**e** and **13a**–**b**, duplicated singlet signals corresponding to *cis/trans* isomers of two methylene groups were observed around 44.35 and 46.50 ppm, as well as 65.39 and 65.45 ppm. Furthermore, distinct signals for the carbonyl and carboxyl carbons were noted around 170.45 and 171.90 ppm. Additionally, hydrazones **13a**–**b** exhibited three distinct signals for the two carbonyl groups and one carboxyl group, observed around 170.30, 170.40, and 17.85 ppm, respectively.

### 2.2. Biological Study

#### 2.2.1. In Vitro COX-1 and COX-2 Inhibition Assays

The Cayman^®^ colorimetric COX inhibitor screening assay kit (Item No. 560131, based in Ann Arbor, MI, USA) was employed to assess the inhibitory activity of newly synthesized compounds on ovine COX-1 and human COX-2. Compounds **5a**–**f**, **7a**–**b**, **10a**–**f**, **13a**–**b**, mefenamic acid (**V**), and celecoxib (**IX**) were tested for their ability to inhibit COX-1 and COX-2. The concentration required to inhibit enzyme activity by 50% (IC_50_) was evaluated, and COX-2 selectivity indices (SI) were calculated as COX-1 (IC_50_)/COX-2 (IC_50_). These values were then compared to those of reference drugs [26]. 

In terms of COX-1 inhibitory activity, all the examined compounds demonstrated mild to moderate inhibitory effects (IC_50_ = 4.07 ± 0.12–14.5 ± 0.2 μM). This effectiveness compares favorably with mefenamic acid (**V**) (IC_50_ = 29.9 ± 0.09 μM) and celecoxib (**IX**) (IC_50_ = 14.93 ± 0.12 μM). When it came to blocking COX-1, all of the compounds showed strong inhibitory properties except for **5c**, which had similar activity to celecoxib. Notably, in terms of inhibition against mefenamic acid, all compounds surpassed its potency. Discussing COX-1 activity results, it seems that introducing the bromo group in position 4 in compounds **5d**–**f**, **7b**, and **10d**–**f** increases COX-1 inhibition in comparison with their counterparts (IC_50_ = 9.03 ± 0.15, 7.00 ± 0.20, 8.00 ± 0.20, 5.93 ± 0.12, 7.00 ± 0.20, 4.07 ± 0.12, and 4.97 ± 0.06 μM). On the other hand, the same substitution in **13b** results in a decrease in inhibition (IC_50_ = 9.93 ± 0.12 μM) in comparison with the counterpart in **13a** (IC_50_ = 8.43 ± 0.12 μM).

Regarding COX-2 inhibitory activity, the examined compounds displayed moderate to potent inhibitory effects against the COX-2 isozyme (IC_50_ = 0.06 ± 0.01– 0.97 ± 0.06 μM), relative to mefenamic acid (**V**) (IC_50_ = 1.98 ± 0.02 μM) and celecoxib (**IX**) (IC_50_ = 0.05 ± 0.02 μM). Markedly, compounds **5d**–**f**, **7b**, and **10c**–**f** emerged as the most active, with IC_50_ values ranging from 0.06 ± 0.01 to 0.09 ± 0.01 μM. Additionally, compounds **5c**, **5d**, and **5f** demonstrated high COX-2 selectivity (SI = 111.53–133.34) when compared to celecoxib (**IX**) (SI = 298.6), as shown in Table 1 and Figure S1 (Supplementary Materials) for IC_50_ curves. 

Regarding compounds **5a**–**f**, their inhibitory efficacy exhibited diversity, with the incorporation of bromine substitution at position 4 on the phenoxy ring **5d**–**f** (IC_50_ = 0.08 ± 0.01 μM, 0.07 ± 0.01 μM, and 0.06 ± 0.01 μM, in respect) resulting in heightened activity compared to their counterparts **5a**–**c** (IC_50_ = 0.97 ± 0.06 μM, 0.37 ± 0.06 μM, and 0.13 ± 0.06 μM, in respect). It is worth emphasizing that *para*-chloro substitution on the phenyl ring **5f** displayed superior inhibitory effects in comparison to both para-methyl and unsubstituted similar, achieving an IC_50_ equal to 0.06 ± 0.01 μM. 

In the context of compounds **7a**–**b**, the introduction of bromo at position 4 on the phenoxy ring in **7b** (IC_50_ = 0.06 ± 0.01 μM) demonstrated robust inhibitory efficacy compared to the un-substituted **7a** (IC_50_ = 0.13 ± 0.06 μM). Moving on to compounds **10a**–**f**, the incorporation of bromine halogen on the phenoxy ring at position 4 in compounds **10d**–**e** (IC_50_ = 0.08 ± 0.01 μM and 0.09 ± 0.01 μM, respectively) resulted in a marked enhancement of inhibitory activity compared to their un-substituted counterparts **10a**–**b**. Conversely, both **10c** and **10f** counterparts exhibited potent inhibition against COX-2 (IC_50_ = 0.07 ± 0.01 μM and 0.06 ± 0.01 μM, respectively) (see Table 1).

**Figure 2:**
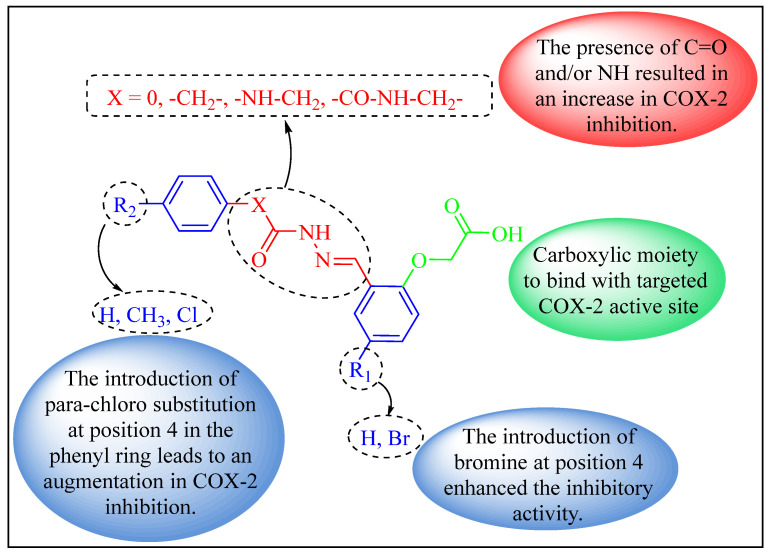
SAR analysis of most designed compounds **5a**–**f**, **7a**, **b**, **10a**–**f**, and **13a**, **b** through in vitro study of COX-2 isozyme.

Conclusively, concerning compounds **13a**–**b**, the substitution of bromo at position 4 in **13b** (IC_50_ = 0.13 ± 0.06 μM) showcased potent inhibition against its respective counterpart **13a** (IC_50_ = 0.23 ± 0.06 μM). Figure 2 concludes the SAR study of our designed compounds.

#### 2.2.2. Drug-Likeness and ADME Prediction

Using Lipinski’s rule of five and the SwissADME predictor, we evaluated the oral bioavailability and drug-likeness of the generated smaller bioactive compounds. Specifically, we looked at the most active anti-inflammatory candidates, **5d**–**f**, **7b**, and **10c**–**f** [27], as summarized in Table S1 (Supplementary Materials). All active compounds followed Lipinski’s rule of five, and a strong association was found between polar surface area, number of rotatable bonds, and medication oral bioavailability.

Additionally, the Veber rule was considered, which indicates that compounds with less than 10 rotatable bonds and a polar surface area of 140 Å^2^ should have good oral bioavailability. Using SwissADME predictors, we were able to ascertain the pharmacokinetic ADME characteristics of the top-performing medications. The ADME data for compounds **5d**–**f**, **7b**, and **10c**–**f** are shown in Figure S2 (Supplementary Materials), along with the reference medication celecoxib **IX**. These chemicals met the Lipinski and Veber criteria, suggesting they might be easily absorbed by humans. Additionally, the findings indicate that these chemicals could show good passive oral absorption, which might mean they do not cross the blood–brain barrier (BBB) or have negative effects on the central nervous system. There is hope for further optimization through in vivo research of these very active candidates, according to the ADME approach and experimental biological assessment.

#### 2.2.3. In Vivo Anti-Inflammatory Activity

**Figure 3:**
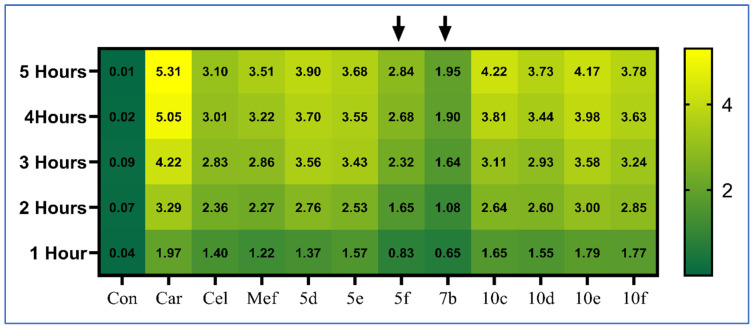
A heat map represents the paw thickness difference at hourly intervals. The darkest green color represents the lowest values, while the yellow color signifies the highest values. The intensity of colors highlights the efficacy of test compounds **5f** and **7b** (indicated with a downward arrow), with the deepest green indicating them as the most effective test compounds for inhibiting paw thickness in the carrageenan paw edema inflammation model. Con: control; Car: carrageenan; Cel: celecoxib; Mef: mefenamic acid. The data are presented in individual cells as mean values.

After our in vitro results match up with the ADME prediction evaluation for our carefully crafted drugs, we will move forward with introducing them to in vivo biological assays for more research. There was a significant increase in inflammation after carrageenan injections as compared to the healthy control group. When compared to the normal control group, rats in the carrageenan group exhibited a significant rise in paw thickness difference and percentage of paw weight gain at the 5 h mark after injection (94.04% ± 3.23). Mefenamic acid **V** (33.89% reduction) and celecoxib **IX** (41.65% reduction) in comparison to the carrageenan group had statistically significant reductions in paw thickness at the 5 h mark. Both the celecoxib (68.15% weight gain) and mefenamic acid (63.76% weight increase) groups showed a considerable improvement over the carrageenan group when compared to the others. As shown in Figure 3, the carrageenan group showed the least significant suppression of paw thickness at the 5 h mark (46.51%), whereas test compounds **7b** and **5f** showed the most significant inhibition at 63.35% and 46.51%, respectively. 

In addition, as shown in Figure S3 (Supplementary Materials), these compounds inhibited the percentage of paw weight increase the most (68.26% and 64.84%, respectively) when compared to the carrageenan group; however, they did not differ significantly from the two reference drugs. Table 2 summarizes the data on the difference in paw thickness at hourly intervals (and percentage of inhibition) and the increase in paw weight percentage for all groups.

These findings reflect those of earlier research [28,29,30]. The capacity of carrageenan to cause paw edema—a condition characterized by localized and acute inflammatory reactions—makes it an essential model for testing the efficacy of novel anti-inflammatory drugs. Researchers frequently use this well-known and reproducible model to examine acute inflammation in paw tissue [31,32]. In Table 2, we can see that test compounds **5f** and **7b** have strong anti-inflammatory properties; this is supported by the fact that they significantly reduced paw thickness and increased paw weight percentage compared to the carrageenan group, and their efficacy was equivalent to that of reference drugs.

#### 2.2.4. Assessment of Inflammatory Biomarkers

**Figure 4:**
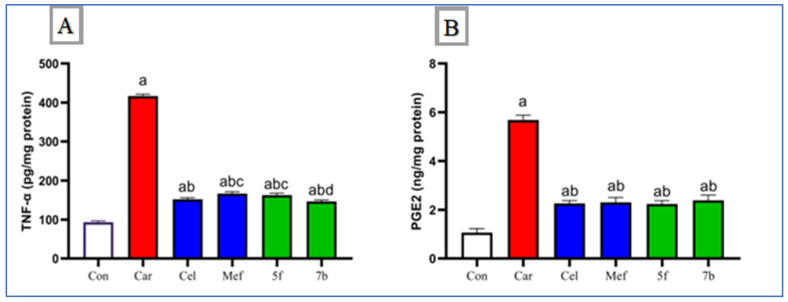
Impact of test compounds on the exudate content of TNF-α (**A**) PGE2 (**B**) in a carrageenan-induced paw edema inflammation model. The data are presented as mean ± SD and were analyzed using one-way ANOVA, followed by Tukey’s multiple comparisons test; n = 6. ^a^ Significantly different from the control group at *p* < 0.05, ^b^ Significantly different from the carrageenan group at *p* < 0.05, ^c^ Significantly different from the celecoxib group at *p* < 0.05, ^d^ Significantly different from the mefenamic acid group at *p* < 0.05. TNF-α: tumor necrosis factor-alpha; PGE2: prostaglandin E2; Con: control; Car: carrageenan; Cel: celecoxib; Mef: mefenamic acid.

Two compounds, **5f** and **7b**, were chosen for additional testing. The compounds were chosen for their distinct set of characteristics, which included lower IC_50_ values, no significant changes in the percentage of paw weight increase compared to the two reference drugs, outperforming the carrageenan group, and a comparable reduction in paw thickness difference at the 5 h mark. Celecoxib and mefenamic acid were used as reference medicines to determine the levels of tumor necrosis factor-alpha (TNF-α) and prostaglandin E2 (PGE2) in the exudates of rats that were given these substances. When compared to the normal control group, rats in the carrageenan group had substantially greater amounts of TNF-α (347.33%) and PGE2 (432.55%) in their exudate. Mefenamic acid (**V**) (60.09% and 59.37%, respectively) and celecoxib (**IX**) (63.52% and 60.16%, respectively) significantly reduced TNF-α and PGE2 exudate content as compared to the carrageenan group, as did the administration of reference medications. As shown in Figure 4, test compounds **5f** and **7b** demonstrated anti-inflammatory activity similar to reference drugs, lowering TNF-α by 61.04% and 64.88%, respectively, and PGE-2 by 60.58% and 57.07%, respectively, in line with previously noted anti-inflammatory effects.

These results corroborate the beneficial anti-inflammatory action of the substances tested, which is in line with earlier research. Their anti-inflammatory effectiveness is further supported by the decreases in TNF-α and PGE-2 exudate content that have been seen [33,34]. There are two distinct stages in the development of carrageenan-induced paw edema. The first stage happens within an hour of induction, and the second phase occurs between two and three hours after induction. Thrombin, serotonin, histamine, bradykinin, and vascular endothelial growth factor are responsible for the early thickening of paw edema. Later on, local neutrophil infiltration, prostaglandin production, nitric oxide (NO), and oxygen-derived free radicals are associated with the persistent increase in paw edema thickness [35,36]. The inflammatory response cannot be started or controlled without the pro-inflammatory cytokine TNF-α. TNF-α manages cellular processes, recruits and activates immune cells, induces other cytokines that promote inflammation and plays a crucial role in the inflammatory process. Important insights on paw tissue inflammatory activity and potential protective benefits may be gained by measuring TNF-α and PGE2 levels [37,38]. 

#### 2.2.5. Analgesic Activity

The hot plate latency test was used to evaluate the analgesic efficacy of test compounds **5f** and **7b**. Mefenamic acid (**V**) and celecoxib (**IX**) were used as reference medications. Mefenamic acid’s highest activity (58.38% rise in the latency period) was at the 60 min mark, whereas celecoxib’s greatest analgesic activity was at the 120 min mark, with a latency period increase of 46.70%. The sequence in which the analgesic effects of the two test substances were observed is as follows: **5f** > **7b**, with a steep climb until the 120 min mark (49.50% and 44.90% increase in latency time, respectively), starting with a quick commencement (39.43% and 37.67% increase in latency period, respectively) at the 30 min mark. Table 3 shows the analgesic effects at 30, 60, 90, and 120 min.

The analgesic efficacy of all reference medications and test compounds that were examined is summarized in Table 3. The hot plate approach, which uses thermally produced pain, may accurately assess centrally mediated nociception. The results of this study provide more evidence that the chemicals being studied have the potential to lengthen the latency time for pain stimuli, indicating that they may have an effective central analgesic impact. When combined with previous findings, the possibility that these two test substances would exhibit strong anti-inflammatory and analgesic effects upon observation is highlighted [39].

#### 2.2.6. Histopathological Examination

**Figure 5:**
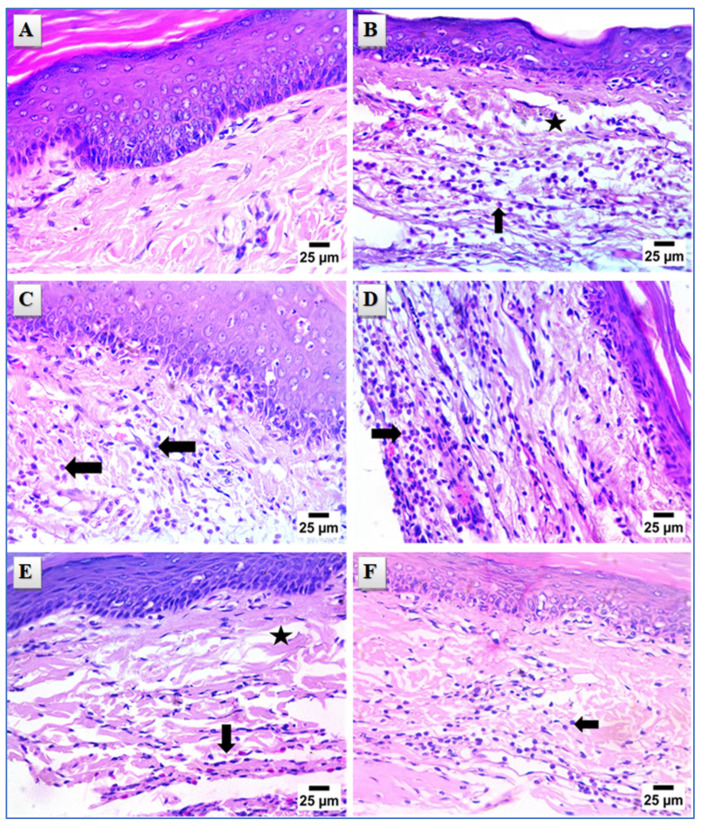
Histopathological photomicrographs illustrate the evident characteristics of an inflammatory model of paw edema across multiple experimental groups. (**A**) Normal control group demonstrates the normal histological structure of the epidermis and dermis; (**B**) carrageenan group demonstrates dermal edema (star) with infiltration by inflammatory cells mainly lymphocytes and eosinophils (arrow); (**C**) celecoxib group demonstrates dermal infiltration by high number of inflammatory cells mainly neutrophils and lymphocytes (arrows); (**D**) mefenamic acid group demonstrates infiltration of dermis by high number of inflammatory cells mainly lymphocytes and eosinophils (arrow); (**E**) **5f** group demonstrates dermal edema (star) with infiltration by a few numbers of inflammatory cells mainly eosinophils and lymphocytes (arrow); (**F**) **7b** group demonstrates dermal infiltration by a moderate number of mononuclear inflammatory cells (arrow) (Hematoxylin and Eosin staining).

As shown in Figure 5, the histopathological investigation showed clear variations between the experimental groups. The control specimens showed typical histological characteristics in the epidermis and dermis, suggesting no signs of inflammation. On the other hand, specimens treated with carrageenan showed evident edema in the skin, along with the presence of inflammatory cells, namely lymphocytes and eosinophils, suggesting an inflammatory reaction [31]. 

The specimens treated with celecoxib had significant dermal infiltration by a large quantity of inflammatory cells, mostly neutrophils, and lymphocytes, indicating an inflammatory response related to the drug. Specimens treated with mefenamic acid showed significant cutaneous infiltration by inflammatory cells, particularly lymphocytes, and eosinophils, reflecting the inflammatory response seen in the carrageenan group. Specimens treated with compounds **5f** and **7b** showed less severe inflammatory alterations. Compound **5f** exhibited dermal edema with limited infiltration of inflammatory cells, mostly eosinophils, and lymphocytes, whereas compound **7b** showed moderate infiltration of mononuclear inflammatory cells in the dermis. The results indicate that compounds **5f** and **7b** may have anti-inflammatory capabilities since they showed reduced inflammatory reactions compared to the reference drugs and carrageenan group [40].

**Figure 6:**
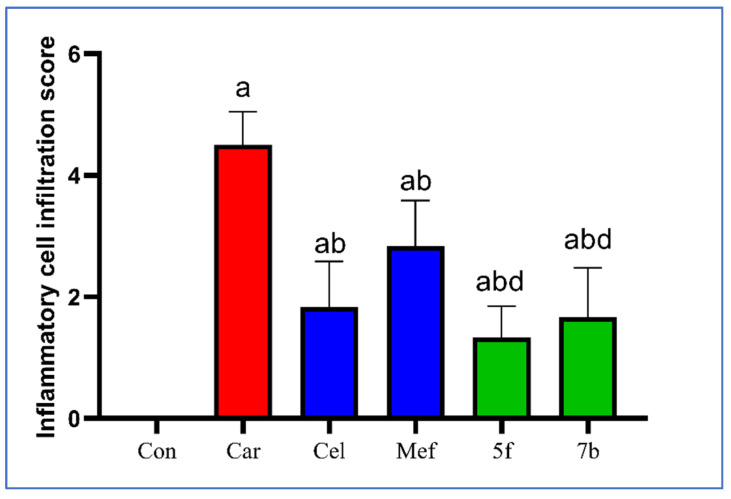
Inflammatory cell infiltration score in a carrageenan-induced paw edema inflammation model. The data are presented as mean ± SD and were analyzed using one-way ANOVA, followed by Tukey’s multiple comparisons test; n = 6. ^a^ Significantly different from the control group at *p* < 0.05, ^b^ Significantly different from the carrageenan group at *p* < 0.05, ^d^ Significantly different from the mefenamic acid group at *p* < 0.05. Con: control; Car: carrageenan; Cel: celecoxib; Mef: mefenamic acid.

Animals treated with carrageenan showed a significant increase in inflammatory cell infiltration compared to the normal control group. Administering reference drugs such as celecoxib or mefenamic acid led to a significant reduction in infiltration scores (59.26% and 37.04%, respectively) compared to the carrageenan group. Test compounds **5f** and **7b** showed a significant decrease in scoring: **5f** by 70.37% and **7b** by 62.96% compared to the carrageenan group. The same groups, **5f** and **7b**, did not exhibit any significant differences compared to the celecoxib group but showed improved effectiveness compared to the mefenamic acid group (Figure 6).

#### 2.2.7. Toxicity Assessment

##### Assessment of Liver and Kidney Function

Celecoxib (**IX**) and mefenamic acid (**V**), two reference medications, and the two test compounds **5f** and **7b** were all tested for changes in renal and liver function. The reference medications were compared to the test compounds. Table 4 displays the results for serum ALT, AST, creatinine, urea, and the AST/ALT ratio. Neither the normal control group nor the carrageenan group exhibited statistically significant changes in these measures compared to the other groups or reference medicines. These results suggest that the two most active chemicals in the test, **5f** and **7b**, may have a relatively low risk of side effects on the liver and kidney

##### Evaluation of Ulcerogenic Effects

**Figure 7:**
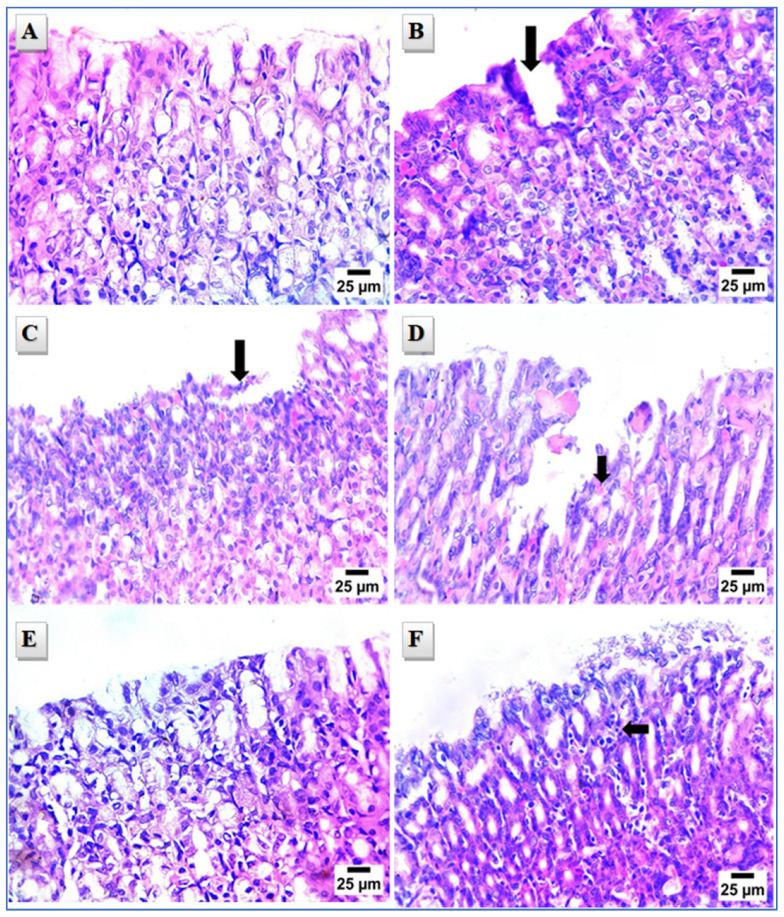
Histopathological photomicrographs of the gastric mucosa of the following: (**A**) Control group demonstrates normal histological structure of gastric mucosa; (**B**) carrageenan group demonstrates small ulcer formation at superficial part of gastric mucosa (arrow); (**C**) celecoxib group demonstrates ulcer formation with eroded gastric mucosa (arrow); (**D**) mefenamic acid group demonstrates ulcer formation with desquamation of gastric mucosal epithelium (arrow); (**E**) **5f** group demonstrates normal histological structure of gastric mucosa; (**F**) **7b** group demonstrates infiltration of gastric mucosa by a few numbers of mononuclear inflammatory cells (arrow) (Hematoxylin and Eosin staining).

Test compounds **5f** and **7b**, along with reference drugs celecoxib and mefenamic acid, were subjected to macroscopic and microscopic assessment for ulcerogenic changes, following previously described methodologies [41,42]. Treatment with mefenamic acid led to a significant increase in both ulcer number (233.33%) and severity score (366.67%) compared to the normal control group. Otherwise, all groups showed non-significant changes in either number or severity score among their means, except for the comparison between the mefenamic acid group and compound **5f** group, as presented in Table 5. In the histopathological examination (Figure 7), the control group showed a normal histological structure of the gastric mucosa; however, the carrageenan group showed minor ulcer development at the surface of the gastric mucosa. Notably, the celecoxib group developed ulcers with degraded gastric mucosa, while mefenamic acid therapy caused ulcer development and desquamation of the stomach mucosal epithelium. In contrast, the **5f** and **7b** groups showed normal histological structure of the stomach mucosa and infiltration by a few mononuclear inflammatory cells, respectively. These results demonstrate that the two compounds under investigation had a positive safety profile regarding the development of gastrointestinal ulcers, a typical side effect of many anti-inflammatory analgesics [43,44].

### 2.3. Molecular Docking Study

The docking study was conducted to gain deeper insights into the COX-2 activity and the specific binding sites of the most promising anti-inflammatory drugs, **5f** and **7b**, which were identified through in vivo biological assessment, toxicity study, and ulcer activity testing using the Autodock Vina software (https://vina.scripps.edu/). Out of the ten possible docking poses that were returned, the one that was most similar to the co-crystallized ligand’s binding mode was chosen. 

#### 2.3.1. Docking Validation Method

The docking approach was validated by redocking the co-crystallized celecoxib **IX** into the COX-2 active site (PDB: 1CX2). The redocked celecoxib showed a very similar placement to the co-crystallized celecoxib, with a docking score of −11.0 kcal/mol. This result highlights the dependability of the docking approach used in this work for properly forecasting the binding orientation of the chemical being studied (Figure S4, Supplementary Materials).

#### 2.3.2. Molecular Docking of Most Potent Candidates **5f** and **7b** within COX-2 Active Sites

**Figure 8:**
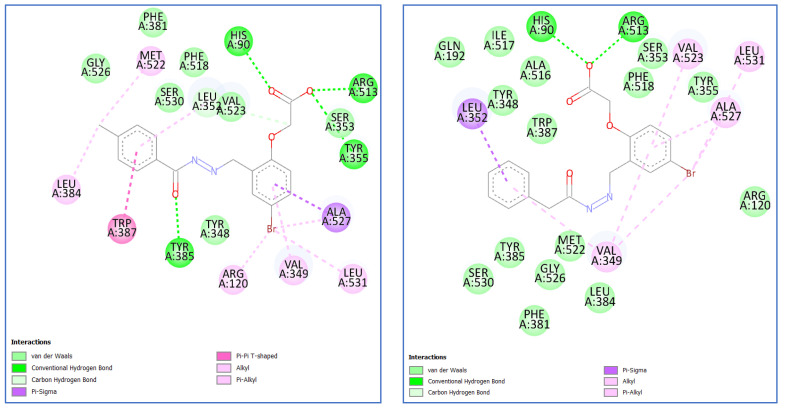
Two-dimensional schematic diagram of potent compounds **5f** (**left**) and **7b** (**right**) on the active site of COX-2 enzyme.

Following foundational design principles and methodological rationale, as well as the investigation about the interaction pockets of the COX-1 and COX-2 active sites [45,46]. The docking conformation of compound **5f** exhibited precise overlay within the active site, wherein the carboxylate moiety established hydrogen bond interactions with Arg513 and His90, while the carbonyl group within the carboxylate moiety engaged in hydrogen bonding with Tyr355. Furthermore, the carbonyl group within the hydrazide spacer formed a hydrogen bond interaction with Tyr385. As for compound **7b**, it likewise manifested a congruent alignment within the active site, wherein the carboxylate moiety established hydrogen bond interactions with Arg513 and His90, whereas the phenoxy ring interacted with Leu352 via *pi*-sigma interaction, as delineated in Table 6 and illustrated in Figure 8.

## 3. Materials and Methods

### 3.1. Chemistry

Benzohydrazides **4a**–**c** [47], 2-phenylacetohydrazide **6** [48], 2-(phenylamino)acetohydrazide **9a**–**c** [49], and *N*-(2-hydrazineyl-2-oxoethyl)benzamide **12** [50] were synthesized following the procedures in the literature. The specifications of the tools used in the chemical section are mentioned in Supplementary Data, page 52.

#### 3.1.1. Synthesis of 2-(4-formylphenoxy)acetic Acids **3a**–**b**

A solution comprising (20 mmol) of aldehydes **1a**–**b**, along with (3.34 g, 20 mmol) of ethyl bromoacetate in 30 mL of DMF, was treated with (5.52 g, 40 mmol) of K_2_CO_3_. The resulting mixture was then stirred for 12 h, yielding the corresponding ethyl 2-(2-formylphenoxy)acetates **2a**–**b**. Subsequently, the esters were subjected to hydrolysis by a mixture of aqueous NaOH and MeOH at 20 °C for 12 h, resulting in the formation of 2-(2-formylphenoxy)acetic acids **3a**–**b**, respectively, m.p. of **3a** = 132 °C (reported m.p. = 189–191 °C) and m.p. of **3b** = 177 °C (reported m.p. = 165 °C) [51].

##### Synthesis of Hydrazones **5a**–**f**, **7a**–**b**, **10a**–**f**, and **13a**–**b**

A mixture containing (2 mmol) of 2-(2-formylphenoxy)acetic acid derivatives **3a**–**b**, along with (2 mmol) of hydrazides **4a**–**c**, **6**, **9a**–**c**, or **12**, was refluxed in (30 mL) of absolute EtOH and (0.3 mL) of acetic acid for 6 h. Upon cooling, the resulting precipitate was filtered, dried, and subsequently crystallized from a mixture of EtOH/DMF, yielding the desired final hydrazones **5a**–**f**, **7a**–**b**, **10a**–**f**, and **13a**–**b**, respectively. The physical properties and spectral analysis of compounds **5a**, **5c**, **5d**, and **7a** are identical to those reported previously [51].

2-(2-((2-Benzoylhydrazineylidene)methyl)phenoxy)acetic acid (**5a**)

White powder, 74% yield; mp 209–211 °C; IR (KBr) *ν*_max_/cm^−1^ 1720 (>C=O), 2121–3410 (-CO_2_H), 3278 (>NH). ^1^HNMR (DMSO-*d*_6_) *δ* 4.84 (s, 2H, -CH_2_-), 7.04 (q, *J* = 8.8 Hz, 2H, ArHs), 7.41 (t, *J* = 8.8 Hz, 1H, ArH), 7.54 (t, *J* = 7.2 Hz, 2H, ArHs), 7.61 (t, *J* = 7.2 Hz, 1H, ArH), 7.94 (m, 3H, ArHs) 8.89 (s, 1H, -CH=N-), 11.98 (1s, D_2_O exchangeable, 1H, -NH-). ^13^CNMR (DMSO-*d*_6_) *δ* 65.35 (-CH_2_-), 113.14, 121.75 122.01, 123.22, 126.22, 128.14, 128.91, 131.81, 132.19, 133.87, 143.70, 156.67, 156.80, 163.51 (>C=O), 170.51 (-CO_2_H). Anal. Calcd. For: C_16_H_14_N_2_O_4_ (298.30): C, 64.42; H, 4.73; N, 9.39; found: C, 64.57, H, 4.85, N, 9.49.

2-(2-((2-(4-Methylbenzoyl)hydrazineylidene)methyl)phenoxy)acetic acid (**5b**)

White powder, 77% yield; mp 222–223 °C; IR (KBr) *ν*_max_/cm^−1^ 1721 (>C=O), 2131–3367 (-CO_2_H), 3155 (>NH). ^1^HNMR (DMSO-*d*_6_) *δ* 2.40 (s, 3H, -CH_3_), 4.84 (s, 2H, -CH_2_-), 7.05 (q, *J* = 8.8 Hz, 2H, ArHs), 7.35 (d, *J* = 7.6 Hz, 2H, ArHs), 7.42 (t, *J* = 8.8 Hz, 1H, ArH), 7.87 (d, *J* = 8.0 Hz, 2H, ArHs), 7.92 (d, *J* = 7.6 Hz, 1H, ArH), 8.88 (s, 1H, -CH=N-), 11.91 (1s, D_2_O exchangeable, 1H, -NH-). ^13^CNMR (DMSO-*d*_6_) *δ* 21.52 (-CH_3_), 65.31 (-CH_2_-), 113.13, 121.75, 123.29, 126.20, 128.16 (2C), 129.44 (2C), 130.99, 131.73, 142.22, 143.39, 156.75, 163.33 (>C=O), 170.51 (-CO_2_H). Anal. Calcd. For: C_17_H_16_N_2_O_4_ (312.33): C, 65.38; H, 5.16; N, 8.97; found: C, 65.57, H, 5.23, N, 9.08.

2-(2-((2-(4-Chlorobenzoyl)hydrazineylidene)methyl)phenoxy)acetic acid (**5c**)

White powder, 71% yield; mp 241–243 °C; IR (KBr) *ν*_max_/cm^−1^ 1720 (>C=O), 2044–3367 (-CO_2_H), 3151 (>NH). ^1^HNMR (DMSO-*d*_6_) *δ* 4.84 (s, 2H, -CH_2_-), 7.06 (q, *J* = 8.4 Hz, 2H, ArHs), 7.41 (t, *J* = 8.8 Hz, 1H, ArH), 7.62 (d, *J* = 8.4 Hz, 2H, ArHs), 7.92 (d, *J* = 7.6 Hz, 1H, ArH), 7.99 (d, *J* = 8.4 Hz, 2H, ArHs), 8.88 (s, 1H, -CH=N-), 12.04 (1s, D_2_O exchangeable, 1H, -NH-); ^13^CNMR (DMSO-*d*_6_) *δ* 65.32 (-CH_2_-), 113.17, 121.77, 123.11, 126.23, 129.01 (2C), 130.09 (2C), 131.92, 132.59, 137.01, 144.00, 156.82, 162.43 (>C=O), 170.48 (-CO_2_H). Anal. Calcd. For: C_16_H_13_ClN_2_O_4_ (332.74): C, 57.76; H, 3.94; N, 8.42; found: C, 57.82; H, 3.99; N, 8.53.

2-(2-((2-Benzoylhydrazineylidene)methyl)-4-bromophenoxy)acetic acid (**5d**)

White powder, 73% yield; mp 244–245 °C; IR (KBr) *ν*_max_/cm^−1^ 1719 (>C=O), 2106–3425 (-CO_2_H), 3201 (>NH). ^1^HNMR (DMSO-*d*_6_) *δ* 4.86 (s, 2H, -CH_2_-), 7.05 (d, *J* = 8.4 Hz, 1H, ArH), 7.53–7.64 (m, 4H, ArHs), 7.95–8.00 (m, 3H, ArHs), 8.81 (s, 1H, -CH=N-), 12.10 (1s, D_2_O exchangeable, 1H, -NH-); ^13^CNMR (DMSO-*d*_6_) *δ* 65.60 (-CH_2_-), 113.53, 115.84, 125.48, 128.12 (2C), 128.17 (2C), 128.96, 132.35, 133.64, 133.87, 141.96, 155.95, 163.63 (>C=O), 170.23 (-CO_2_H). Anal. Calcd. For: C_16_H_13_BrN_2_O_4_ (377.19): C, 50.95; H, 3.47; N, 7.43; found: C, 51.05; H, 3.53; N, 7.46.

2-(4-Bromo-2-((2-(4-methylbenzoyl)hydrazineylidene)methyl)phenoxy)acetic acid (**5e**)

White powder, 74% yield; mp 277–278 °C; IR (KBr) *ν*_max_/cm^−1^ 1716 (>C=O), 2134–3406 (-CO_2_H), 3194 (>NH). ^1^HNMR (DMSO-d6) δ 2.40 (s, 3H, -CH_3_), 4.86 (s, 2H, -CH_2_-), 7.05 (d, *J* = 8.8 Hz, 1H, ArH), 7.35 (d, *J* = 8.0 Hz, 2H, ArHs), 7.56 (d, *J* = 8.8 Hz, 1H, ArH), 7.87 (d, *J* = 7.6 Hz, 2H, ArHs), 7.99 (s, 1H, ArH), 8.80 (s, 1H, -CH=N-), 12.02 (1s, D2O exchangeable, 1H, -NH-); ^13^CNMR (DMSO-d6) δ 21.53 (-CH_3_), 65.58 (-CH_2_-), 113.52, 115.82, 125.55, 128.09, 128.19 (2C), 129.48 (2C), 130.75, 133.79, 141.64, 142.41, 155.90, 163.44 (>C=O), 170.23 (-CO_2_H). Anal. Calcd. For: C_17_H_15_BrN_2_O_4_ (391.22): C, 52.19; H, 3.86; N, 7.16; found: C, 52.29; H, 3.94; N, 7.21.

2-(4-Bromo-2-((2-(4-chlorobenzoyl)hydrazineylidene)methyl)phenoxy)acetic acid (**5f**)

White powder, 73% yield; mp 261–262 °C; IR (KBr) *ν*_max_/cm^−1^ 1716 (>C=O), 2101–3410 (-CO_2_H), 3278 (>NH). IR (KBr) *ν*_max_/cm^−1^ 1716 (>C=O), 2314–3410 (-CO_2_H), 3140 (>NH). ^1^HNMR (DMSO-d6) δ 4.86 (s, 2H, -CH_2_-), 7.06 (d, *J* = 8.8 Hz, 1H, ArH), 7.56–6.64 (m, 3H, ArHs), 7.56 (d, *J* = 8.8 Hz, 1H, ArH), 7.98 (d, *J* = 7.6 Hz, 3H, ArHs), 8.79 (s, 1H, -CH=N-), 12.15 (1s, D2O exchangeable, 1H, -NH-); ^13^CNMR (DMSO-d6) δ 65.60 (-CH_2_-), 113.54, 115.86, 125.35, 128.12, 129.07 (2C), 130.11 (2C), 132.35, 133.99, 137.18, 142.28, 155.98, 162.57 (>C=O), 170.22 (-CO_2_H). Anal. Calcd. For: C_16_H_12_BrClN_2_O_4_ (411.64): C, 46.69; H, 2.94; N, 6.81; found: C, 46.74; H, 2.99; N, 6.89.

2-(2-((2-(2-Phenylacetyl)hydrazineylidene)methyl)phenoxy)acetic acid (**7a**)

White powder, 69% yield; mp 214–215 °C; IR (KBr) *ν*_max_/cm^−1^ 1728 (>C=O), 2152–3400 (-CO_2_H), 3159 (>NH). ^1^HNMR (DMSO-*d*_6_) *δ* 3.54 and 4.00 (2s, 2H, -CH_2_-), 4.82 (s, 2H, -CH_2_-), 6:.99–7.07 (m, 2H, ArHs), 7.23–7.40 (m, 6H, ArHs), 7.82 and 7.89 (2d, *J* = 8.8/8.8 Hz, 1H, ArH), 8.42 and 8.63 (2s, 1H, CH=N-), 11.44 and 11.73 (2s, D_2_O exchangeable, 1H, -NH-), ^13^CNMR (DMSO-*d*_6_) *δ* 41.73 (-CH_2_-), 65.36 (-CH_2_-), 113.09, 113.19, 121.76, 121.78, 123.04, 123.18, 125.95, 126.08, 126.84, 127.07, 128.71, 128.81, 129.54, 129.86, 131.44, 131.75, 136.22, 136.30, 139.02, 142.35, 156.60, 166.93 170.47, 170.53 (>C=O), 172.74 (-CO_2_H). Anal. Calcd. For: C_17_H_16_N_2_O_4_ (312.33): C, 65.38; H, 5.16; N, 8.97; found: C, 65.44, H, 5.23, N, 9.08.

2-(4-Bromo-2-((2-(2-phenylacetyl)hydrazineylidene)methyl)phenoxy)acetic acid (**7b**)

White powder, 68% yield; mp 277 °C; IR (KBr) *ν*_max_/cm^−1^ 1720 (>C=O), 2156–3300 (-CO_2_H), 3163 (>NH). ^1^HNMR (DMSO-*d*_6_) *δ* 3.55 and 4.01 (2s, 2H, -CH_2_-), 4.83 (s, 2H, -CH_2_-), 6.99–7.05 (m, 1H, ArHs), 7.22–7.34 (m, 5H, ArHs), 7.54 (t, *J* = Hz, 1H, ArH), 7.88 and 7.93 (2s, 1H, ArH), 8.32 and 8.54 (2s, 1H, CH=N-), 11.53 and 11.85 (2s, D_2_O exchangeable, 1H, -NH-), ^13^CNMR (DMSO-*d*_6_) *δ* 41.71 (-CH_2_-), 65.64 (-CH_2_-), 113.51, 113.57, 115.71, 115.89, 125.31, 125.44, 126.85, 127.11, 127.96, 127.99, 128.70, 128.84, 129.54, 129.84, 133.54, 136.06, 136.26, 137.37, 140.6, 155.77, 155.84, 167.13, 170.21, 170.26 (>C=O), 172.91 (-CO_2_H). Anal. Calcd. For: C_17_H_15_BrN_2_O_4_ (391.22): C, 52.19; H, 3.86; N, 7.16; found: C, 52.29; H, 3.89; N, 7.22.

2-(2-((2-(Phenylglycyl)hydrazineylidene)methyl)phenoxy)acetic acid (**10a**)

White powder, 75% yield; mp 221–222 °C; IR (KBr) *ν*_max_/cm^−1^ 1728 (>C=O), 2214–3500 (-CO_2_H), 3170 (>NH), 3425 (>NH). ^1^HNMR (DMSO-*d*_6_) *δ* 3.80 and 4.24 (2s, 2H, -CH_2_-), 4.82 (s, 2H, -CH_2_-), 6.56–6.66 (m, 3H, ArHs), 7.00–7.13 (m, 4H, ArHs), 7.39 (t, *J* = 8.0 Hz, 1H, ArH), 7.83 and 7.93 (2s, 1H, ArH), 8.44 and 8.64 (2s, 1H, -CH=N-), 11.57 and 11.61 (2s, D_2_O exchangeable, 1H, -NH-). ^13^CNMR (DMSO-*d*_6_) *δ* 44.31, 46.29 (-CH_2_-), 65.29, 65.33 (-CH_2_-), 112.78, 113.08, 113.17, 116.51, 116.86. 121.75, 121.78, 123.05, 126.12, 126.19, 126.32, 129.32, 129.37, 131.58, 139.65, 142.64, 148.77, 148.85, 156.64, 156.70, 167.39, 160.47, 170.53 (>C=O), 171.89 (-CO_2_H). Anal. Calcd. For: C_17_H_17_N_3_O_4_ (327.43): C, 62.38; H, 5.23; N, 12.84; found: C, 62.45, H, 5.31, N, 12.95.

2-(2-((2-(4-Tolylglycyl)hydrazineylidene)methyl)phenoxy)acetic acid (**10b**)

White powder, 78% yield; mp 236–238 °C; IR (KBr) *ν*_max_/cm^−1^ 1732 (>C=O), 2229–3500 (-CO_2_H), 3163 (>NH), 3421 (>NH). ^1^HNMR (DMSO-*d*_6_) *δ* 2.16 (s, 3H, CH_3_), 3.76 and 4.21 (2s, 2H, -CH_2_-), 4.82 (s, 2H, -CH_2_-), 6.51–6.57 (m, 2H, ArHs), 6.90–7.94 (m, 2H, ArHs), 7.00–7.08 (m, 2H, ArH), 7.39 (t, *J* = 8.8 Hz, 1H, ArH), 7.83 and 7.92 (2d, *J* = 8.8/8.8 Hz, 1H, ArH), 8.43 and 8.63 (2s, 1H, -CH=N-), 11.55 and 11.57 (2s, D_2_O exchangeable, 1H, -NH-). ^13^CNMR (DMSO-*d*_6_) *δ* 20.57 (-CH_3_). 44.60, 46.68 (-CH_2_-), 65.30, 65.33 (-CH_2_-), 112.92, 113.07, 113.16, 121.74, 121.77, 123.05, 124.90, 125.31, 126.11, 126.17, 129.76, 129.80, 131.56, 131.74, 139.61, 142.61, 146.50, 146.60, 156.63, 156.70, 170.47, 170.53 (>C=O), 172.01 (-CO_2_H). Anal. Calcd. For: C_17_H_17_N_3_O_4_ (327.43): C, 62.38; H, 5.23; N, 12.84; found: C, 62.45, H, 5.31, N, 12.95. Anal. Calcd. For: C_18_H_19_N_3_O_4_ (341.37): C, 63.33; H, 5.61; N, 12.31; found; C, 63.41, H, 5.72, N, 12.42.

2-(2-((2-((4-Chlorophenyl)glycyl)hydrazineylidene)methyl)phenoxy)acetic acid (**10c**)

White powder, 79% yield; mp 240–242 °C; IR (KBr) ν_max_/cm^−1^ 1732 (>C=O), 2190–3500 (-CO_2_H), 3167 (>NH), 3421 (>NH). ^1^HNMR (DMSO-*d*_6_) *δ* 3.80 and 4.24 (2s, 2H, -CH_2_-), 4.82 (s, 2H, -CH_2_-), 5.98 and 6.21 (2s, D_2_O exchangeable, 1H, -NH-), 6.59–6.66 (m, 2H, ArHs), 7.00–7.07 (m, 2H, ArHs), 7.13 (t, *J* = 8.0 Hz 2H, ArHs), 7.39 (t, *J* = 8.0 Hz, 1H, ArH), 7.83 and 7.92 (2d, *J* = 8.8/8.8 Hz, 1H, ArH), 8.43 and 8.63 (2s, 1H, -CH=N-), 11.56 and 11.61 (2s, D_2_O exchangeable, 1H, -NH-). ^13^CNMR (DMSO-*d*_6_) *δ* 44.36, 46.14 (-CH_2_-), 65.29, 65.33 (-CH_2_-), 113.07, 113.18, 114.15, 119.68, 120.08, 121.75, 123.01, 126.12, 126.23, 128.96, 129.05, 131.59, 131.79, 139.72, 142.70, 147.78, 147.89 156.64, 167.01, 170.47, 170.53 (>C=O), 171.57 (-CO_2_H). Anal. Calcd. For: C_17_H_16_ClN_3_O_4_ (361.78): C, 56.44; H, 4.46; N, 11.62; found; C, 56.53, H, 4.55, N, 11.72.

2-(4-Bromo-2-((2-(phenylglycyl)hydrazineylidene)methyl)phenoxy)acetic acid (**10d**)

White powder, 76% yield; mp 231–233 °C; IR (KBr) *ν*_max_/cm^−1^ 1720 (>C=O), 2306–3500 (-CO_2_H), 3167 (>NH), 3425 (>NH). ^1^HNMR (DMSO-*d*_6_) *δ* 3.81 and 4.27 (2s, 2H, -CH_2_-), 4.83 (d, *J* = 8.0 Hz, 2H, -CH_2_-), 6.55–6.65 (m, 3H, ArHs), 7.01–7.04 (m, 1H, ArH), 7.10 (q, *J* = 8.0 Hz, 2H, ArHs), 7.54 (d, *J* = 8.0 Hz, 1H, ArH), 7.89 and 8.00 (2d, 1H, ArH), 8.35 and 8.57 (2s, 1H, -CH=N-), 11.63 and 11.73 (2s, D_2_O exchangeable, 1H, -NH-). ^13^CNMR (DMSO-*d*_6_) *δ* 44.28, 46.37 (-CH_2_-), 65.57, 65.61 (-CH_2_-), 112.79, 113.50, 113.62, 115.69, 115.86, 116.49, 116.61, 125.32, 128.02, 128.10, 129.29, 129.37. 133.71, 133.81, 138.07, 141.00, 148.74, 148.93, 155.84, 155.87, 167.69, 170.21, 170.27 (>C=O), 172.13 (-CO_2_H). Anal. Calcd. For: C_17_H_16_BrN_3_O_4_ (406.24): C, 50.26; H, 3.97; N, 10.34; found; C, 50.31; H, 4.02; N, 10.39.

2-(4-Bromo-2-((2-(*p*-tolylglycyl)hydrazineylidene)methyl)phenoxy)aceticacid (**10e**)

White powder, 68% yield; mp 239–240 °C; IR (KBr) ν_max_/cm^−1^ 1720 (>C=O), 2152–3500 (-CO_2_H), 3167 (>NH), 3421 (>NH). ^1^HNMR (DMSO-*d*_6_) *δ* 2.16 (s, 1H, CH_3_), 3.77 and 4.27 (2s, 2H, -CH_2_-), 4.84 (d, *J* = 8.0 Hz, 2H, -CH_2_-), 6.53 (q, *J* = 8.8 Hz, 2H, ArHs), 6.89 and 7.99 (2d, 2H, ArHs), 7.01–7.04 (m, 2H, ArHs), 7.54 (d, *J* = 8.0 Hz, 1H, ArH), 7.53 and 7.56 (2d, 1H, ArH), 8.34 and 8.56 (2s, 1H, -CH=N-), 11.61 and 11.69 (2s, D_2_O exchangeable, 1H, -NH-). ^13^CNMR (DMSO-*d*_6_) *δ* 2.57 (CH_3_), 44.57, 46.75 (-CH_2_-), 65.56, 65.59 (-CH_2_-), 112.93, 113.50, 113.62, 115.62, 115.85, 124.87, 125.32, 125.34, 128.01, 128.08, 129.74, 128.80, 129.74, 129.80, 133.70, 133.80, 140.97, 146.46, 146.68, 155.83, 155.86, 170.20, 170.27 (>C=O), 172.27 (-CO_2_H). Anal. Calcd. For: C_18_H_18_BrN_3_O_4_ (420.26): C, 51.44; H, 4.32; N, 10.00; found; C, 51.47; H, 4.38; N, 10.07.

2-(4-Bromo-2-((2-((4 chlorophenyl)glycyl)hydrazineylidene)methyl)phenoxy) acetic acid (**10f**)

White powder, 72% yield; mp 261–262 °C; IR (KBr) ν_max_/cm^−1^ 1728 (>C=O), 2156–3600 (-CO_2_H), 3167 (>NH), 3433 (>NH). ^1^HNMR (DMSO-*d*_6_) *δ* 3.81 and 4.27 (2s, 2H, -CH_2_-), 4.84 (d, *J* = 8.0 Hz, 2H, -CH_2_-), 6.01 and 6.25 (2s, D_2_O exchangeable, 1H, -NH-), 6.63 (q, *J* = 8.8 Hz, 2H, ArHs), 7.02 (t, *J* = 8.0 Hz, 1H, ArHs), 7.12 (t, *J* = 8.8 Hz, 2H, ArHs), 7.54 and 7.55 (2d, 1H, ArH), 7.89 and 7.99 (2d, 1H, ArH), 8.34 and 8.56 (2s, 1H, -CH=N-), 11.63 and 11.74 (2s, D_2_O exchangeable, 1H, -NH-). ^13^CNMR (DMSO-*d*_6_) *δ* 44.33, 46.20 (-CH_2_-), 65.56, 65.60 (-CH_2_-), 13.50, 113.62 114.14. 114.17 115.67, 119.65, 120.1, 125.28, 128.01, 128.14, 129.05, 133.73, 138.14, 141.06, 147.74, 147.98, 155.84, 155.88, 167.31, 170.21, 170.27 (>C=O), 171.83 (-CO_2_H). Anal. Calcd. For: C_17_H_15_BrClN_3_O_4_ (440.68): C, 46.33; H, 3.43; N, 9.54; found; C, 46.43; H, 3.49; N, 9.58.

2-(2-((2-(Benzoylglycyl)hydrazineylidene)methyl)phenoxy)acetic acid (**13a**)

White powder, 72% yield; mp 210–211 °C; IR (KBr) ν_max_/cm^−1^ 1735 (>C=O), 2299–3500 (-CO_2_H), 3178 (>NH), 3305 (>NH). ^1^HNMR (DMSO-*d*_6_) *δ* 3.99 and 4.44 (2d, 2H, -CH_2_-), 4.83 (s, 2H, -CH_2_-), 7.03 (q, *J* = 8.8 Hz, 2H, ArHs), 7.39 (t, *J* = 8.0 Hz, 1H, ArH), 7.49–7.58 (m, 3H, ArH), 7.83–7.93 (m, 3H, ArHs), 8.43 and 8.64 (2s, 1H, -CH=N-), 8.71 and 8.89 (2t, D_2_O exchangeable, 1H, -NH-), 11.57 and 11.64 (2s, D_2_O exchangeable, 1H, -NH-). ^13^CNMR (DMSO-*d*_6_) *δ* 41.28, 42.44 (-CH_2_-), 65.31, 65.34 (-CH_2_-), 113.12, 113.19, 121.77, 123.02, 123.9, 126.03. 126.13, 127.73, 127.83, 128.82, 131.57, 131.75, 131.81, 131.88, 134.36, 134,57, 139.50, 142.55, 156.62, 156.70, 166.02, 167.00, 167.06, 170.48 (>C=O), 170.53 (>C=O), 170.75 (-CO_2_H). Anal. Calcd. For: C_18_H_17_N_3_O_5_ (355.35): C, 60.84; H, 4.82; N, 11.83; found; C, 60.92, H, 4.90, N, 11.92.

2-(2-((2-(Benzoylglycyl)hydrazineylidene)methyl)-4-bromophenoxy)acetic acid (**13b**)

White powder, 67% yield; mp 248–250 °C; IR (KBr) ν_max_/cm^−1^ 1728 (>C=O), 2160–3599 (-CO_2_H), 3170 (>NH), 3302 (>NH). ^1^HNMR (DMSO-*d*_6_) *δ* 4.00 and 4.46 (2s, 2H, -CH_2_-), 4.85 (s, 2H, -CH_2_-), 7.03 (t, *J* = 8.8 Hz, 1H, ArH), 7.49–7.59 (m, 4H, ArHs), 7.90–7.97 (m, 3H, ArHs), 8.35 and 8.57 (s2, 1H, -CH=N-), 8.74 and 8.91 (2t, D_2_O exchangeable, 1H, -NH-), 11.65 and 11.76 (s, D_2_O exchangeable, 1H, -NH-). ^13^CNMR (DMSO-*d*_6_) *δ* 41.33, 42.49 (-CH_2_-), 65.58, 65.62 (-CH_2_-), 113.52, 113.63, 115.75, 115.75 125.28, 125.37, 127.74, 127.84, 127.94, 128.01, 128.81, 131.81, 131.90, 133.70, 133.83 134.32, 134.57, 137.89, 140.88, 155.82, 155.88, 166.27, 167.09, 170.22 (>C=O), 170.27 (>C=O), 170.93 (-CO_2_H). Anal. Calcd. For: C_18_H_16_BrN_3_O_5_ (434.25): C, 49.79; H, 3.71; N, 9.68; found: C, 49.87; H, 3.75; N, 9.73.

### 3.2. Biological Activity

#### 3.2.1. In Vitro Assessment [52]

In vitro enzymatic analysis was performed as reported (Supplementary Materials, page 52).

#### 3.2.2. Animals

The research ethical committee at the Faculty of Pharmacy, Egyptian Russian University, granted approval for the experimental protocol (ERUFP-PC-24-001) (Supplementary Materials, page 52).

#### 3.2.3. Chemicals

Reference drugs were acquired in tablets and assayed as mentioned (Supplementary Materials, page 52).

#### 3.2.4. In Vivo Assessment [53]

The carrageenan paw edema test was conducted following established procedures as detailed (Supplementary Materials, page 53).

#### 3.2.5. Assessment of Inflammatory Biomarkers Using ELISA [54]

ELISA assessment assays were conducted as cited (Supplementary Materials, page 53).

#### 3.2.6. Analgesic Activity [55]

The hot plate latency test was performed according to the following procedures (Supplementary Materials, page 54).

#### 3.2.7. Histopathological Examination [56]

The histopathological assessments were performed according to the following procedures (Supplementary Materials, page 54).

#### 3.2.8. Toxicity Assessment

##### Assessment of liver and kidney function

These assessments were conducted following the manufacturer’s instructions (Supplementary Materials, page 55).

##### Evaluation of Ulcerogenic Effects

The assessment of ulcerogenic effects was conducted following established procedures [42,57] (Supplementary Materials, page 55).

#### 3.2.9. Statistical Analysis

The data were statistically analyzed using GraphPad Prism 9.5.1 Demo (GraphPad Software, San Diego, CA, USA). (Supplementary Materials, page 56).

#### 3.2.10. Molecular Modeling Study

Docking studies are performed using AutoDockVina 1.5.7 (Supplementary Materials, page 56). 

#### 3.2.11. In Silico Drug-Likeness and ADME Prediction [58]

The ADME study was performed using the SwissADME server (Supplementary Materials, page 57).

## 4. Conclusions

This project aims to create specific COX-2 inhibitor analogs similar to celecoxib and mefenamic acid and then evaluate them in vitro to determine the most effective molecules. The most powerful compounds, **5d**–**f**, **7b**, and **10c**–**f**, were tested in vivo to determine their effectiveness. Compounds **5f** and **7b**, identified as the most potent, underwent additional investigations, revealing significant anti-inflammatory effects and a safe profile in a carrageenan-induced paw edema inflammation model. Both compounds showed strong anti-inflammatory effects by reducing paw edema thickness and the percentage of paw weight gain, similarly to the reference medications mefenamic acid **V** and celecoxib **IX**. Compounds **5f** and **7b** showed potential as dual anti-inflammatory and analgesic medicines based on their promising analgesic activities in the hot plate latency test. No substantial adverse effects on liver and renal function were found in the toxicity study of both compounds, confirming their safety profile. Additionally, the assessment of ulcer-causing effects showed that both compounds had a significantly decreased likelihood of causing gastrointestinal ulcers compared to mefenamic acid, a commonly used anti-inflammatory pain reliever renowned for its ulcer-causing effects. The results highlight the potential of compounds **5f** and **7b** as interesting candidates for further development as anti-inflammatory and analgesic drugs, requiring further exploration in preclinical and clinical trials.

## Figures and Tables

**Table 1:** In vitro inhibitory activity of designed compounds **5a**–**c**, **7a**–**b**, **10a**–**f**, and **13a**–**b** towards COX-1 and COX-2 enzymes.

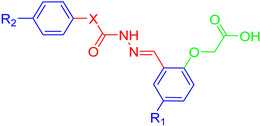						
Compound	X	R_1_	R_2_	COX-1 IC_50_ (µM)	COX-2 IC_50_ (µM)	SI
**Mefenamic acid**	–	–	–	29.9 ± 0.09	1.98 ± 0.02	15.10
**Celecoxib**	–	–	–	14.93 ± 0.12	0.05 ± 0.02	298.6
**5a**	0	-H	-H	10.00 ± 0.10	0.97 ± 0.06	10.31
**5b**	0	-H	-CH_3_	7.50 ± 0.20	0.37 ± 0.06	20.27
**5c**	0	-H	-Cl	14.50 ± 0.20	0.13 ± 0.06	111.53
**5d**	0	-Br	-H	9.03 ± 0.15	0.08 ± 0.01 ^a^	112.87
**5e**	0	-Br	-CH_3_	7.00 ± 0.20	0.07 ± 0.01 ^a^	100.00
**5f**	0	-Br	-Cl	8.00 ± 0.20	0.06 ± 0.01 ^a^	133.34
**7a**	-CH_2_-	-H	-H	10.00 ± 0.20	0.13 ± 0.06	76.92
**7b**	-CH_2_-	-Br	-H	5.93 ± 0.12	0.06 ± 0.01 ^a^	84.71
**10a**	-NH-CH_2_-	-H	-H	9.50 ± 0.20	0.50 ± 0.10	19.00
**10b**	-NH-CH_2_-	-H	-CH_3_	4.97 ± 0.06	0.33 ± 0.06	15.06
**10c**	-NH-CH_2_-	-H	-Cl	5.57 ± 0.12	0.07 ± 0.01 ^a^	79.57
**10d**	-NH-CH_2_-	-Br	-H	7.00 ± 0.20	0.08 ± 0.01 ^a^	87.50
**10e**	-NH-CH_2_-	-Br	-CH_3_	4.07 ± 0.12	0.06 ± 0.01 ^a^	67.83
**10f**	-NH-CH_2_-	-Br	-Cl	4.97 ± 0.06	0.09 ± 0.01 ^a^	55.22
**13a**	-(C=O)-NH-CH_2_-	-H	-H	8.43 ± 0.12	0.23 ± 0.06	36.65
**13b**	-(C=O)-NH-CH_2_-	-Br	-H	9.93 ± 0.12	0.13 ± 0.06	76.38

^a^ The data, which are shown as the mean ± SEM (n = 3), do not show any statistical significance when compared to celecoxib and mefenamic acid, with a p-value greater than 0.05 as determined by one-way ANOVA followed by Tukey and a post hoc test. With a red color scheme and strong lettering, the majority of active chemicals are shown.

**Table 2:** Paw thickness difference at hourly intervals (and percentage of inhibition) and the percentage of paw weight increase.

	Paw Thickness Difference at Hourly Intervals (mm) Percentage of Inhibition (%)					Paw Weight Increase (%)
	1st h	2nd h	3rd h	4th h	5th h	
**Control**	0.04 ± 0.01	0.07 ± 0.02	0.08 ± 0.03	0.02 ± 0.01	0.00 ± 0.01	0.31 ± 2.33
**Carrageenan**	1.97 ± 0.38 ^a^	3.29 ± 0.31 ^a^	4.22 ± 0.29 ^a^	5.05 ± 0.52 ^a^	5.31 ± 0.52 ^a^	94.04 ± 3.23 ^a^
**Celecoxib**	1.4 ± 0.38 ^ab^ (29.31%)	2.36 ± 0.51 ^ab^ (28.34%)	2.83 ± 0.43 ^ab^ (33%)	3.01 ± 0.57 ^ab^ (40.39%)	3.10 ± 0.23 ^ab^ (41.65%)	29.95 ± 2.20 ^ab^
**Mefenamic acid**	1.22 ± 0.19 ^ab^ (38.43%)	2.27 ± 0.11 ^ab^ (31.22%)	2.86 ± 0.11 ^ab^ (32.37%)	3.22 ± 0.19 ^ab^ (36.16%)	3.51 ± 0.07 ^ab^ (33.89%)	34.08 ± 2.27 ^ab^
**5d**	1.37 ± 0.13 ^ab^ (30.57%)	2.76 ± 0.24 ^a^ (16.19%)	3.56 ± 0.33 ^abcd^ (15.67%)	3.7 ± 0.32 ^abc^ (26.75%)	3.90 ± 0.41 ^abc^ (26.51%)	76.92 ± 3.89 ^abcd^
**5e**	1.57 ± 0.4 ^a^ (20.35%)	2.53 ± 0.48 ^ab^ (23.28%)	3.43 ± 0.10 ^abcd^ (18.71%)	3.55 ± 0.11 ^ab^ (29.59%)	3.68 ± 0.15 ^abc^ (30.72%)	47.55 ± 2.41 ^abcd^
**5f**	0.83 ± 0.15 ^ab^ (58.11%)	1.65 ± 0.29 ^abcd^ (49.85%)	2.32 ± 0.17 ^ab^ (45.05%)	2.68 ± 0.12 ^ab^ (46.9%)	2.84 ± 0.13 ^abd^ (46.51%)	29.85 ± 3.88 ^ab^
**7b**	0.65 ± 0.16 ^abc^ (67.23%)	1.08 ± 0.21 ^abcd^ (67.11%)	1.64 ± 0.13 ^abcd^ (61.23%)	1.90 ± 0.19 ^abcd^ (62.42%)	1.95 ± 0.19 ^abcd^ (63.35%)	33.07 ± 1.88 ^ab^
**10c**	1.65 ± 0.22 ^a^ (16.47%)	2.64 ± 0.33 ^ab^ (19.99%)	3.11 ± 0.29 ^ab^ (26.25%)	3.81 ± 0.19 ^abcd^ (24.47%)	4.22 ± 0.28 ^abcd^ (20.54%)	69.01 ± 4.61 ^abcd^
**10d**	1.55 ± 0.41 ^a^ (21.37%)	2.60 ± 0.21 ^ab^ (21.15%)	2.93 ± 0.37 ^ab^ (30.56%)	3.44 ± 0.37 ^ab^ (31.87%)	3.73 ± 0.34 ^abc^ (29.71%)	60.8 ± 4.27 ^abcd^
**10e**	1.79 ± 0.39 ^a^ (9.38%)	3.00 ± 0.30 ^acd^ (9.01%)	3.58 ± 0.17 ^abcd^ (15.28%)	3.98 ± 0.26 ^abcd^ (21.24%)	4.17 ± 0.24 ^abcd^ (21.42%)	64.95 ± 6.79 ^abcd^
**10f**	1.77 ± 0.27 ^a^ (10.39%)	2.85 ± 0.46 ^ad^ (13.51%)	3.24 ± 0.46 ^ab^ (23.17%)	3.63 ± 0.48 ^abc^ (28.04%)	3.78 ± 0.51 ^abc^ (28.80%)	59.16 ± 2.72 ^abcd^

The data are presented as mean ± SD and were analyzed using two-way ANOVA (for paw thickness difference) or one-way ANOVA (for paw weight increase percentage), followed by Tukey’s multiple comparisons test (n = 6). ^a^ Significantly different from the control group at *p* < 0.05, ^b^ Significantly different from the carrageenan group at *p* < 0.05, ^c^ Significantly different from the celecoxib group at *p* < 0.05, ^d^ Significantly different from the mefenamic acid group at *p* < 0.05.

**Table 3:** The analgesic activity of reference drugs and test compounds at 30, 60, 90, and 120 min.

	30 min	60 min	90 min	120 min
**Control**	6.45 ± 0.33	7.31 ± 0.14	8.47 ± 0.31	9.46 ± 0.31
**Celecoxib**	8.39 ± 0.22 ^a^	9.95 ± 0.23 ^a^	11.63 ± 0.60 ^a^	13.88 ± 0.41 ^a^
**Mefenamic acid**	9.95 ± 0.35 ^ab^	11.58 ± 0.25 ^ab^	12.83 ± 0.36 ^ab^	13.73 ± 0.42 ^a^
**5f**	8.99 ± 0.38 ^abc^	10.59 ± 0.3 ^abc^	12.48 ± 0.65 ^ab^	14.15 ± 0.38 ^a^
**7b**	8.88 ± 0.29 ^ac^	10.35 ± 0.35 ^ac^	12.09 ± 0.29 ^ac^	13.71 ± 0.31 ^a^

The data are presented as mean ± SD and were analyzed using a two-way ANOVA, followed by Tukey’s multiple comparisons test; n = 6. ^a^ Significantly different from the control group at *p* < 0.05, ^b^ Significantly different from the celecoxib group at *p* < 0.05, ^c^ Significantly different from the mefenamic acid group at *p* < 0.05.

**Table 4:** Serum ALT, AST, ALT/AST ratio, creatinine, and urea.

	ALT (IU/L)	AST (IU/L)	AST/ALT	Creatinine	Urea
**Control**	87.61 ± 6.41	95.54 ± 5.37	1.10 ± 0.12	0.69 ± 0.07	26.47 ± 1.97
**Carrageenan**	90.43 ± 6.04	100.14 ± 8.36	1.11 ± 0.14	0.73 ± 0.05	27.85 ± 1.27
**Celecoxib**	92.94 ± 5.00	101.23 ± 10.04	1.09 ± 0.08	0.73 ± 0.03	26.28 ± 3.11
**Mefenamic acid**	88.84 ± 6.09	97.51 ± 8.08	1.10 ± 0.15	0.72 ± 0.05	27.58 ± 2.08
**5f**	92.76 ± 3.83	93.69 ± 5.26	1.01 ± 0.08	0.67 ± 0.04	26.37 ± 2.48
**7b**	89.13 ± 8.53	97.59 ± 6.19	1.10 ± 0.12	0.68 ± 0.05	27.16 ± 1.53

The data are presented as mean ± SD and were analyzed using one-way ANOVA, followed by Tukey’s multiple comparisons test; n = 6. No significant differences were observed among the different groups.

**Table 5:** The ulcer numbering and severity score for all groups.

	Ulcer Number Score	Ulcer Severity Score
**Control**	0.50 ± 0.55	0.25 ± 0.27
**Carrageenan**	0.83 ± 0.75	0.50 ± 0.45
**Celecoxib**	1.00 ± 0.63	0.75 ± 0.52
**Mefenamic acid**	1.67 ± 0.82 ^a^	1.17 ± 0.61 ^a^
**5f**	0.50 ± 0.55 ^d^	0.33 ± 0.41 ^d^
**7b**	0.67 ± 0.52	0.42 ± 0.38

The data are presented as mean ± SD and were analyzed using one-way ANOVA, followed by Tukey’s multiple comparisons test (n = 6). ^a^ Significantly different from the control group at *p* < 0.05, ^d^ Significantly different from the mefenamic acid group at *p* < 0.05.

**Table 6:** The binding score, amino acid interactions, and bond lengths of compounds **5f** and **7b** within the active sites of the COX-2 enzyme.

Compound	Binding Score (kcal/mol)	Interaction	Bond Length (A^o^)	Ligand Atom	Residue	Enzyme	PDB ID
**Celecoxib**	−11.0	H-Bond	1.98	O of SO_2_NH_2_	His90	COX-2	1CX2
		H-Bond	2.24	O of SO_2_NH_2_	Arg513		
		H-Bond	2.26	F of CF3	Arg120		
**5f**	−8.7	H-Bond	2.89	CO of COO^−^	His90	COX-2	1CX2
		H-Bond	3.07	O of COO^−^	Arg513		
		H-Bond	3.12	O of COO^−^	Tyr355		
		H-Bond	2.28	CO of CONH	Tyr385		
**7b**	−9.0	H-Bond	2.30	O of COO^−^	His90	COX-2	1CX2
		H-Bond	2.14	O of COO^−^	Arg513		
		Pi-sigma	3.84	phenoxy ring	leu352		

## Data Availability

Data are contained within the article and Supplementary Materials.

## Supplementary Materials

The following supporting information can be downloaded at: https://www.mdpi.com/article/10.3390/molecules29061309/s1, Figure S1: IC_50_ curves against COX-2; Figure S2 and Table S1: Virtual ADME assessment; Figure S3: In vivo assessment of designed compounds; Figures S4 and S5: Molecular docking; Figures S6–S27: ^1^H NMR and ^13^C NMR spectra of **5a**–**f**, **7a**, **b**, **10a**–**f**, and **13a**, **b**; Pages 51–56: Experimental procedures and instruments.
