# Supplementary material for: Design, Synthesis, and Biological Evaluation of Novel Phenoxy Acetic Acid Derivatives as Selective COX-2 Inhibitors Coupled with Comprehensive Bio-Pharmacological Inquiry, Histopathological Profiling, and Toxicological Scrutiny
Source: Molecules. 2024 Mar 15;29(6):1309. doi: 10.3390/molecules29061309 (PMC10974743; doi:10.3390/molecules29061309)
Supplement: Supplementary file 1 [file molecules-29-01309-s001.zip › molecules-2907565-supplementary.pdf]

Supplementary data

Design, synthesis and biological evaluation of novel phenoxy acetic acid derivatives as selective COX-2 inhibitors coupled with comprehensive bio-pharmacological inquiry, histopathological profiling, and toxicological scrutiny

Najla A. Alshaye [a], Mohamed K. Elgohary[b,] *, Mahmoud S. Elkotamy[b], Hatem A. Abdel-Aziz[c,] *

[1] Department of Chemistry, College of Science, Princess Nourah bint Abdulrahman University, P.O. Box 84428, Riyadh 11671, Saudi Arabia; naalshaye@pnu.edu.sa

[2] Pharmaceutical Chemistry Department, Faculty of Pharmacy, Egyptian-Russian University, Badr City, Cairo, 11829, Egypt; mohamed-elgohary@eru.edu.eg (M.K.E.); mahmoud-elkotamy@eru.edu.eg (M.S.E.)

[3] Applied Organic Chemistry Department, National Research Center, Dokki, Cairo, 12622, Egypt; hatem_741@yahoo.com

* Correspondence: mohamed-elgohary@eru.edu.eg (M.K.E.); hatem_741@yahoo.com (H.A.A.)

Contents

**IC_{50} curves against COX-2 isozyme**	**4**
**Figure S1:** IC_{50} curves of potent compounds **5d-f, 7b** and **9c-f**	**5**
**Virtual ADME assessment**	**7**
**Figure S2.** Computational ADME prediction study of human intestinal absorption	**8**
**Table S1.** ADME and pharmacological aspects prediction for compounds (**5d-f, 7b** and **10c-f**)	**8**
***In vivo* assessment of designed compounds**	**9**
**Figure S3:** Impact of test compounds on the paw weight percentage in a carrageenan induced paw edema inflammation model	**10**
**Molecular docking survey**	**11**
**Figure S4:** Superimposition of co-crystalized celecoxib (purple) and redocked celecoxib (Cyan) for validation of docking protocol**.**	**12**
**Figure S5:** 3D diagram of the binding mode of most active compound **5f** (left) and **7b** (right) on the active site of COX-2 enzyme	**13**
**^{1}HNMR and ^{13}CNMR spectra**	**14**
**Figure S6.** ^{1}H NMR of compound **5a**……………………………………………………..……	**15**
**Figure S7**. ^{13}C NMR of compound **5a**..………………………………………………… ……	**16**
**Figure S6.** ^{1}H NMR of compound **5b**……………………………………………………..……	**17**
**Figure S7**. ^{13}C NMR of compound **5b**..………………………………………………… ……	**18**
**Figure S8.** ^{1}H NMR of compound **5c**…………………………………………………………	**19**
**Figure S8.** D_2O of compound **5c**…………………………………………………………………	**20**
**Figure S9.** ^{13}C NMR of compound **5c**…………………..………………………………..……	**21**
**Figure S10.** ^{1}H NMR of compound **5d**………………………………………………….………	**22**
**Figure S11.** ^{13}C NMR of compound **5d**……………………………………………………………	**23**
**Figure S12.** ^{1}H NMR of compound **5e**…………….……………………………………….……	**24**
**Figure S13.** ^{13}C NMR of compound **5e**…………….……………………………………….……	**25**
**Figure S12.** ^{1}H NMR of compound **5f**…………….……………………………………….……	**26**
**Figure S13.** ^{13}C NMR of compound **5f**…………….……………………………………….……	**27**
**Figure S14.** ^{1}H NMR of compound **7a**………….....…………………………………..…………	**28**
**Figure S15.** ^{13}C NMR of compound **7a**………………………………………..……………..………	**29**
**Figure S16.** ^{1}H NMR of compound **7b**……………………………………..……………...……	**30**
**Figure S16.** D_2O of compound **7b**……………………………………..…………..…………	**31**
**Figure S17.** ^{13}C NMR of compound **7b**……………………………………………………………	**32**
**Figure S18.** ^{1}H NMR of compound **10a**………………………………………………...……	**33**

**Figure S19.** ^{13}C NMR of compound **10a**……………………………………………	**34**
**Figure S20.** ^{1}H NMR of compound **10b**……………………………………………	**35**
**Figure S21.** ^{13}C NMR of compound **10b**……………………………………………	**36**
**Figure S22.** ^{1}H NMR of compound **10c**. ……………………………………………	**37**
**Figure S23.** ^{13}C NMR of compound **10c**. ……………………………………………	**38**
**Figure S22.** ^{1}H NMR of compound **10d**. ……………………………………………	**39**
**Figure S23.** ^{13}C NMR of compound **10d**. ……………………………………………	**40**
**Figure S22.** ^{1}H NMR of compound **10e**. ……………………………………………	**41**
**Figure S23.** ^{13}C NMR of compound **10e**. ……………………………………………	**42**
**Figure S22.** ^{1}H NMR of compound **10f**. ……………………………………………	**43**
**Figure S22.** D_2O of compound **10f**. ……………………………………………	**44**
**Figure S23.** ^{13}C NMR of compound **10f**. ……………………………………………	**45**
**Figure S24.** ^{1}H NMR of compound **13a**. ……………………………………………	**46**
**Figure S24.** D_2O of compound **13a**. ……………………………………………	**47**
**Figure S25.** ^{13}C NMR of compound **13a**. ……………………………………………	**48**
**Figure S26.** ^{1}H NMR of compound **13b**. ……………………………………………	**49**
**Figure S27.** ^{13}C NMR of compound **13b**. ……………………………………………	**50**
**Experimental procedures and instruments**	**51**
Chemistry instruments……………………………………………	**52**
In-vitro cyclooxygenase (COX-1/COX-2) inhibition assay……………………………	**52**
Animals ……………………………………………	**52**
Chemicals ……………………………………………	**52**
In-vivo anti-inflammatory activity ……………………………………………	**53**
Assessment of inflammatory biomarkers using ELISA……………………………	**53**
Analgesic activity……………………………………………	**54**
Histopathological examination……………………………………………	**54**
Assessment of liver and kidney function……………………………………………	**55**
Evaluation of ulcerogenic effects……………………………………………	**55**
Docking study ……………………………………………	**56**
Statistical analyses ……………………………………………	**56**
In silico predictive ADME study ……………………………………………	**57**

IC_{50} curves against COX-2 isozyme

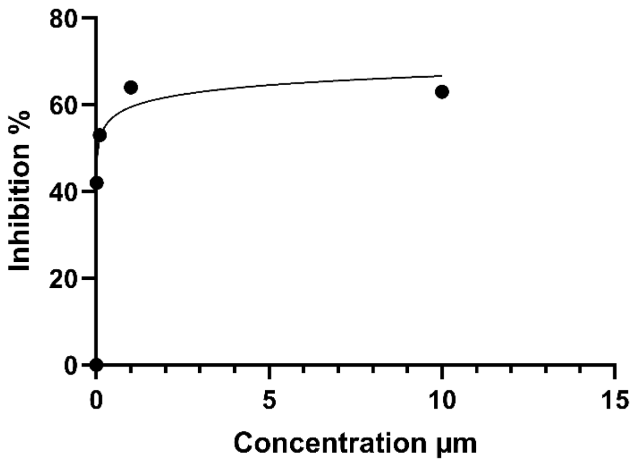

5d

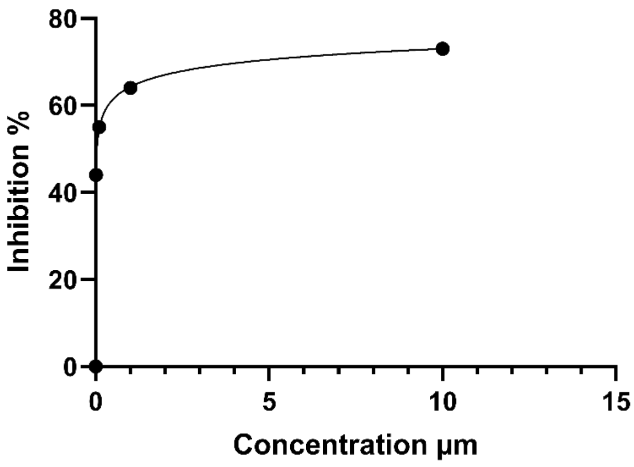

5e

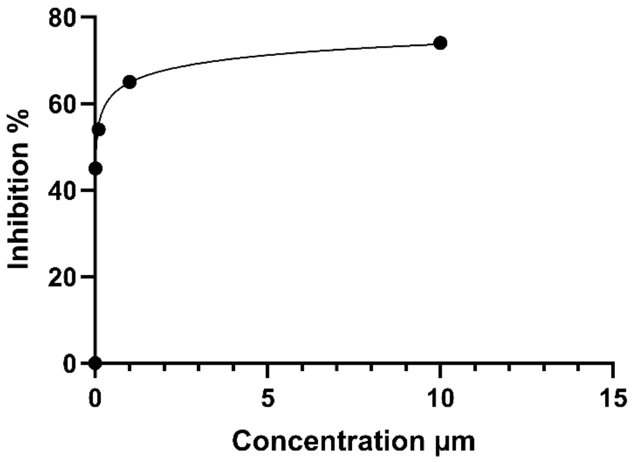

5f

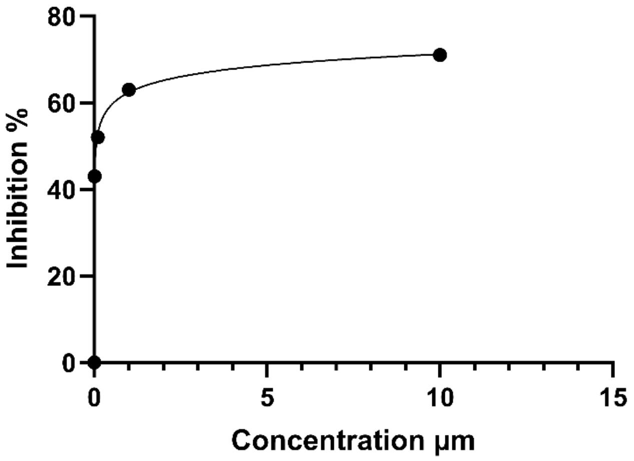

7b

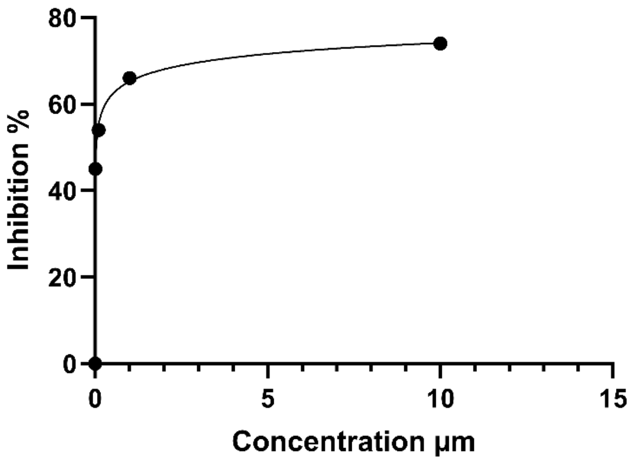

9c

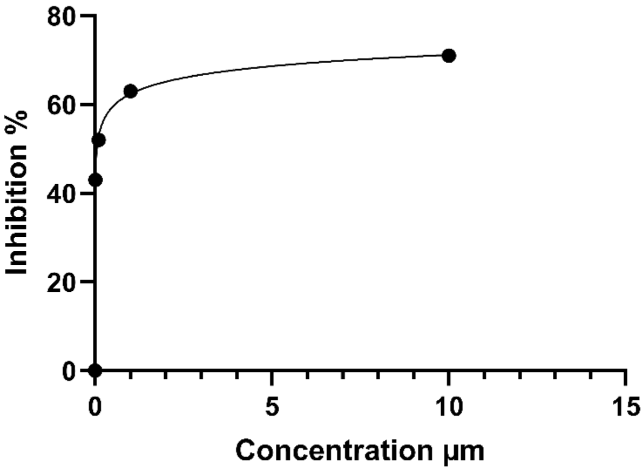

9d

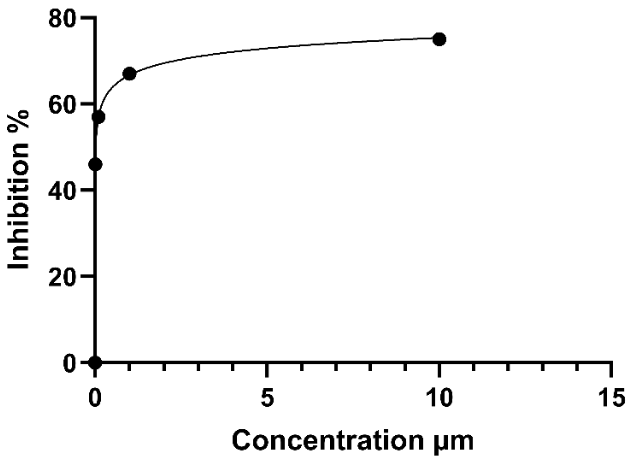

9e

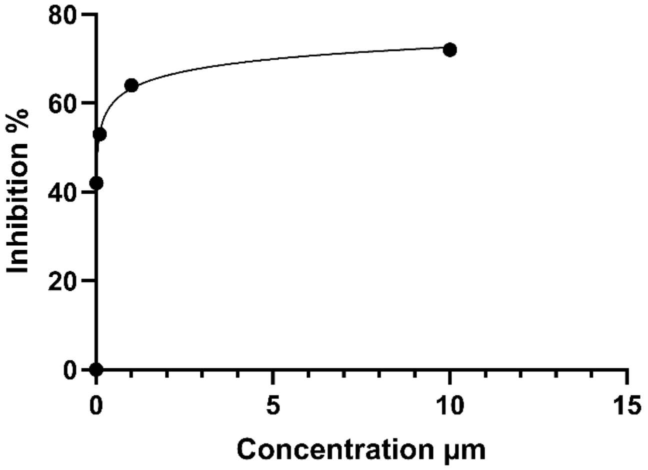

9f

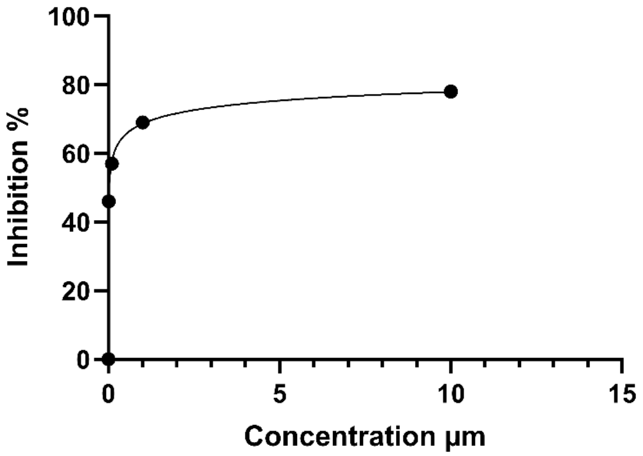

Celecoxib IX

Figure S1: IC_{50} curves of potent compounds **5d-f, 7b** and **9c-f**

Virtual ADME assessment

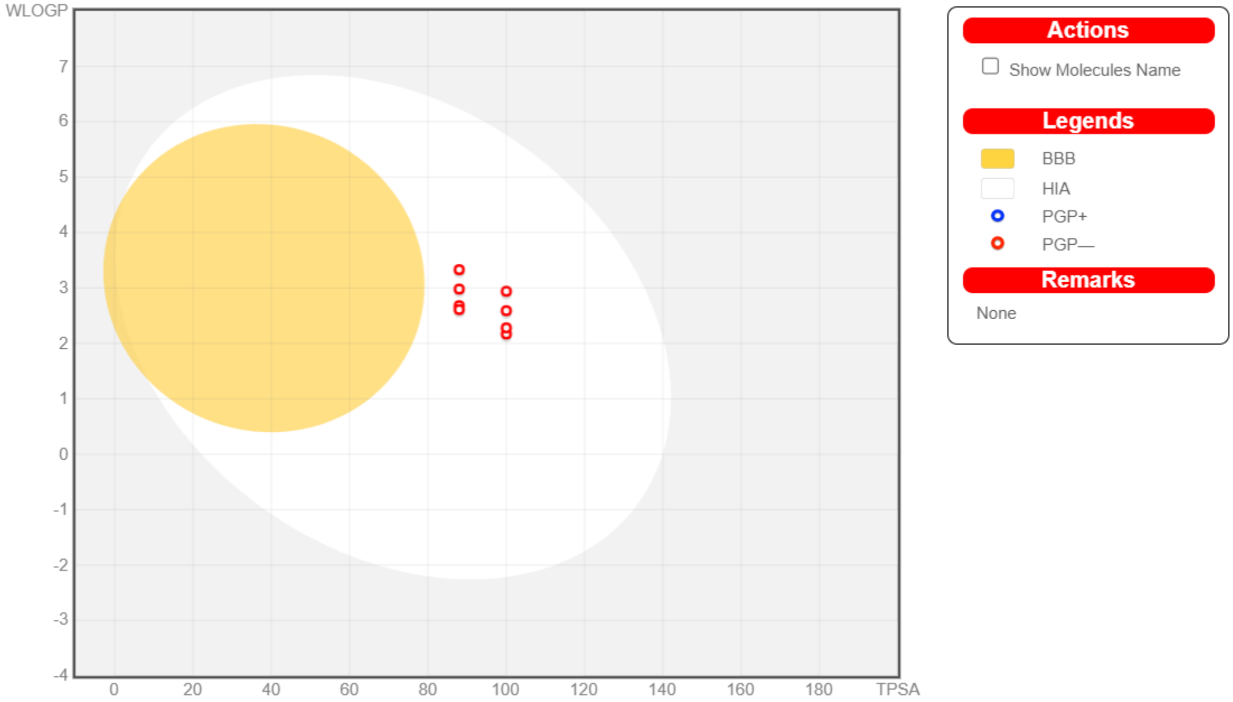

Figure S2. Computational ADME prediction study of human intestinal absorption

Table S1. ADME and pharmacological aspects prediction for compounds (5d-f, 7b and 10c-f)

properties	M.wt	H. Bond Donor	H. Bond Acceptor	Log *P*	TPSA	Rotatable bonds	Lipinski violations	Veber violations	P.gp substrate
**5d**	377.19	2	5	2.4	87.99	7	0	0	No
**5e**	391.22	2	5	2.57	87.99	7	0	0	No
**5f**	411.63	2	5	2.6	87.99	7	0	0	No
**7b**	391.22	2	5	2.39	87.99	8	0	0	No
**10c**	361.78	3	5	2.37	100.02	9	0	0	No
**10d**	406.23	3	5	2.26	100.02	9	0	0	No
**10e**	420.26	3	5	2.63	100.02	9	0	0	No
**10f**	440.68	3	5	2.68	100.02	9	0	0	No
**Celecoxib IX**	381.37	2	5	3.40	86.36	0	0	0	No

M.wt: molecular weight, Log*P*: Octanol-water partition coefficient

TPSA: Topological polar surface area.

In vivo assessment of designed compounds

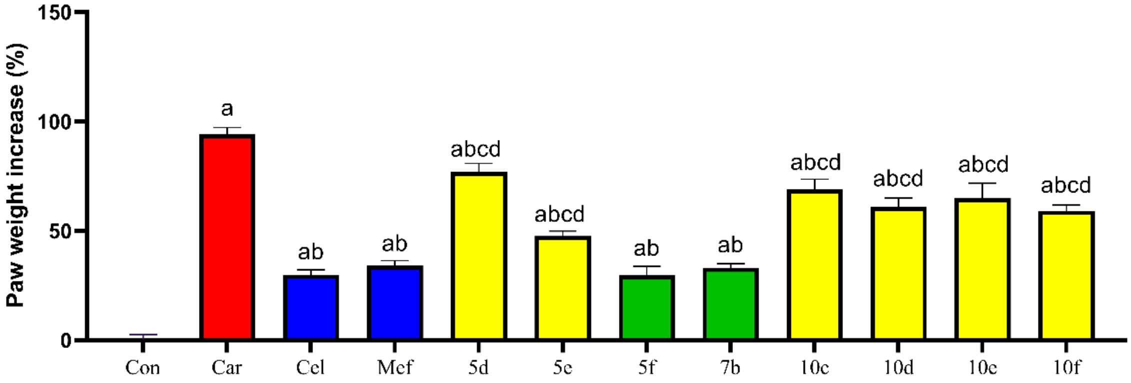

Figure S3: Impact of test compounds on the paw weight increase percentage in a carrageenan induced paw edema inflammation model. The data are presented as mean ± SD and were analyzed using one-way ANOVA, followed by Tukey's multiple comparisons test; n = 6. [a] Significantly different from control group at $p < 0.05$, [b] Significantly different from carrageenan group at $p < 0.05$, [c] Significantly different from celecoxib group at $p < 0.05$, [d] Significantly different from mefenamic acid group at $p < 0.05$. Con: control, Car: carrageenan, Cel: celecoxib, Mef: mefenamic acid.

Molecular docking survey

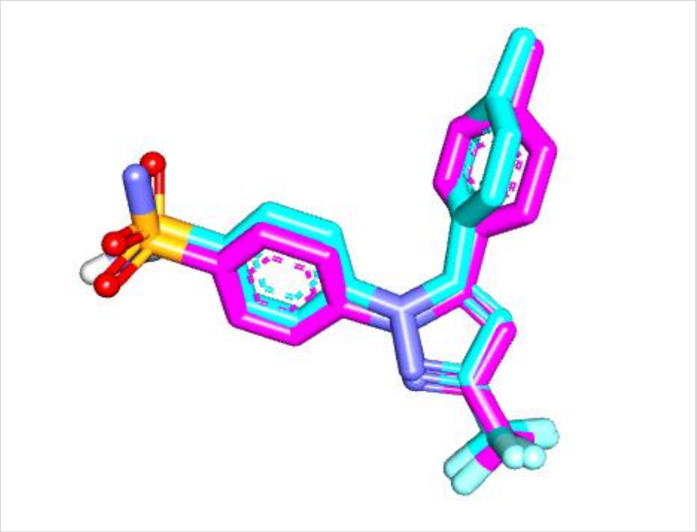

mode	affinity (kcal/mol)	dist from best mode rmsd l.b.	rmsd u.b.
1	-11.0	0.000	0.000
2	-8.8	4.943	6.434
3	-8.5	3.875	5.950
4	-8.2	4.874	7.274

Writing output ... done.

Figure S4: Superimposition of co-crystalized celecoxib **IX** (purple) and redocked celecoxib (Cyan) for validation of docking protocol.

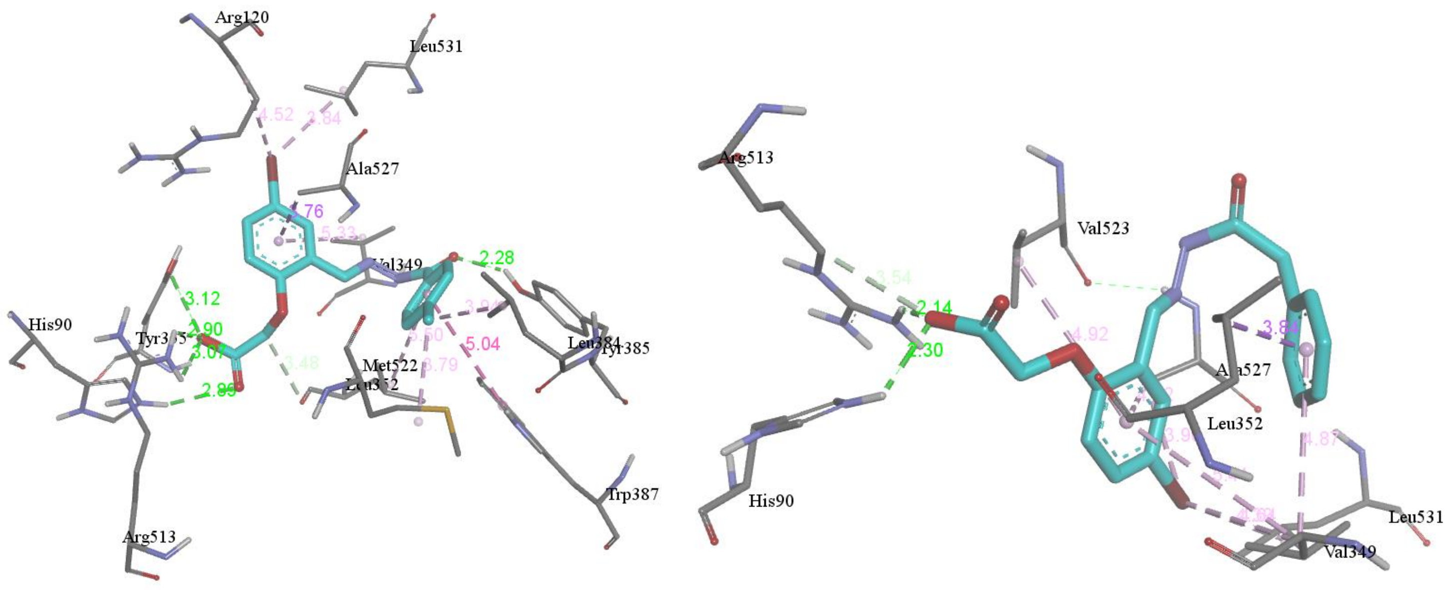

Figure S5: 3D diagram of the binding mode of most active compound **5f** (left) and **7b** (right) on the active site of COX-2 enzyme.

^{1}HNMR and ^{13}CNMR spectra

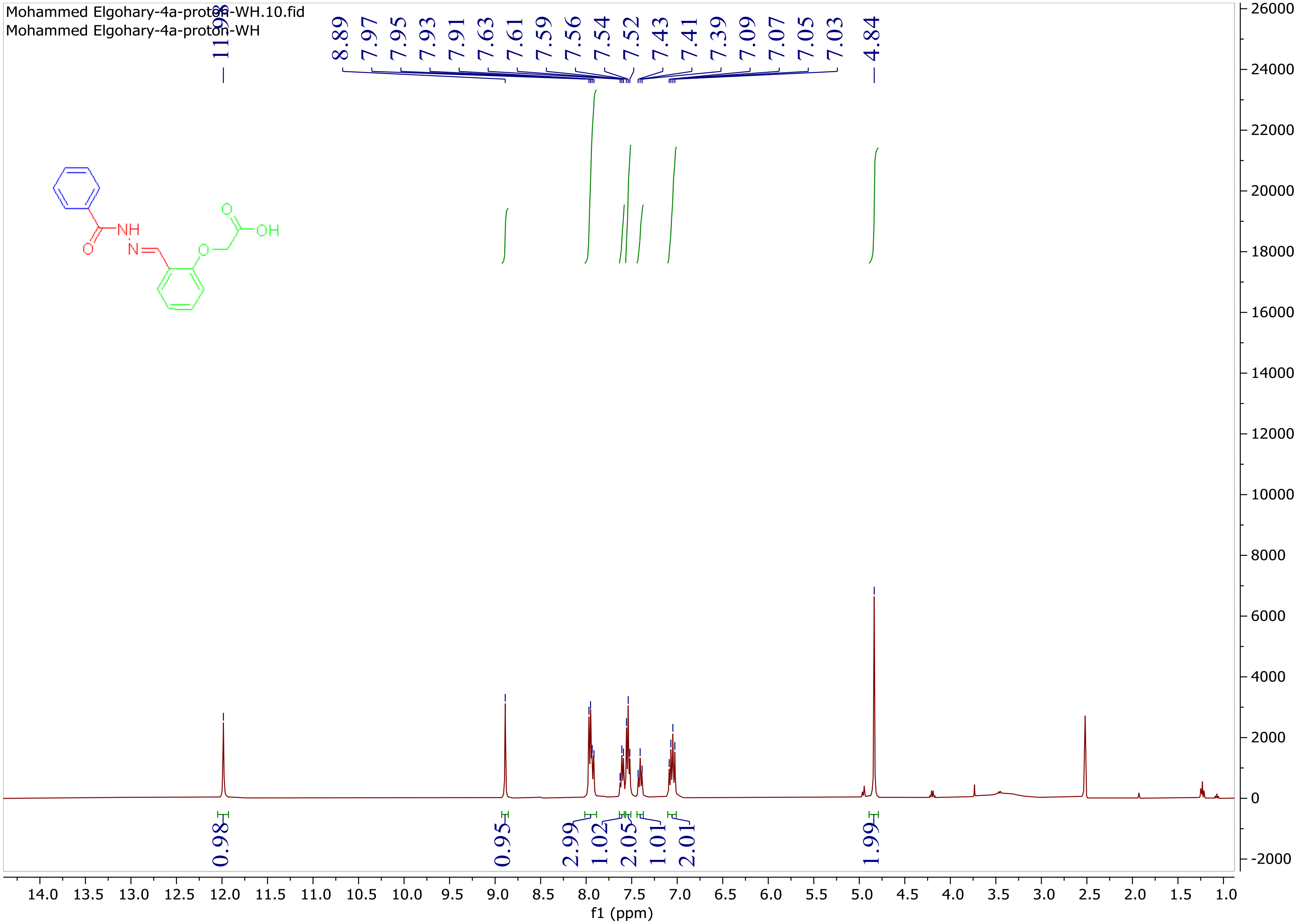

Figure S6. ^{1}H NMR of compound **5a**

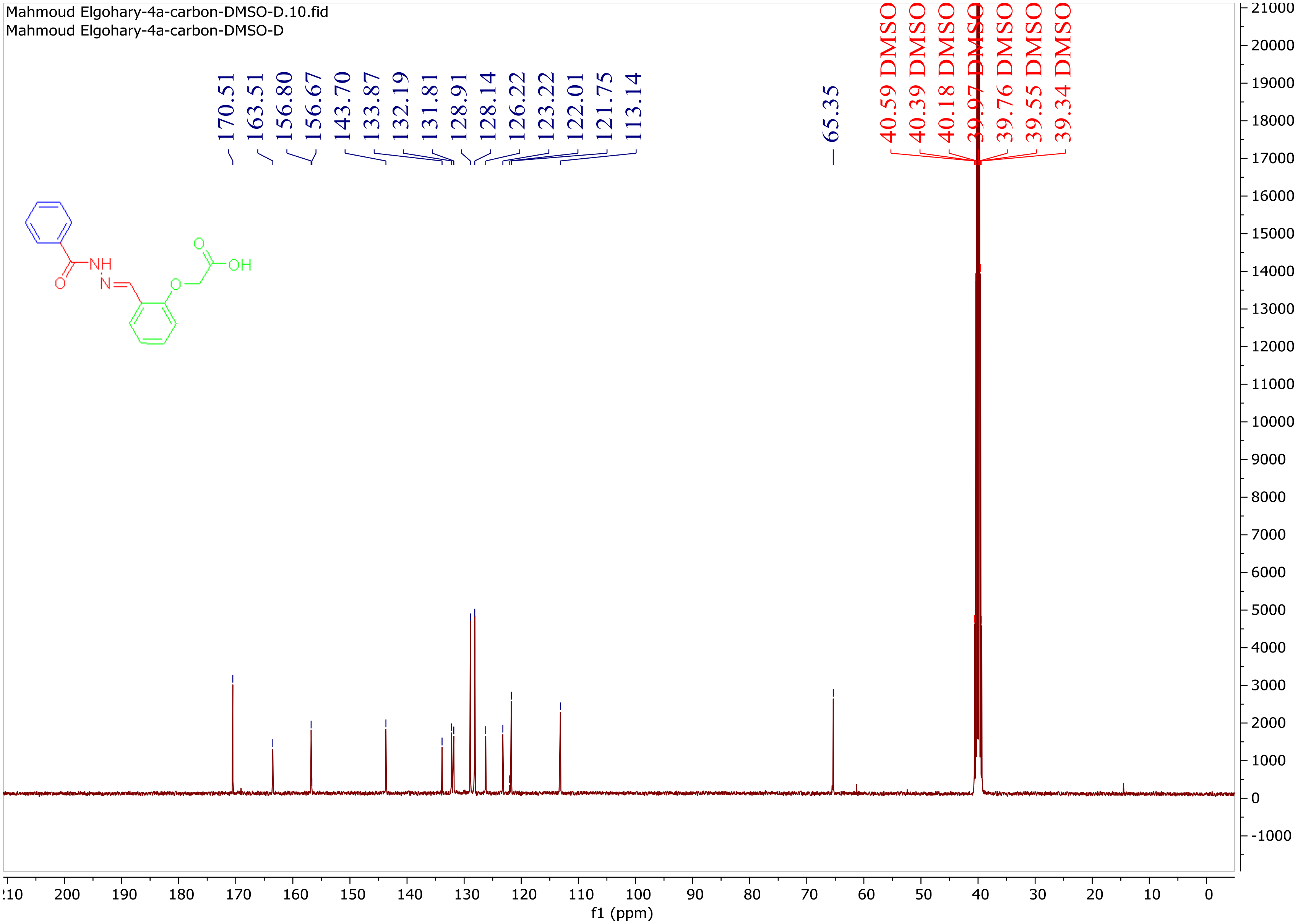

Figure S7. ^{13}C NMR of compound **5a**

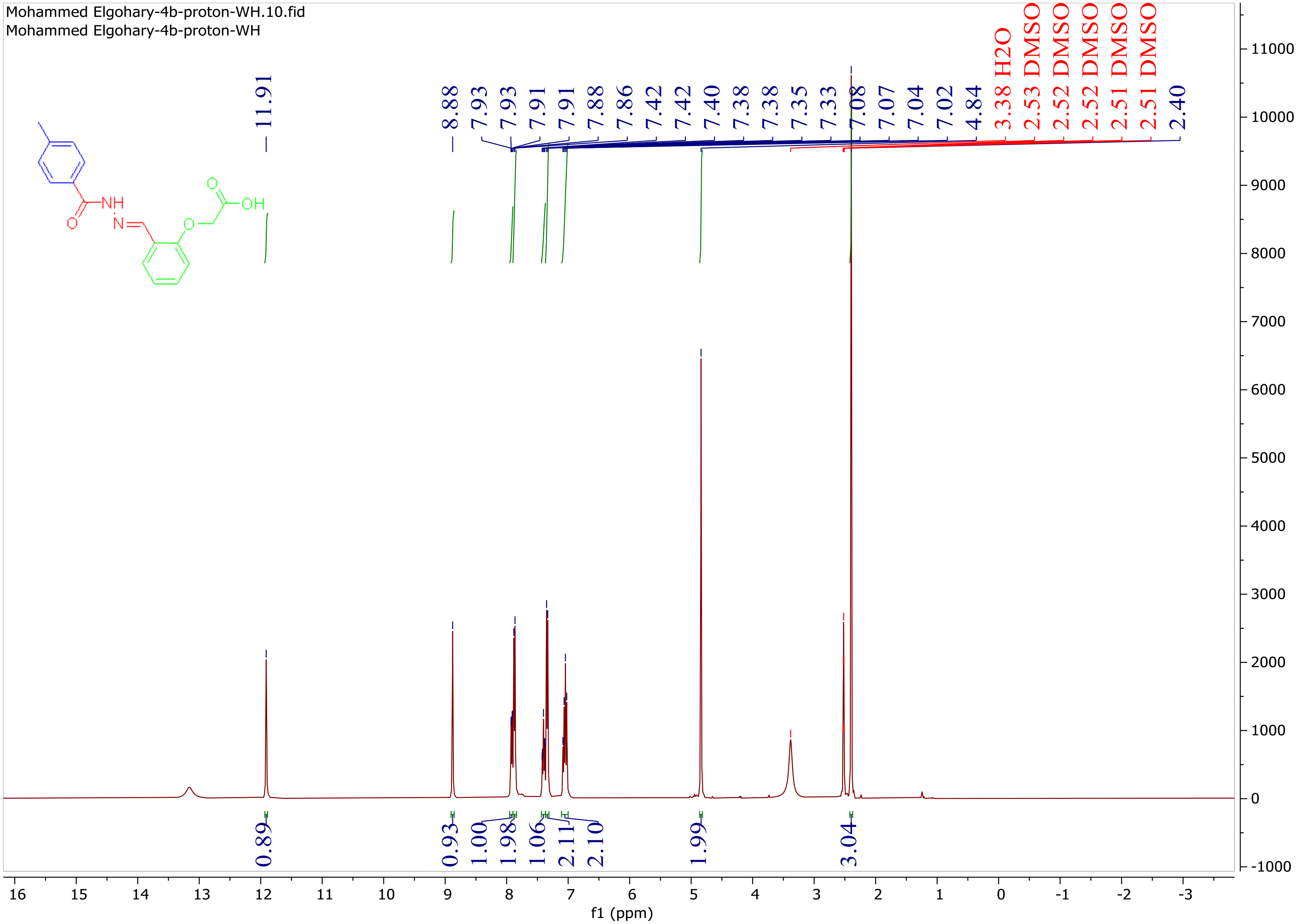

Figure S6. ^{1}H NMR of compound **5b**

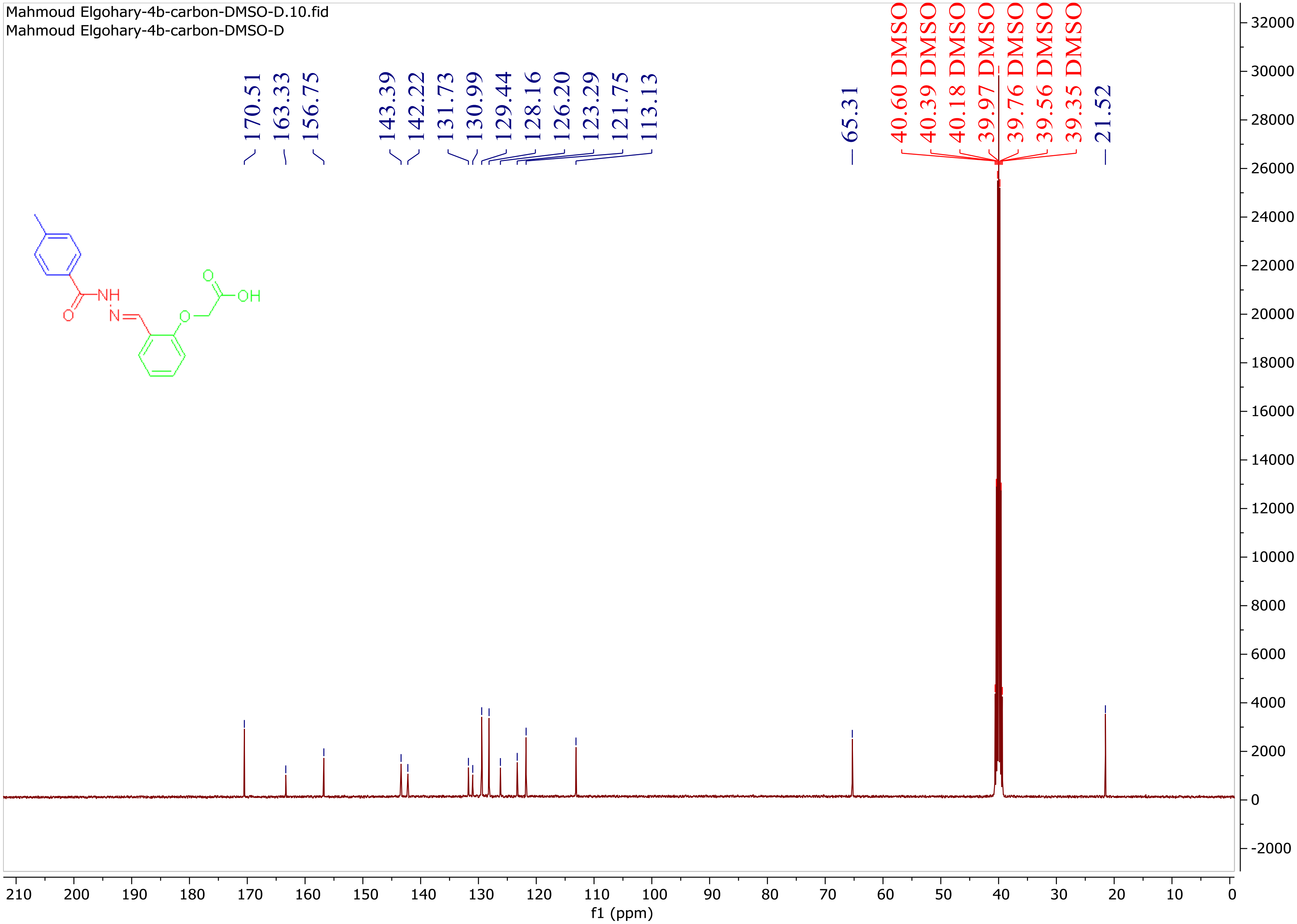

Figure S7. ^{13}C NMR of compound **5b**

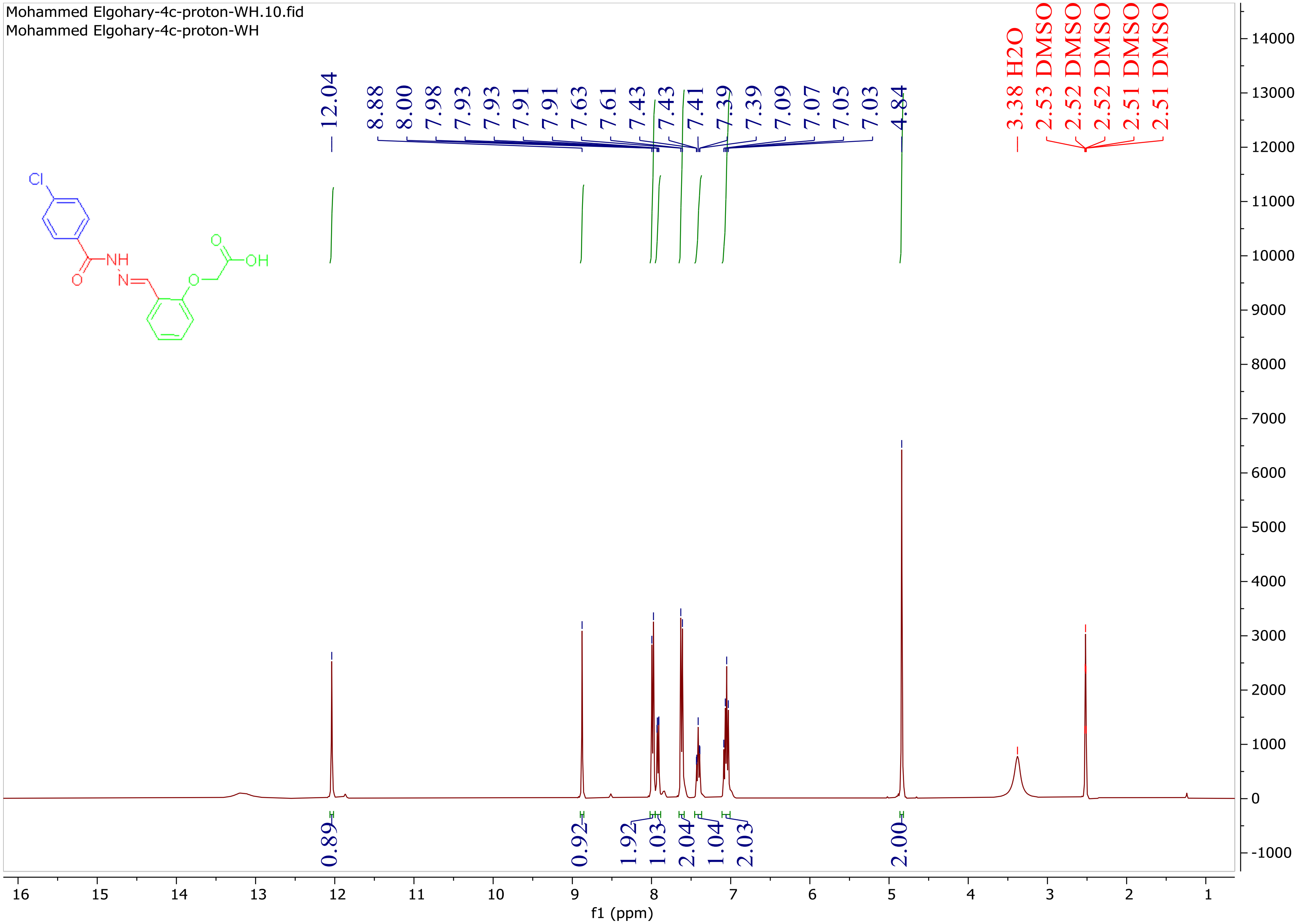

Figure S8. ^{1}H NMR of compound **5c**

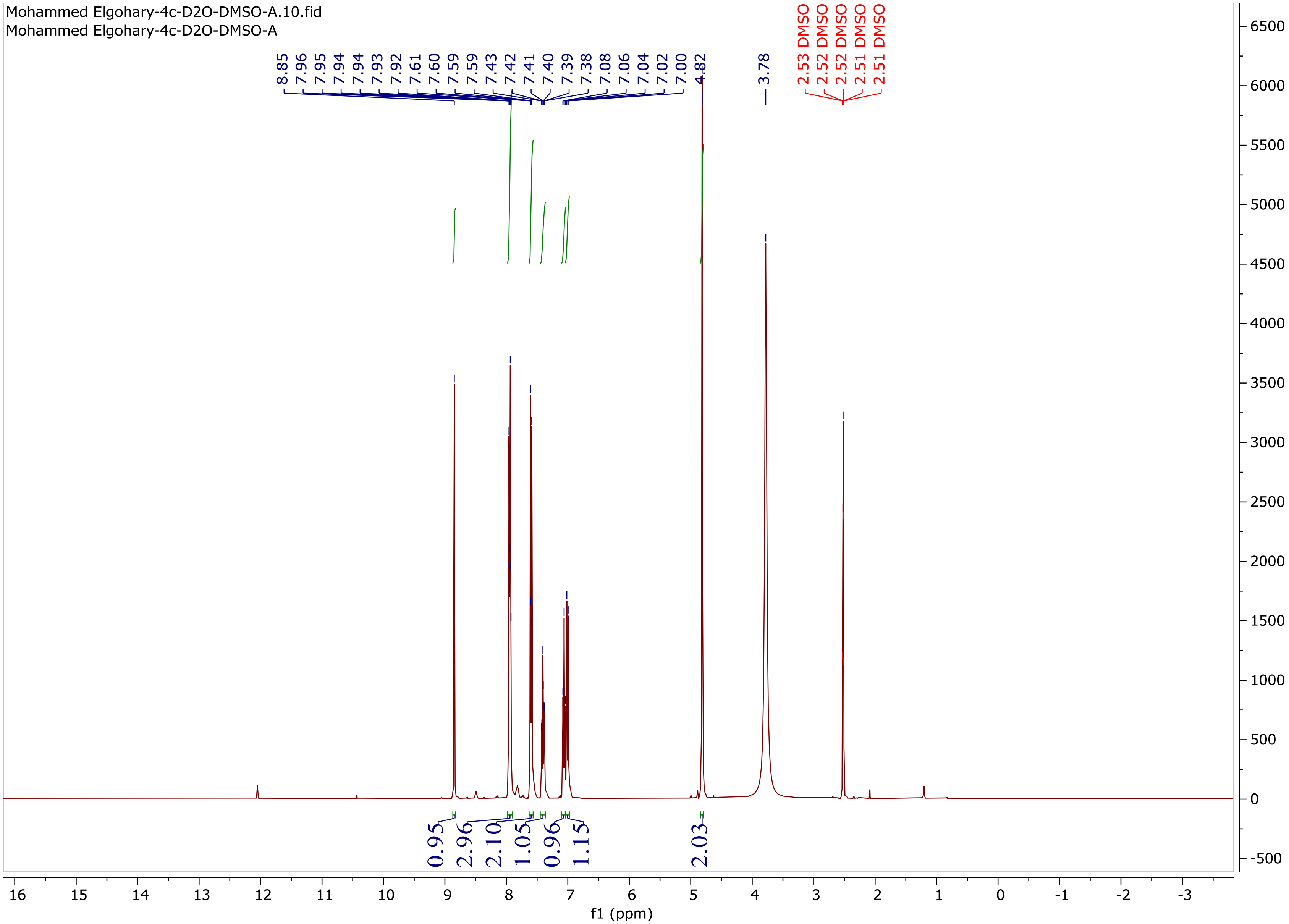

Figure S8. D_2O of compound **5c**

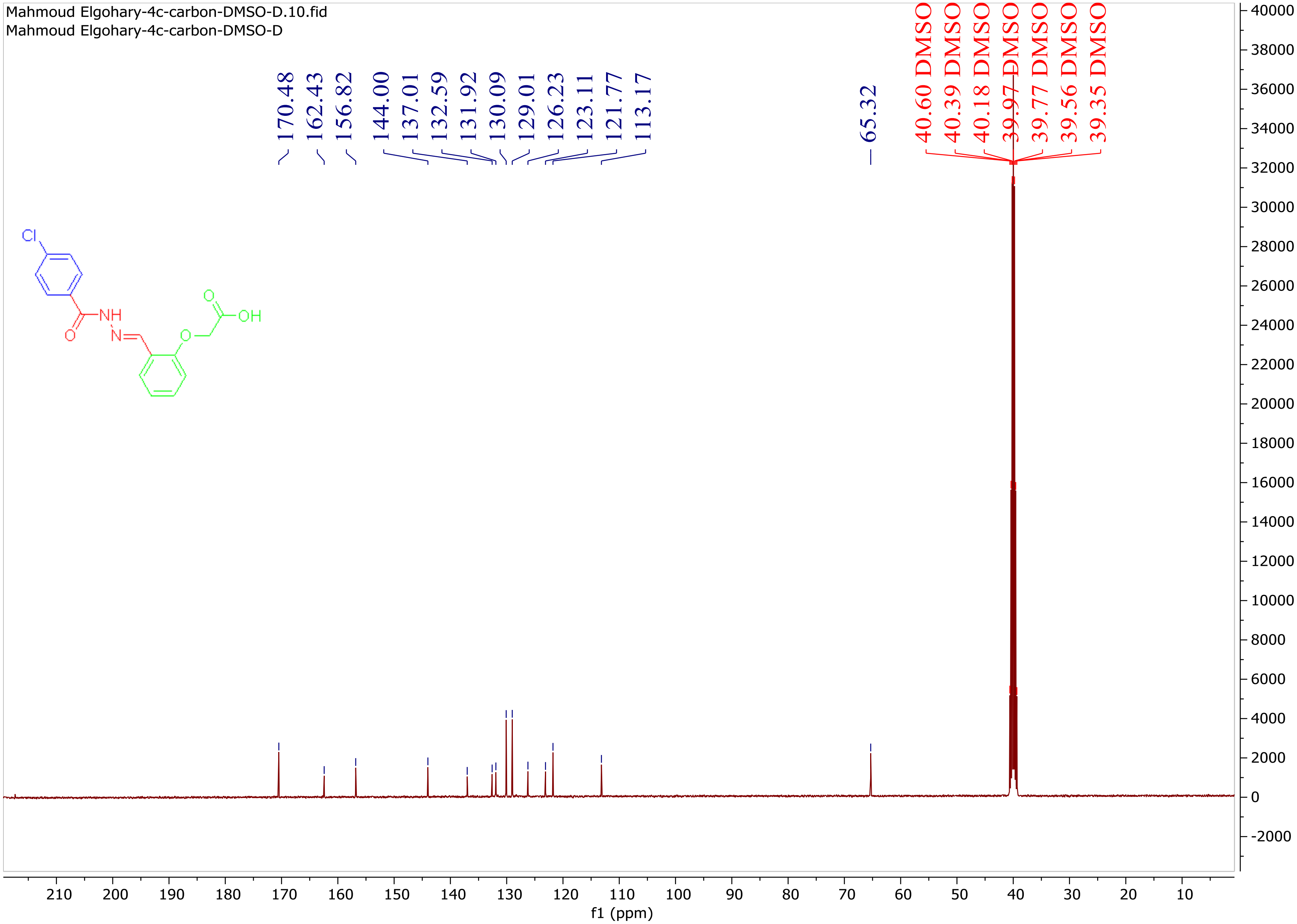

Figure S9. ^{13}C NMR of compound **5c**

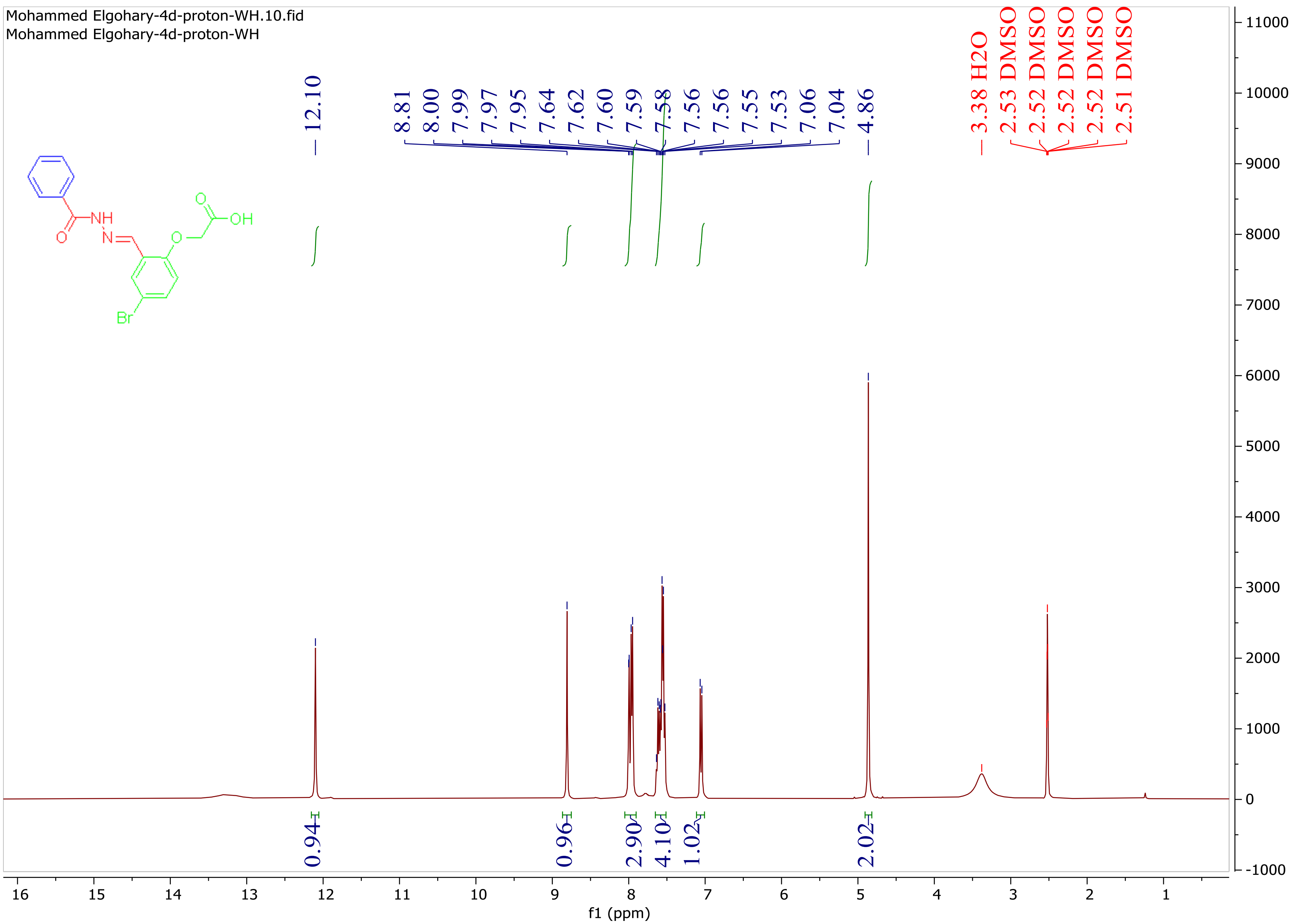

Figure S10. ^{1}H NMR of compound **5d**

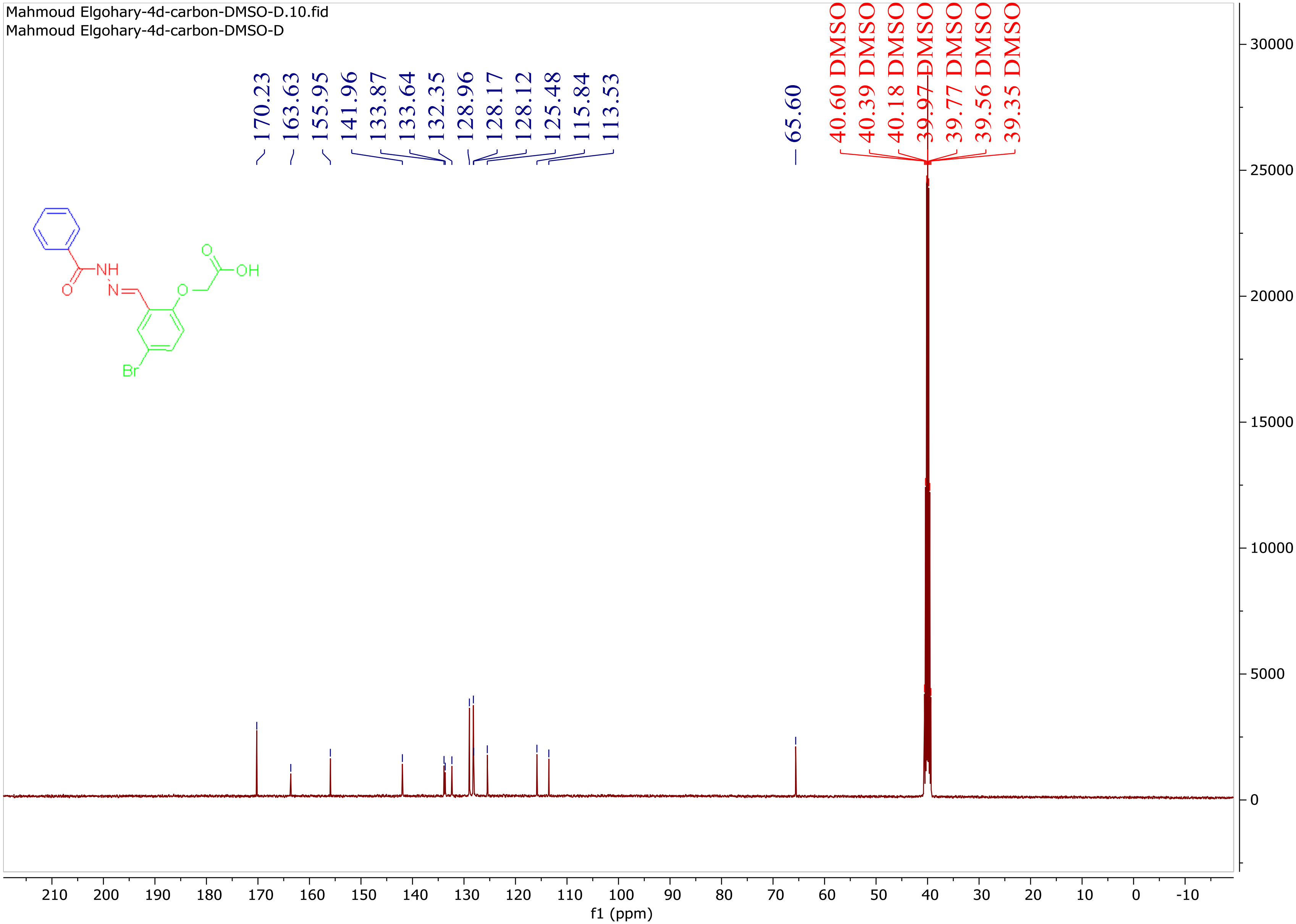

Figure S11. ^{13}C NMR of compound **5d**

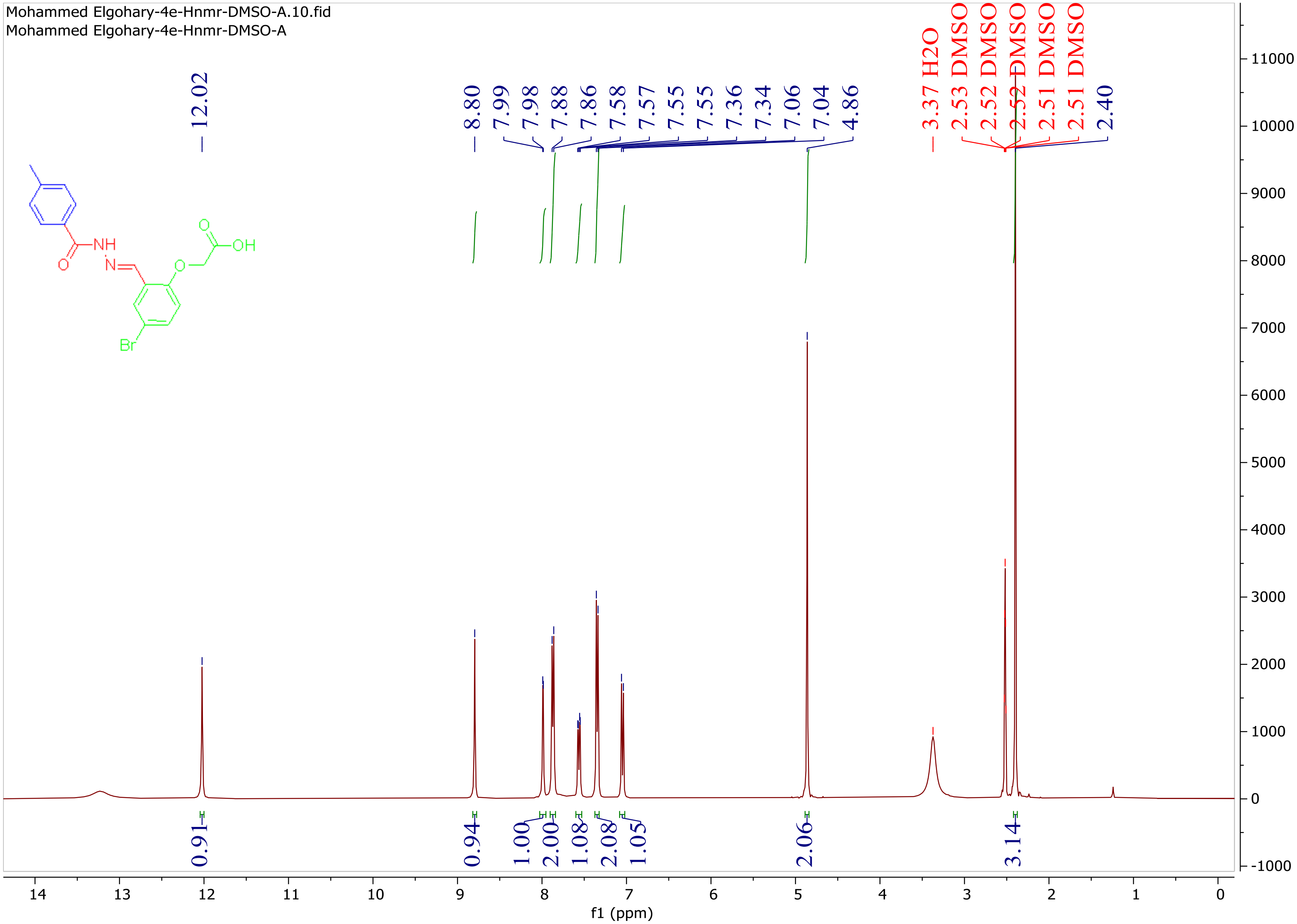

Figure S12. ^{1}H NMR of compound **5e**

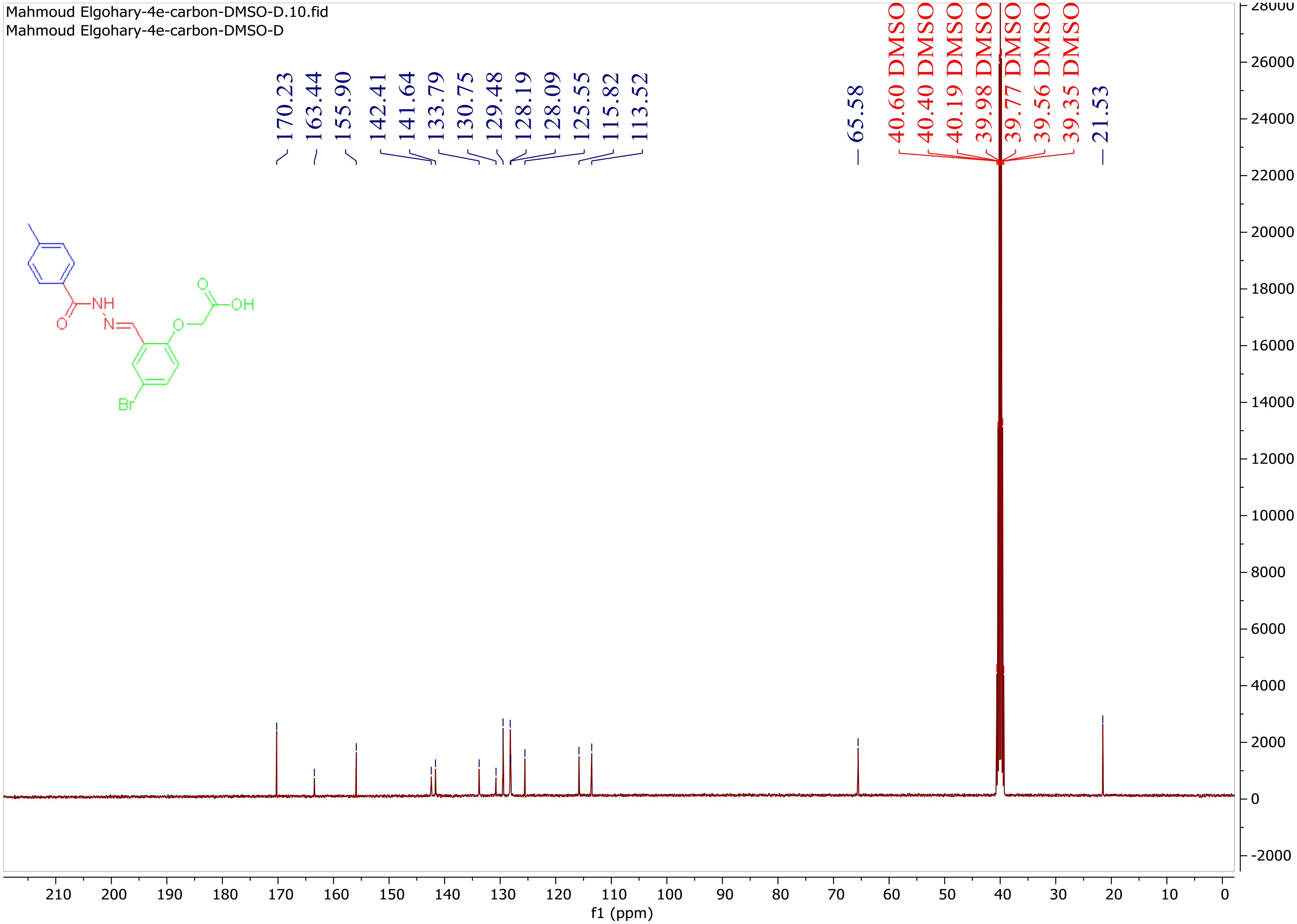

Figure S13. ^{13}C NMR of compound **5e**

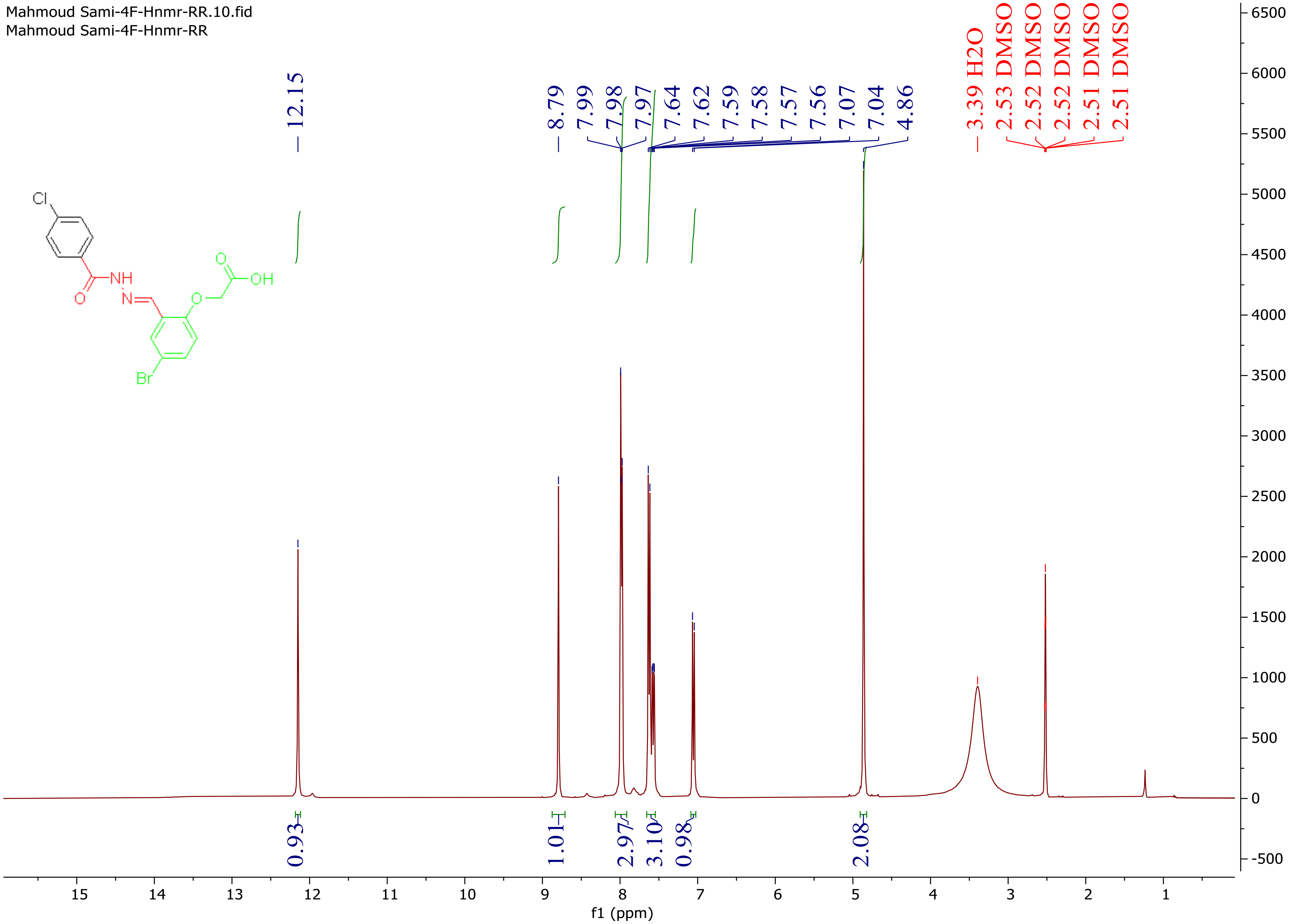

Figure S12. ^{1}H NMR of compound **5f**

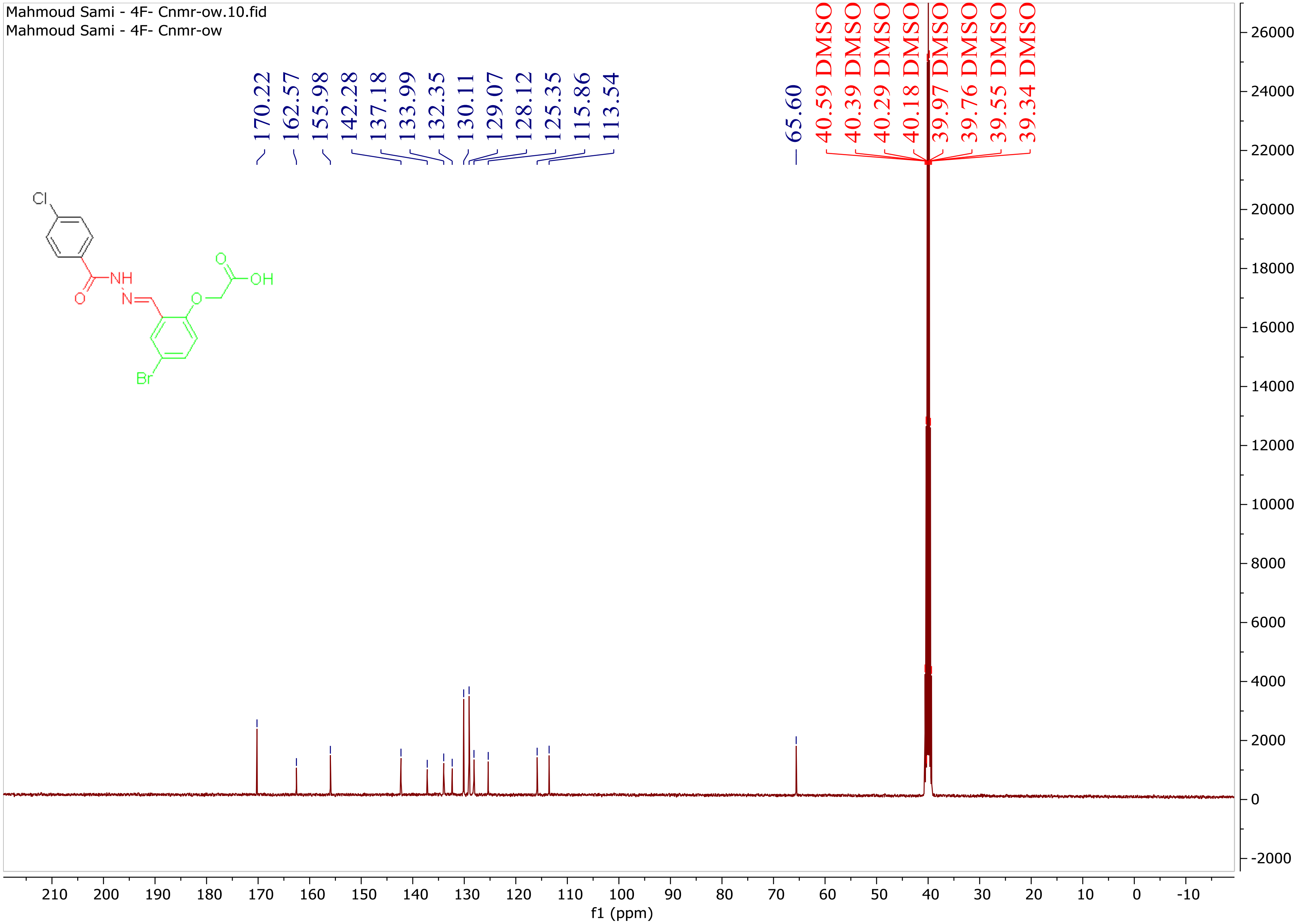

Figure S13. ^{13}C NMR of compound **5f**

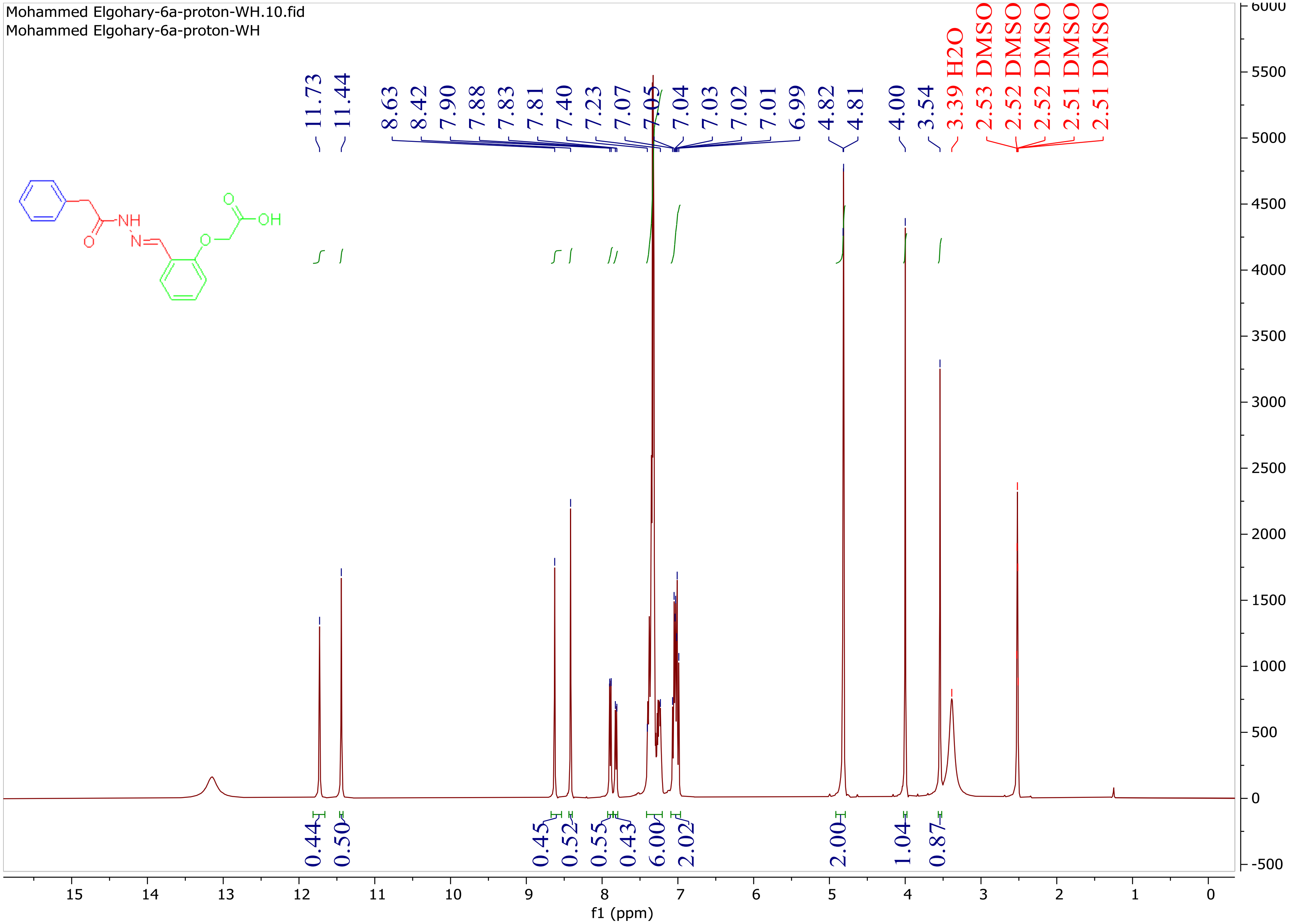

Figure S14. ^{1}H NMR of compound **7a**

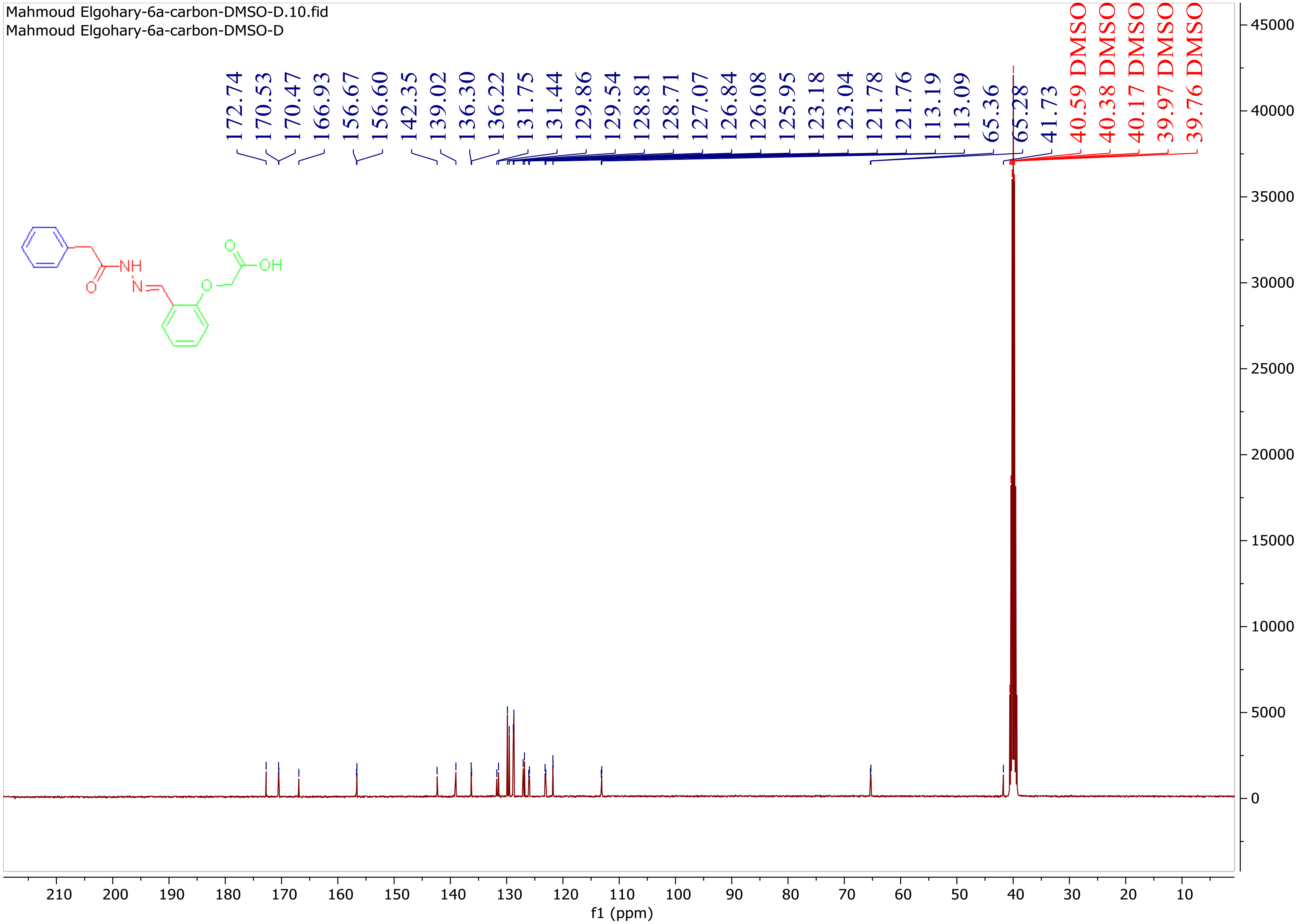

Figure S15. ^{13}C NMR of compound **7a**

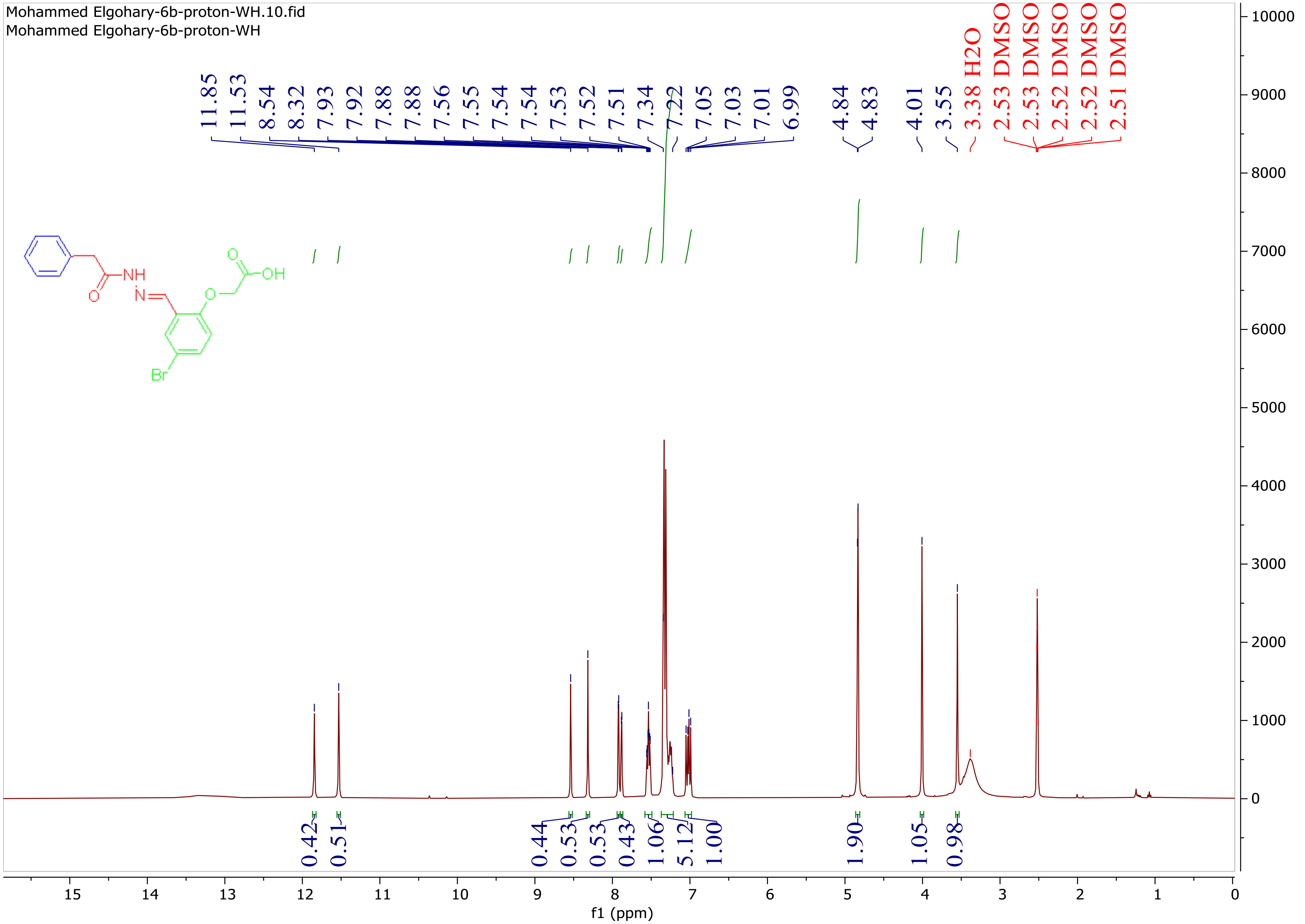

Figure S16. ^{1}H NMR of compound **7b**

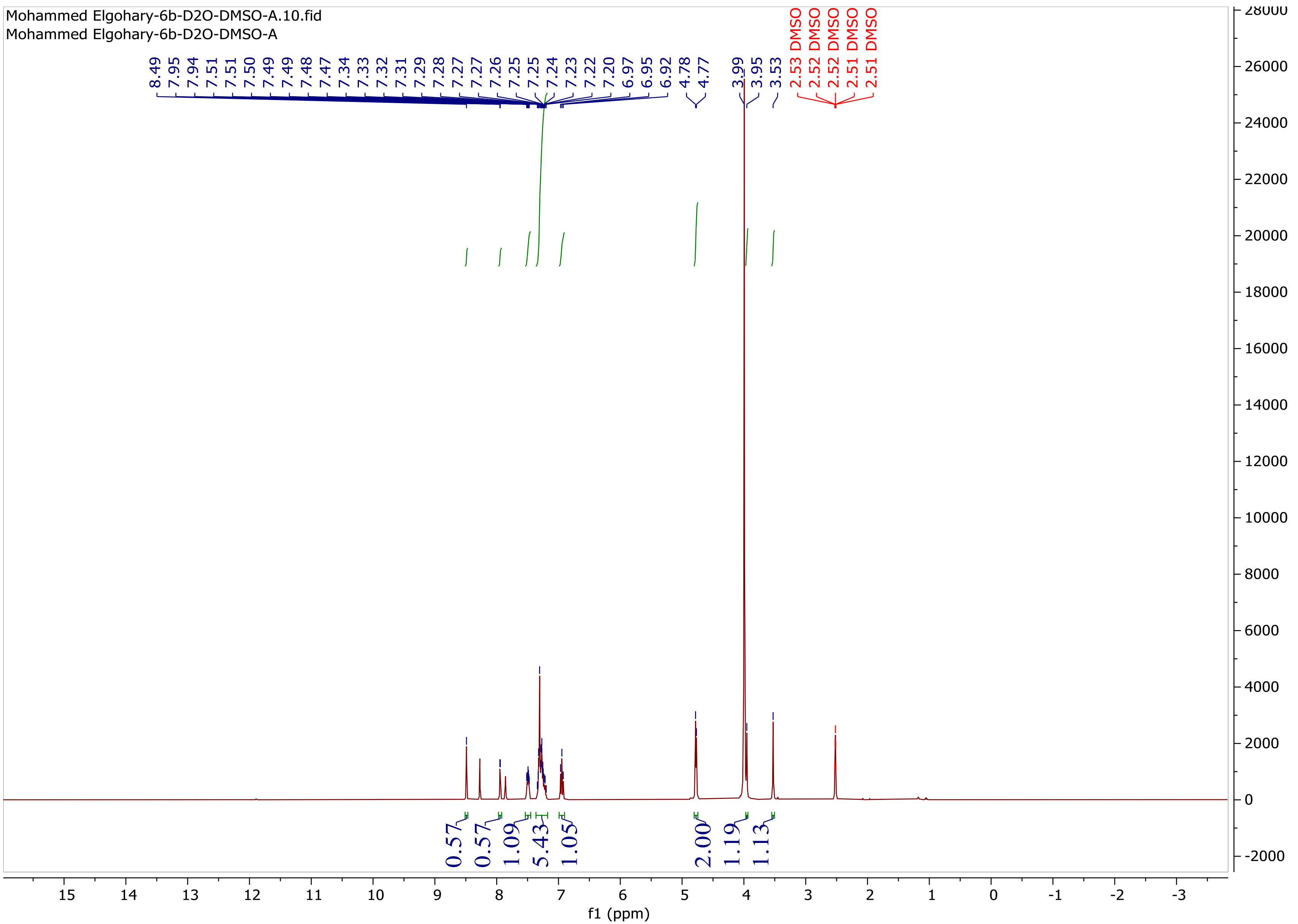

Figure S16. D_2O of compound **7b**

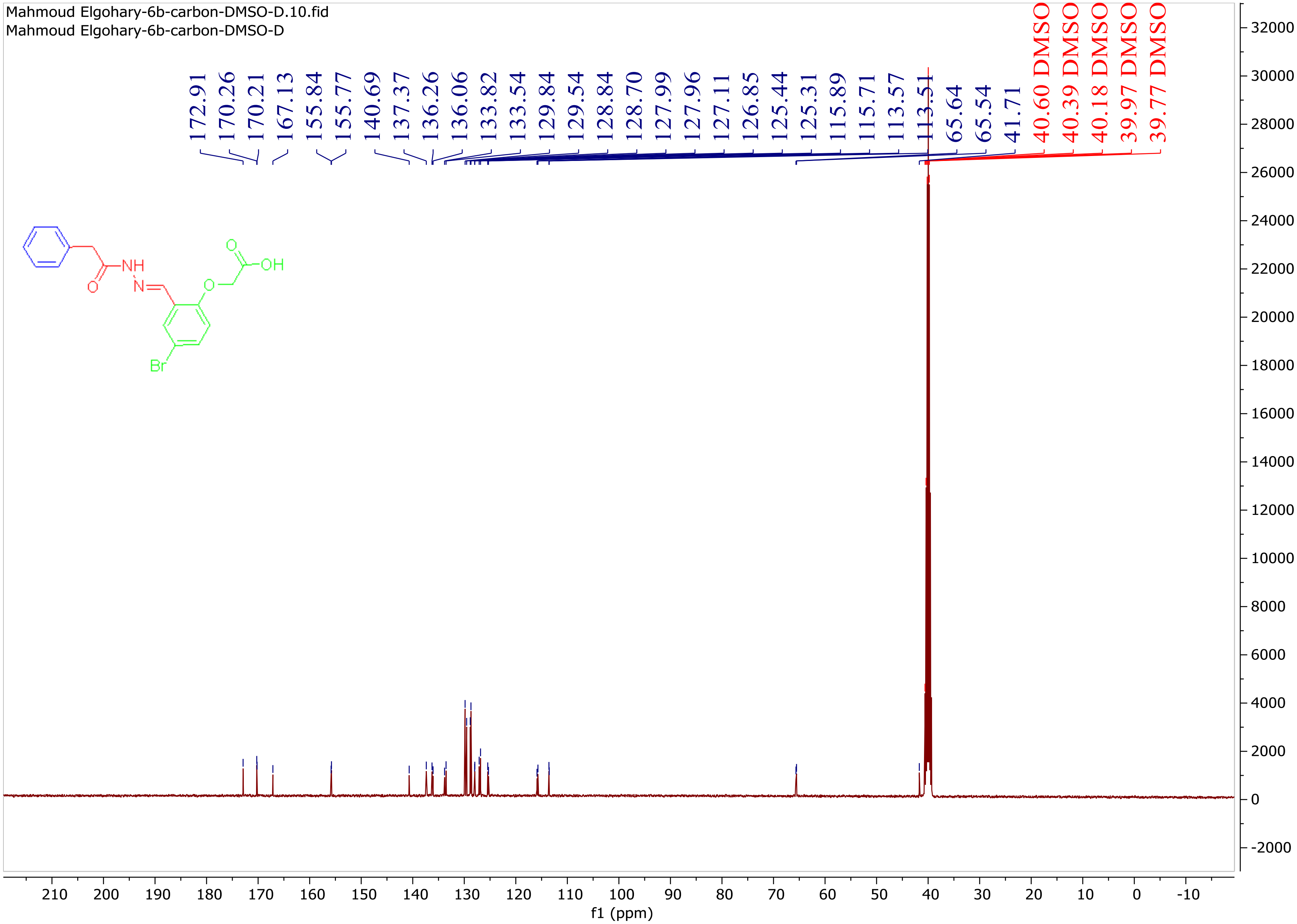

Figure S17. ^{13}C NMR of compound **7b**

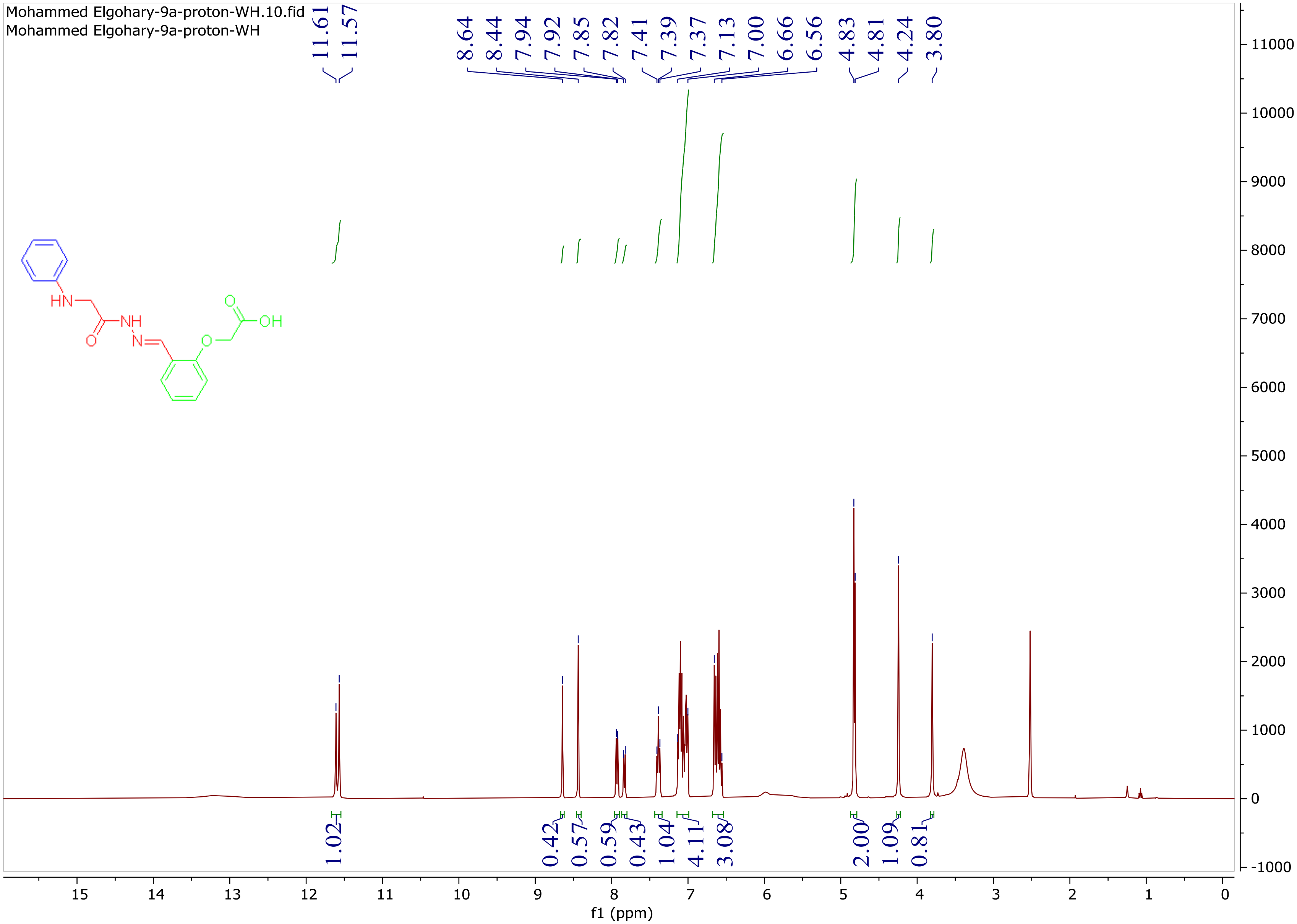

Figure S18. ^{1}H NMR of compound **10a**

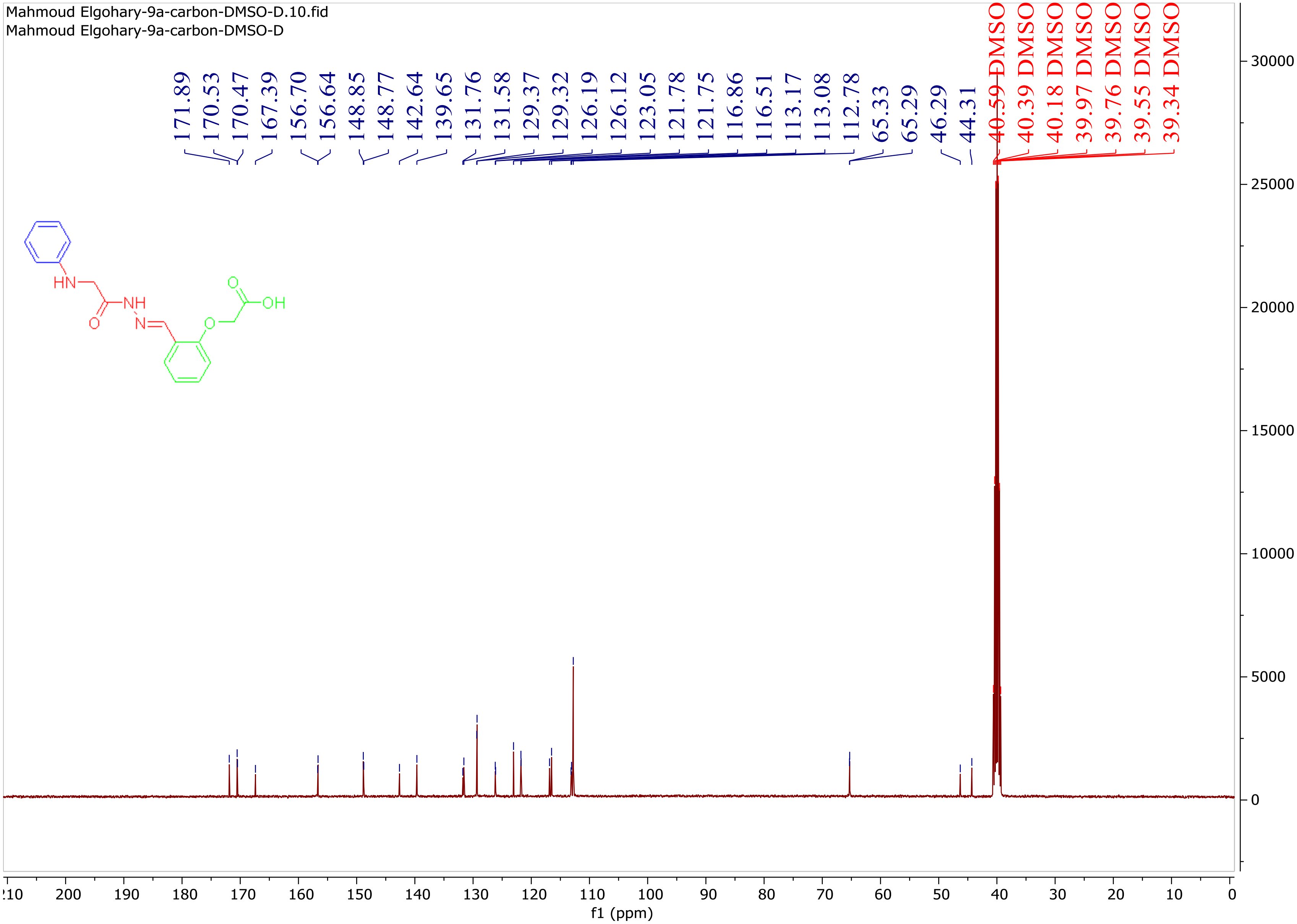

Figure S19. ^{13}C NMR of compound **10a**

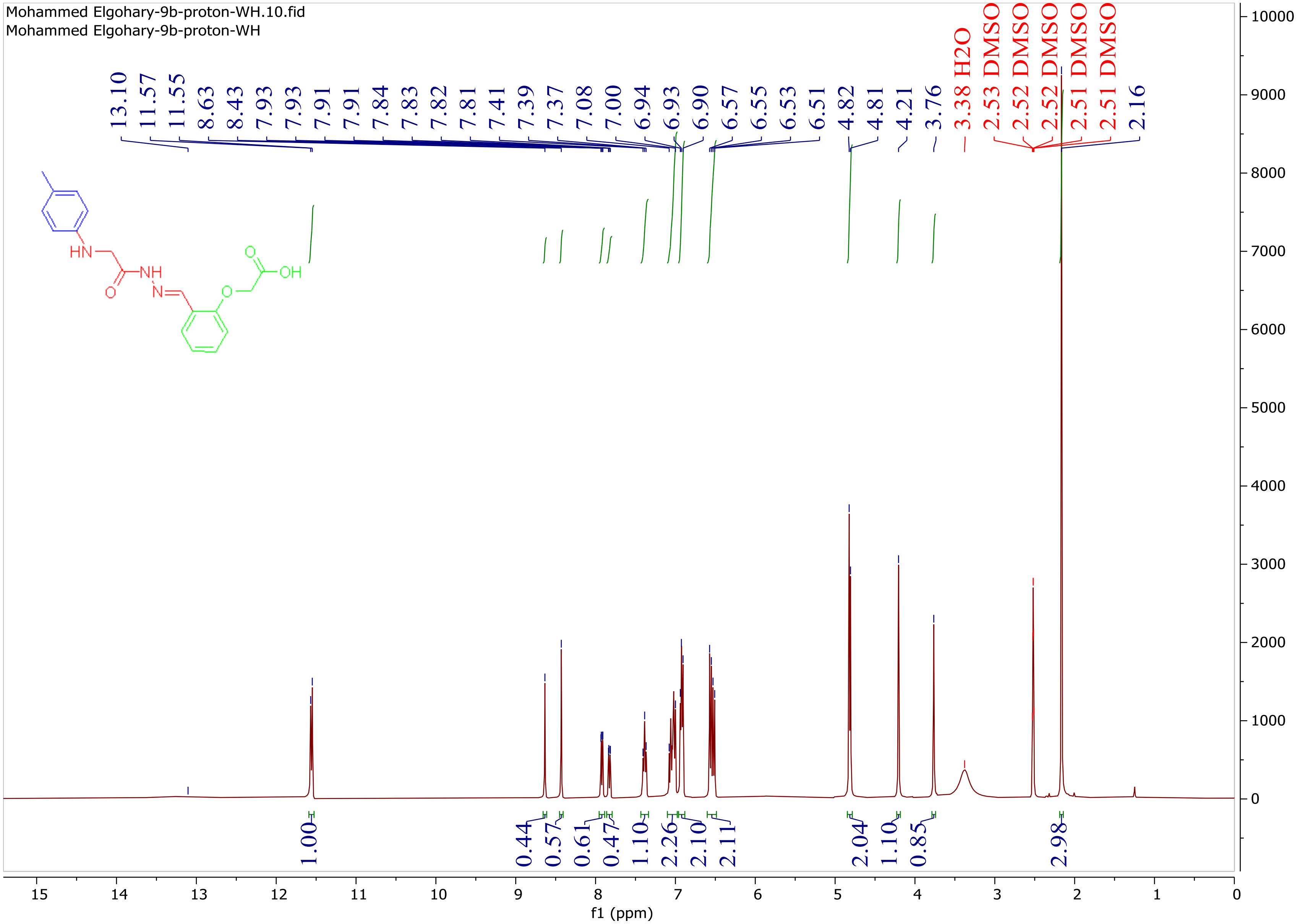

Figure S20. ^{1}H NMR of compound **10b**

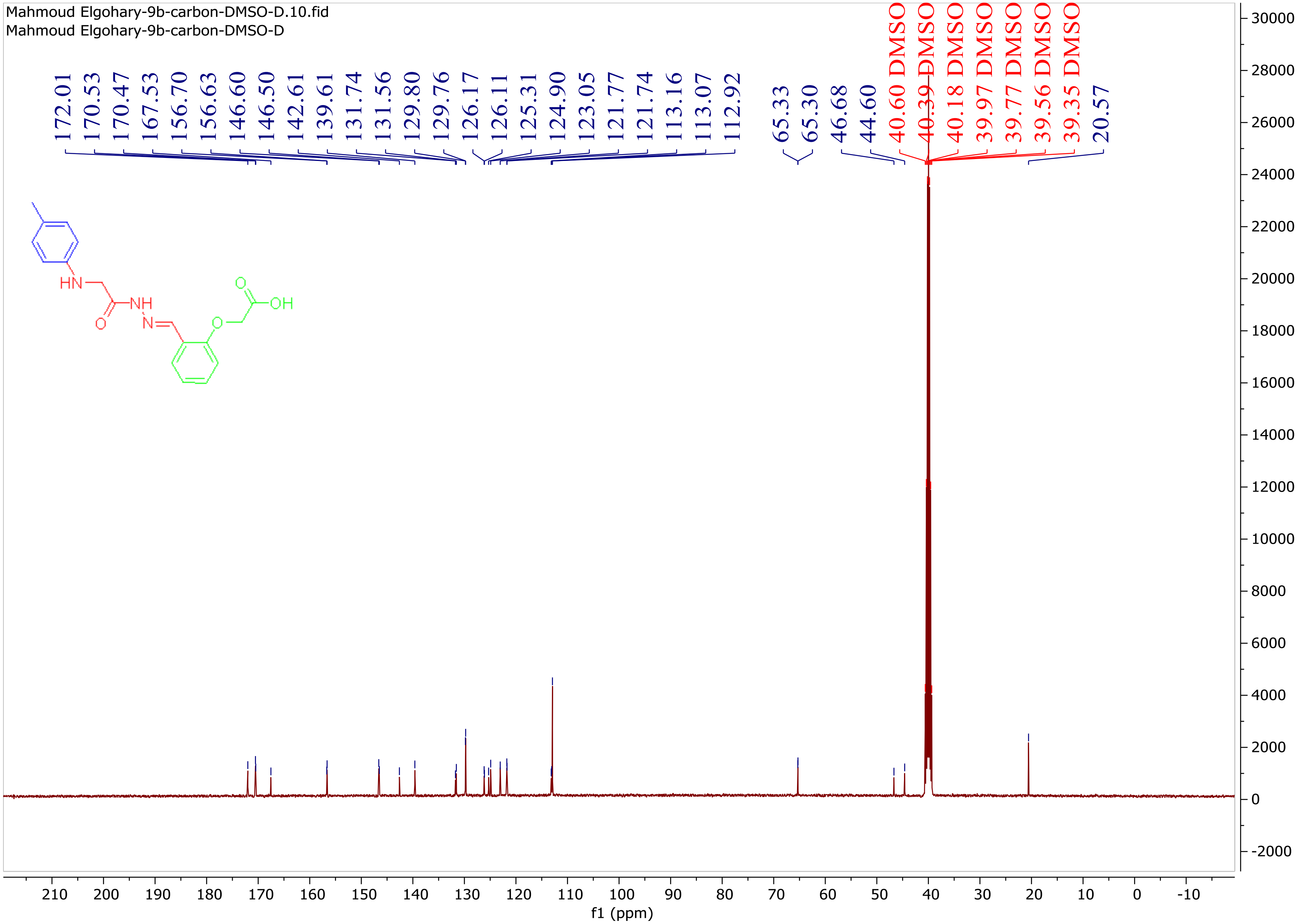

Figure S21. ^{13}C NMR of compound **10b**

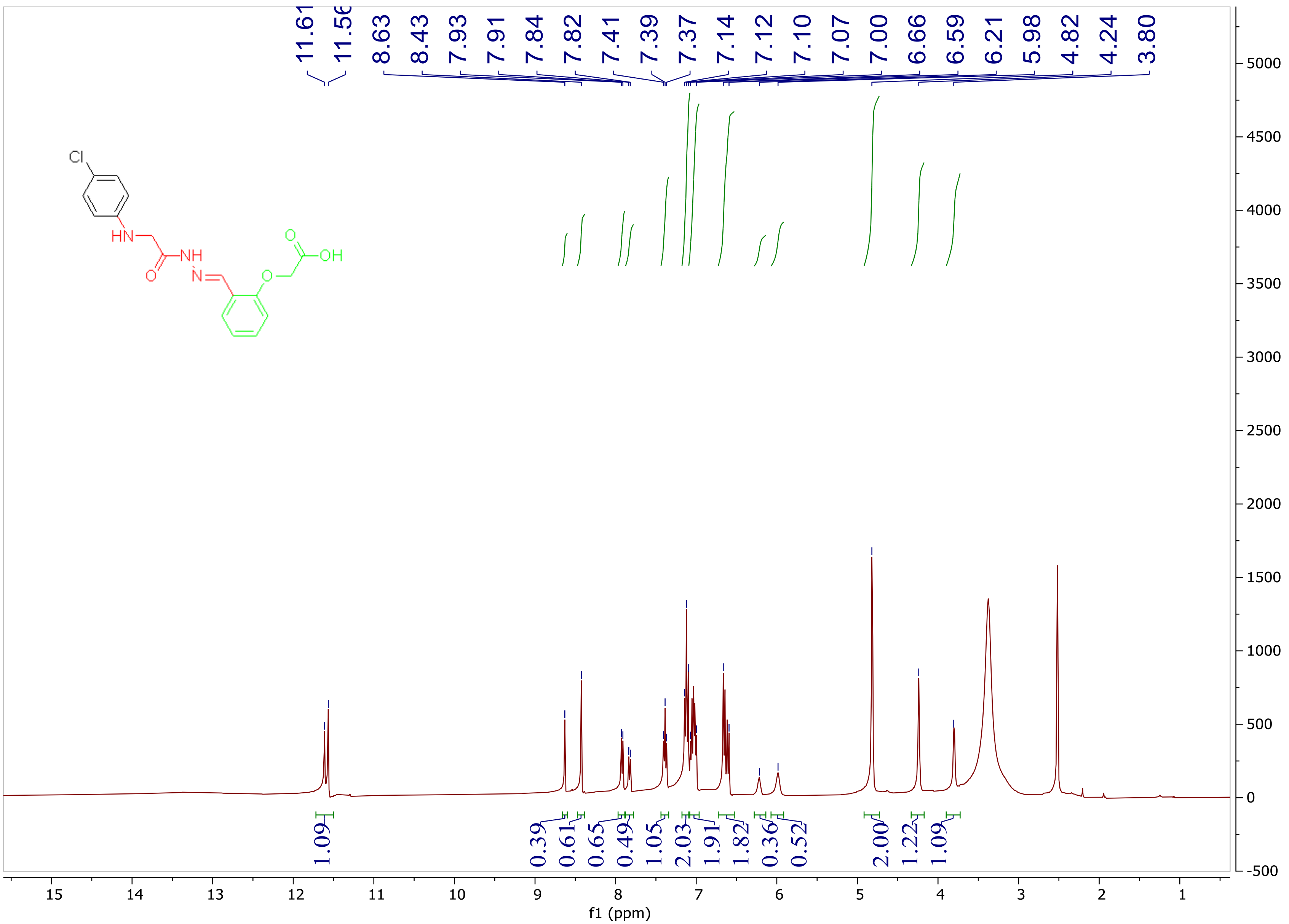

Figure S22. ^{1}H NMR of compound **10c**

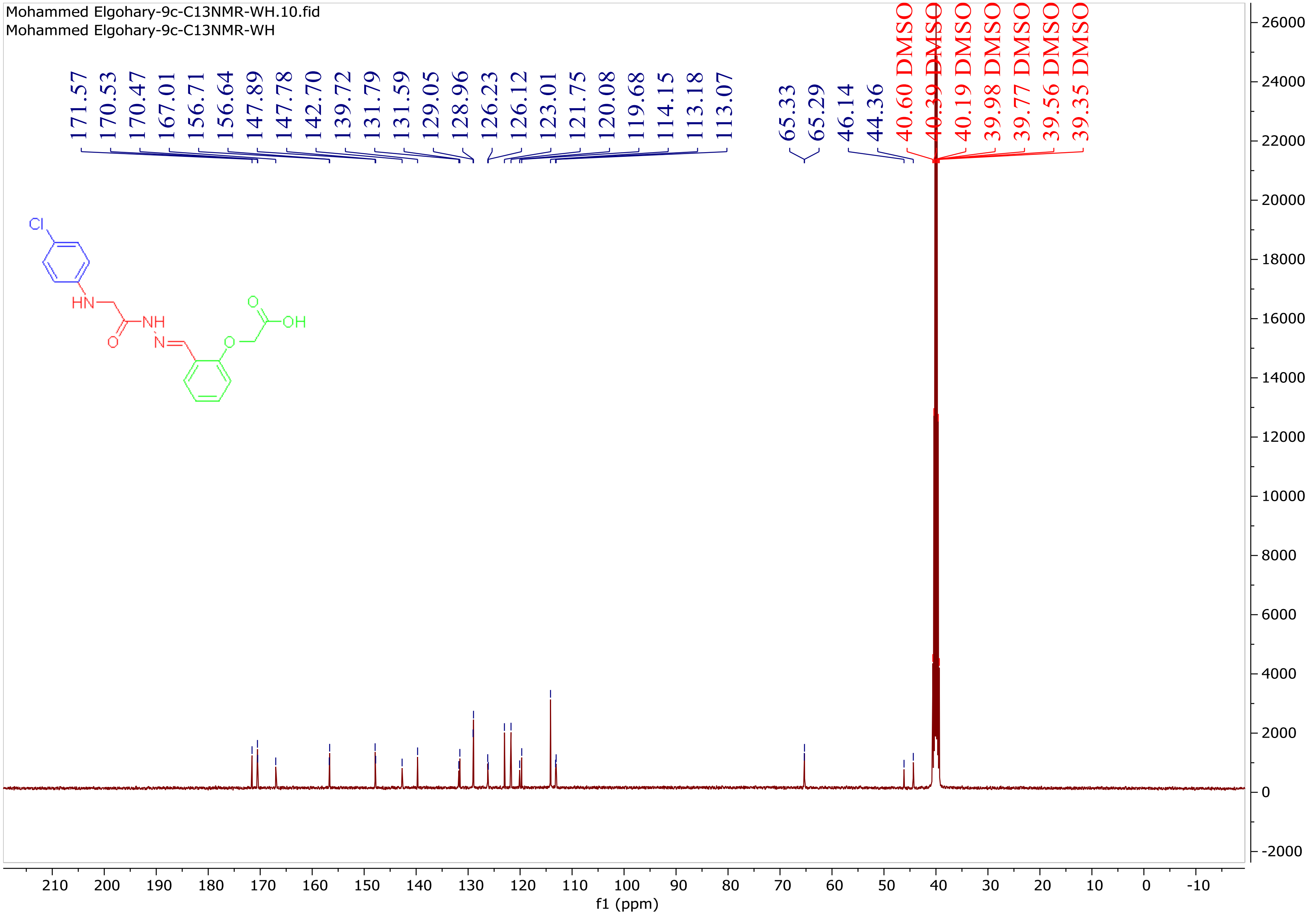

Figure S23. ^{13}C NMR of compound **10c**

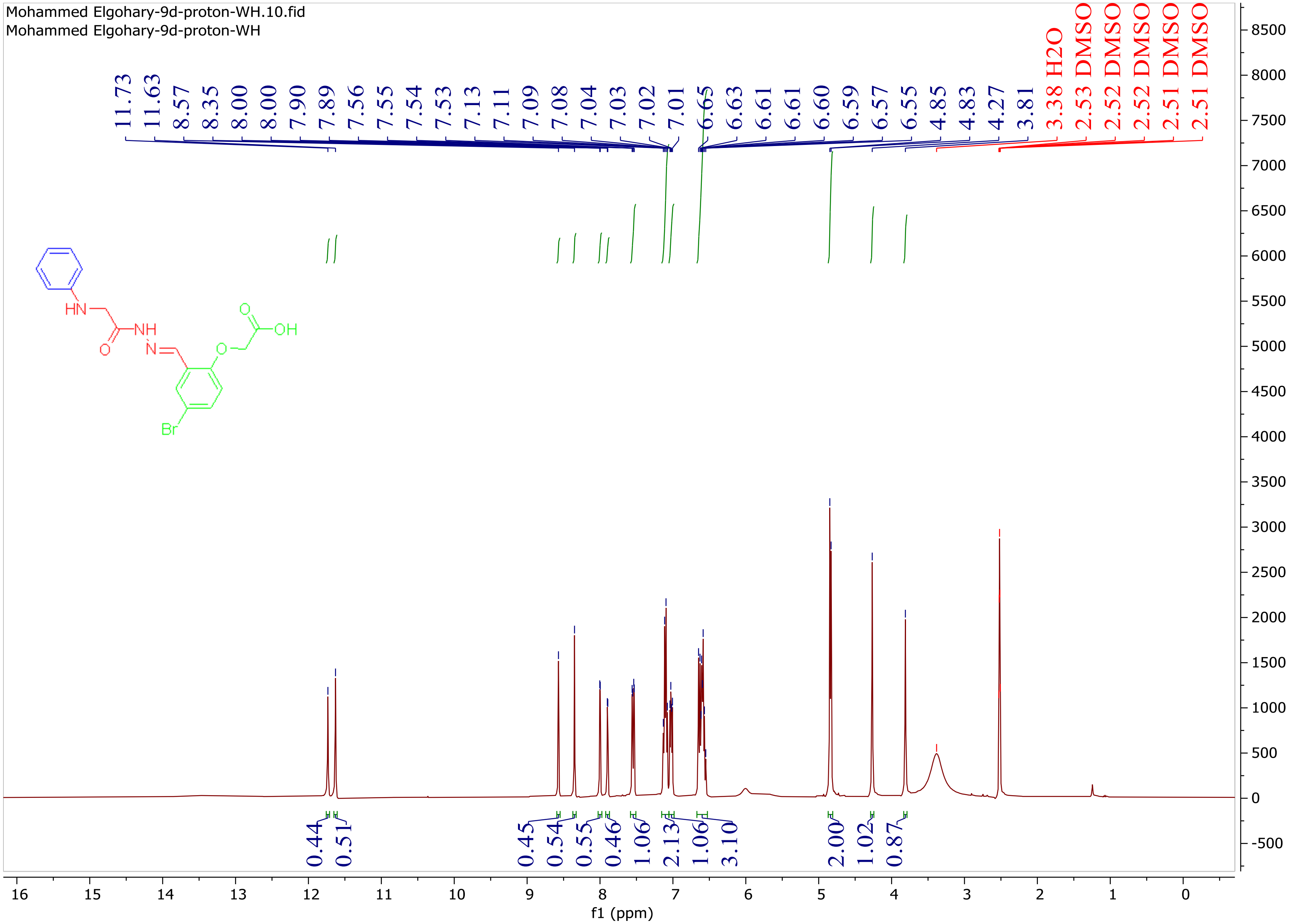

Figure S22. ^{1}H NMR of compound **10d**

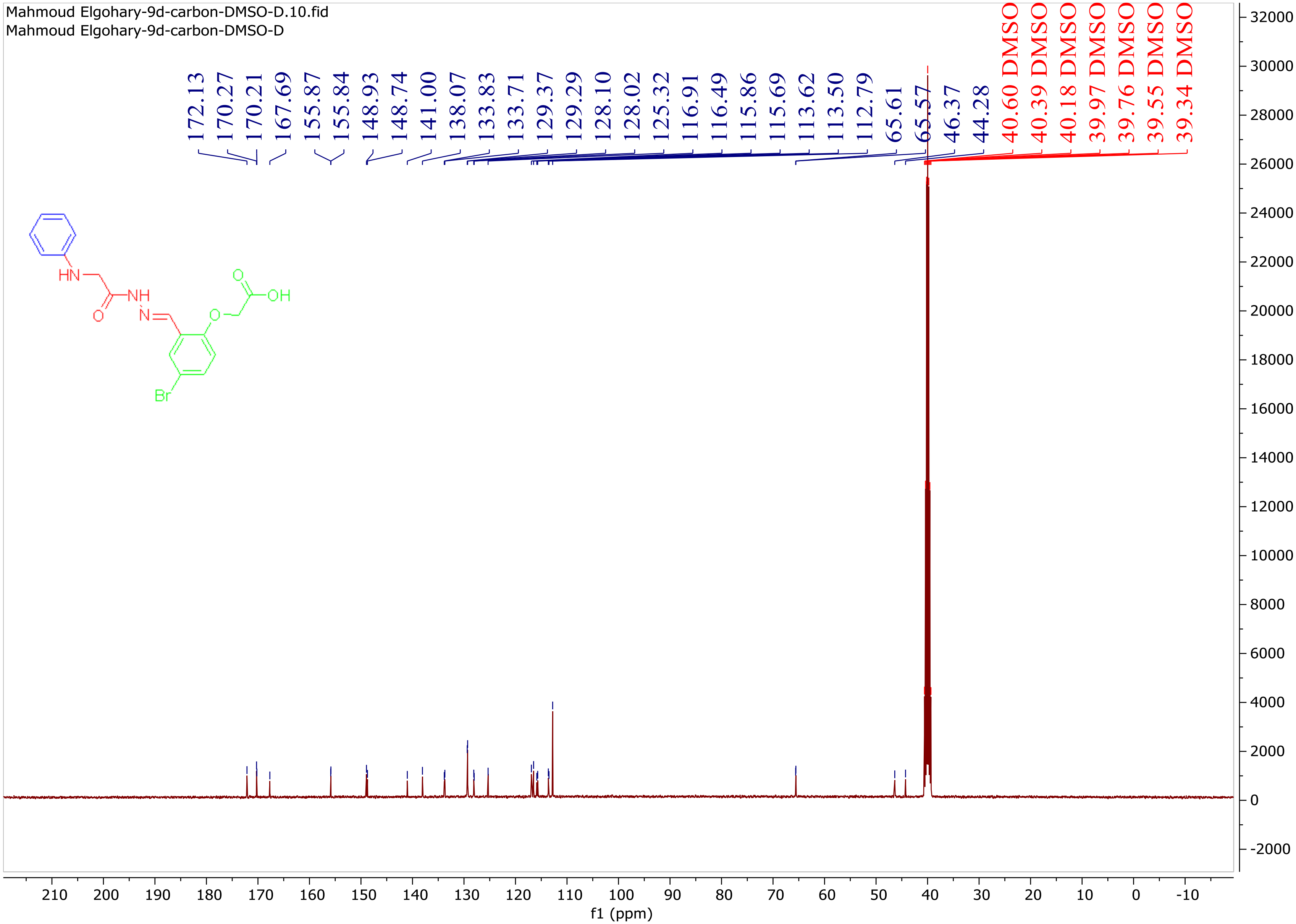

Figure S23. ^{13}C NMR of compound **10d**

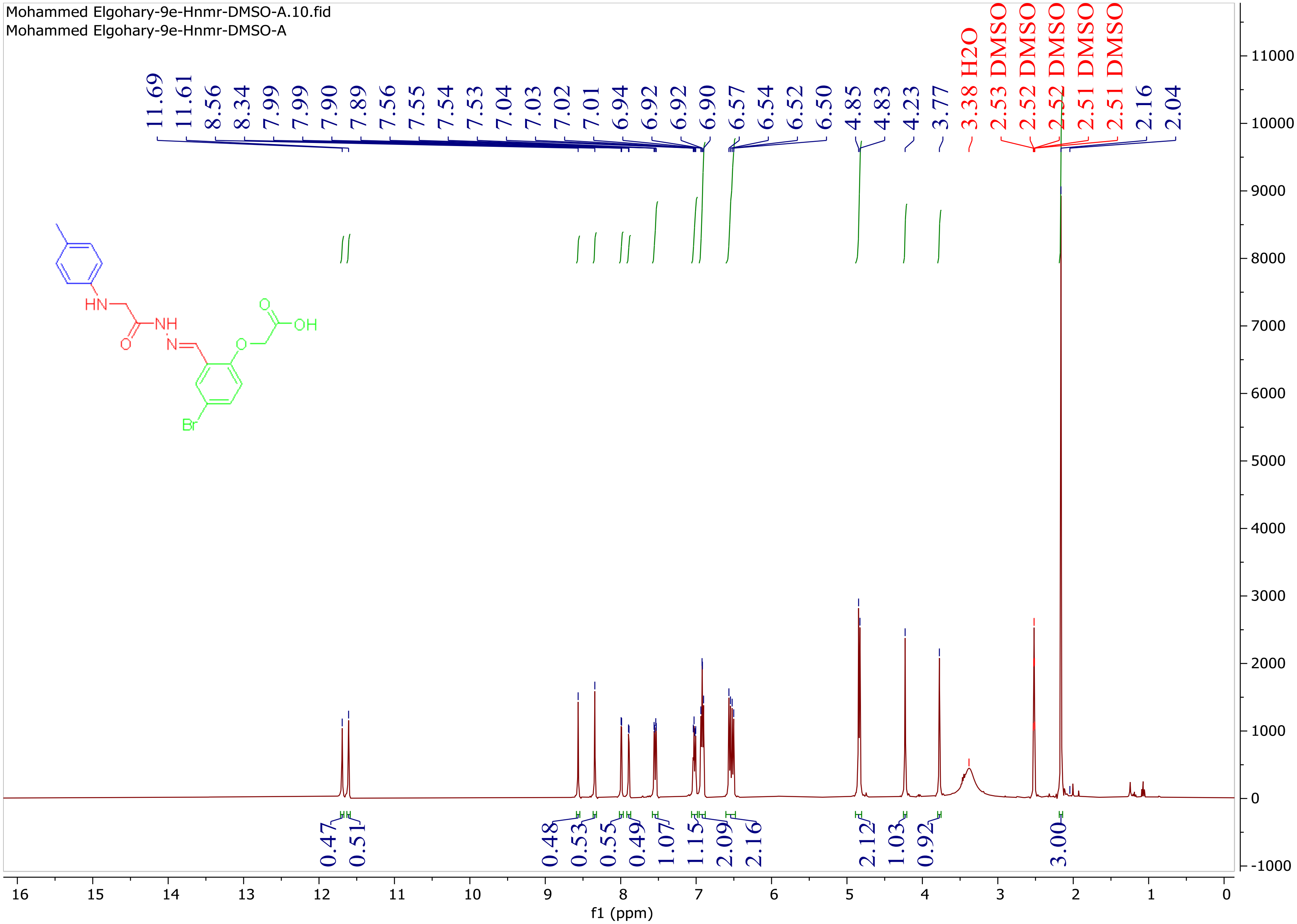

Figure S22. ^{1}H NMR of compound **10e**

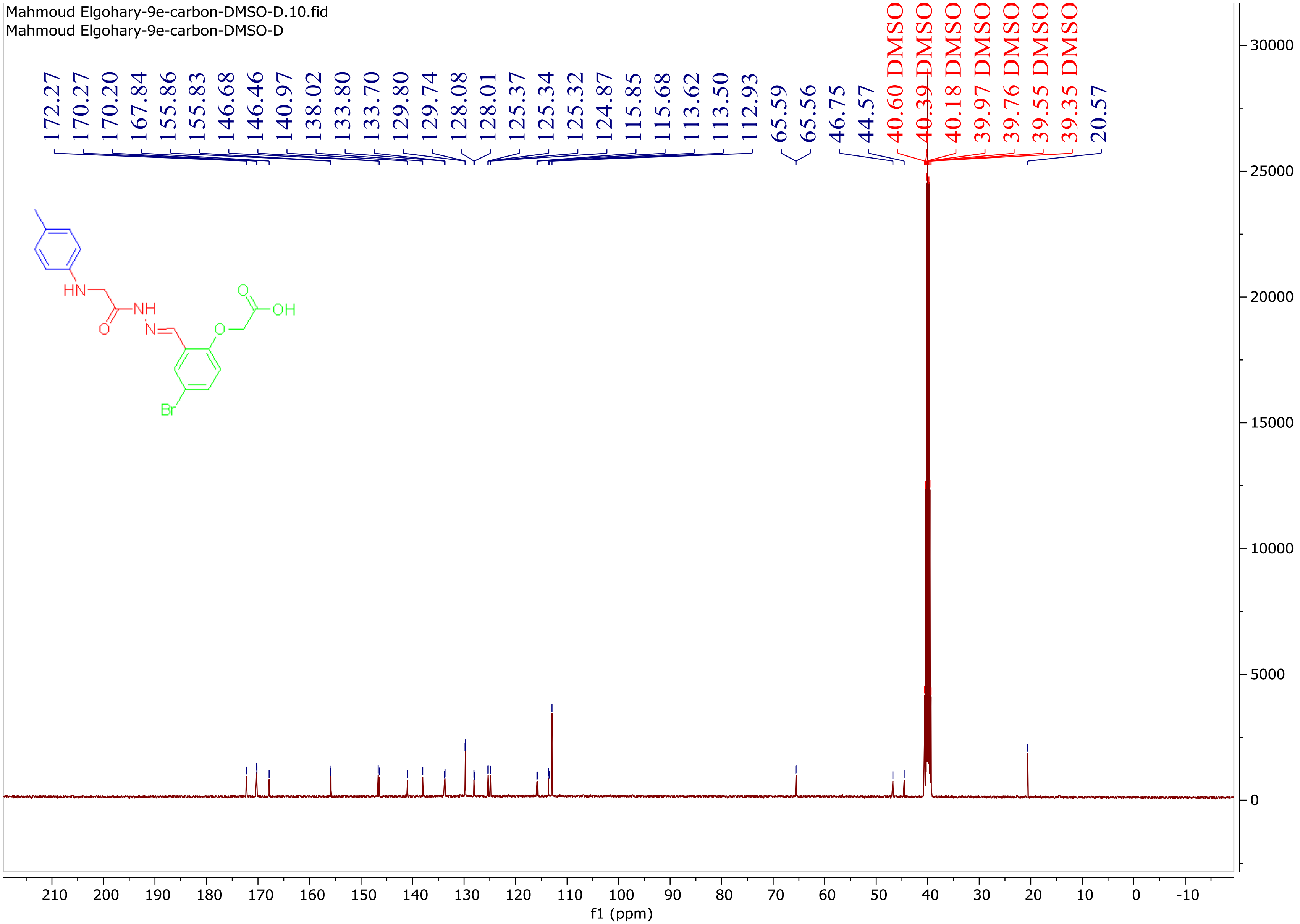

Figure S23. ^{13}C NMR of compound **10e**

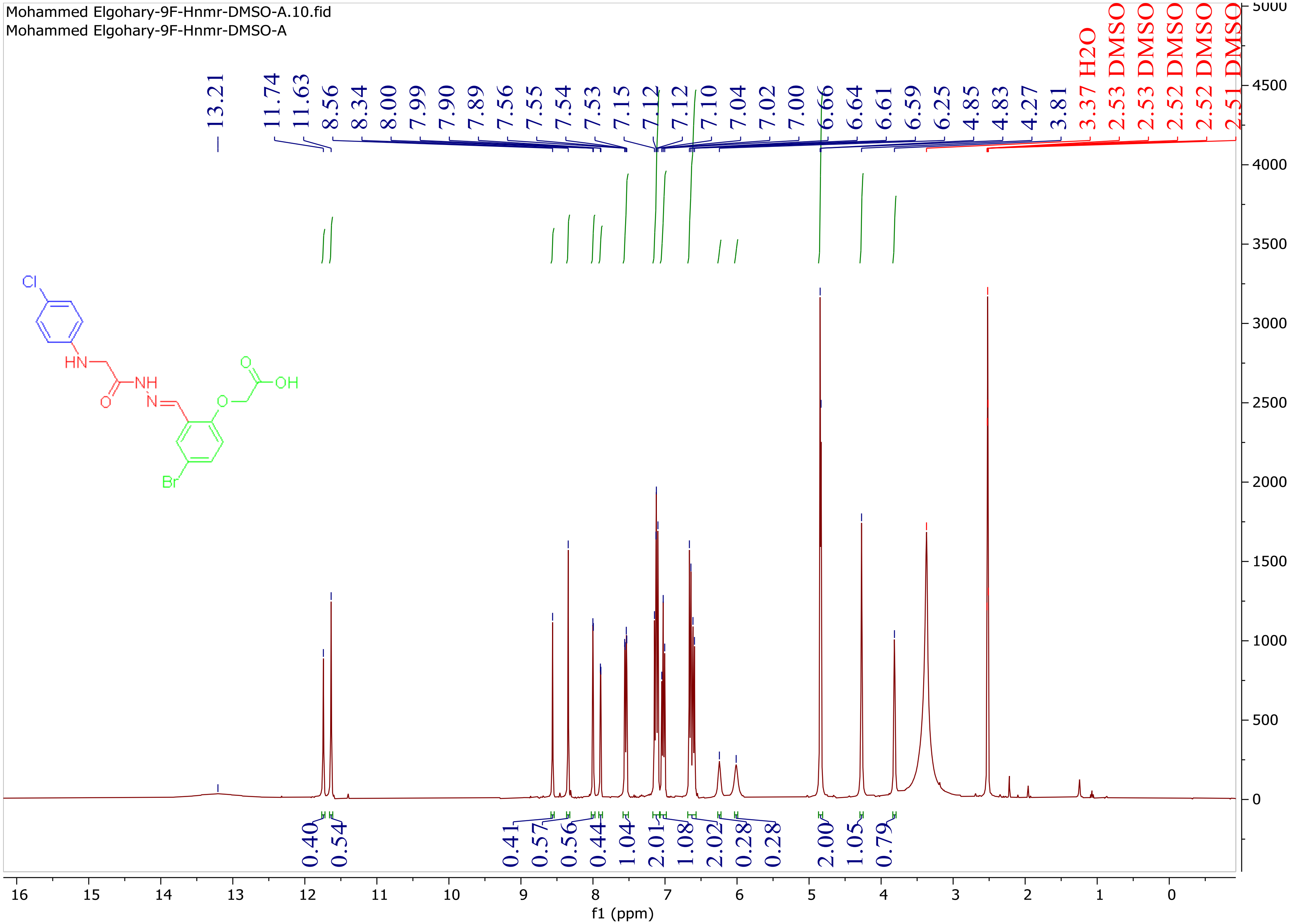

Figure S22. ^{1}H NMR of compound **10f**

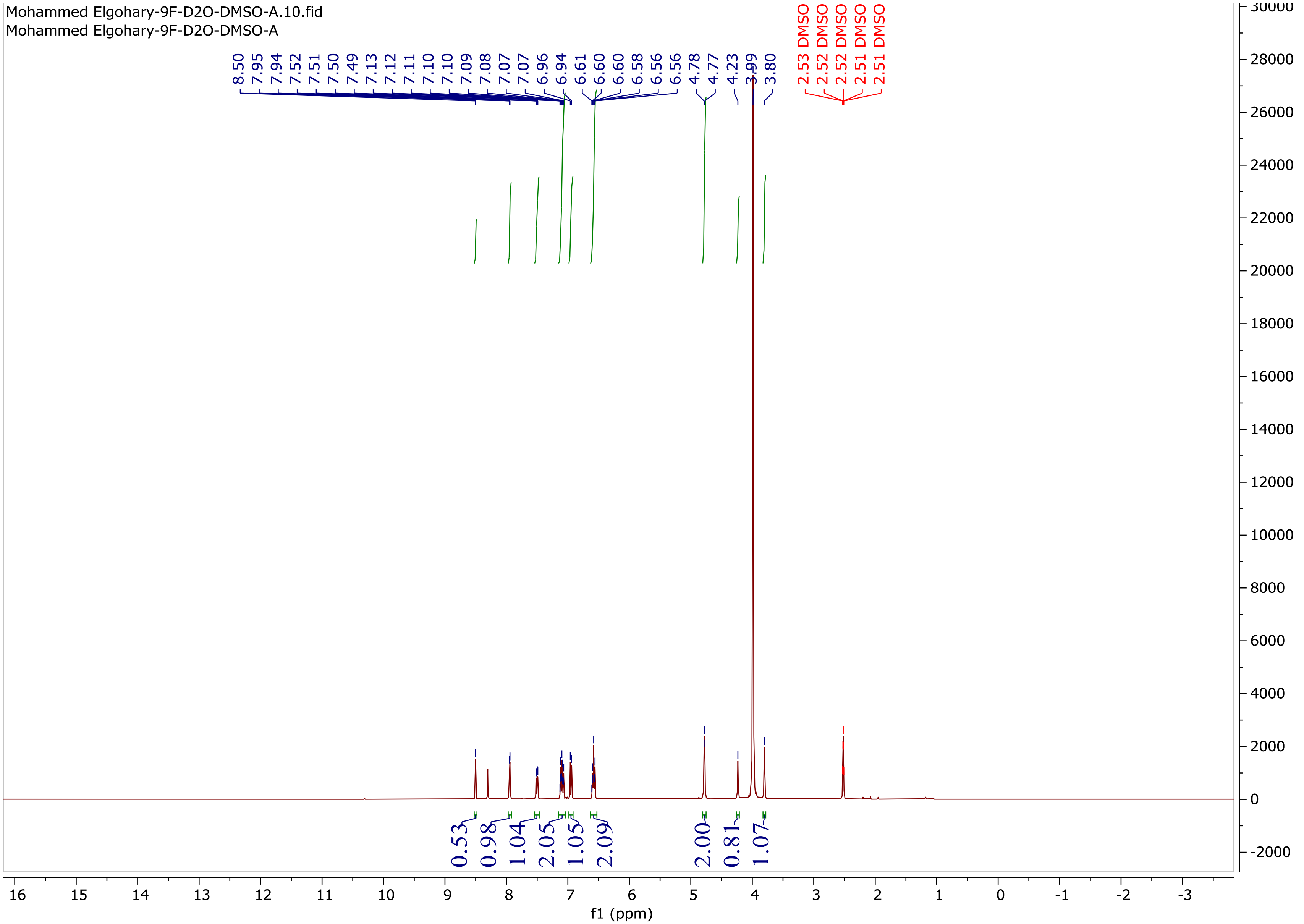

Figure S22. D_2O of compound **10f**

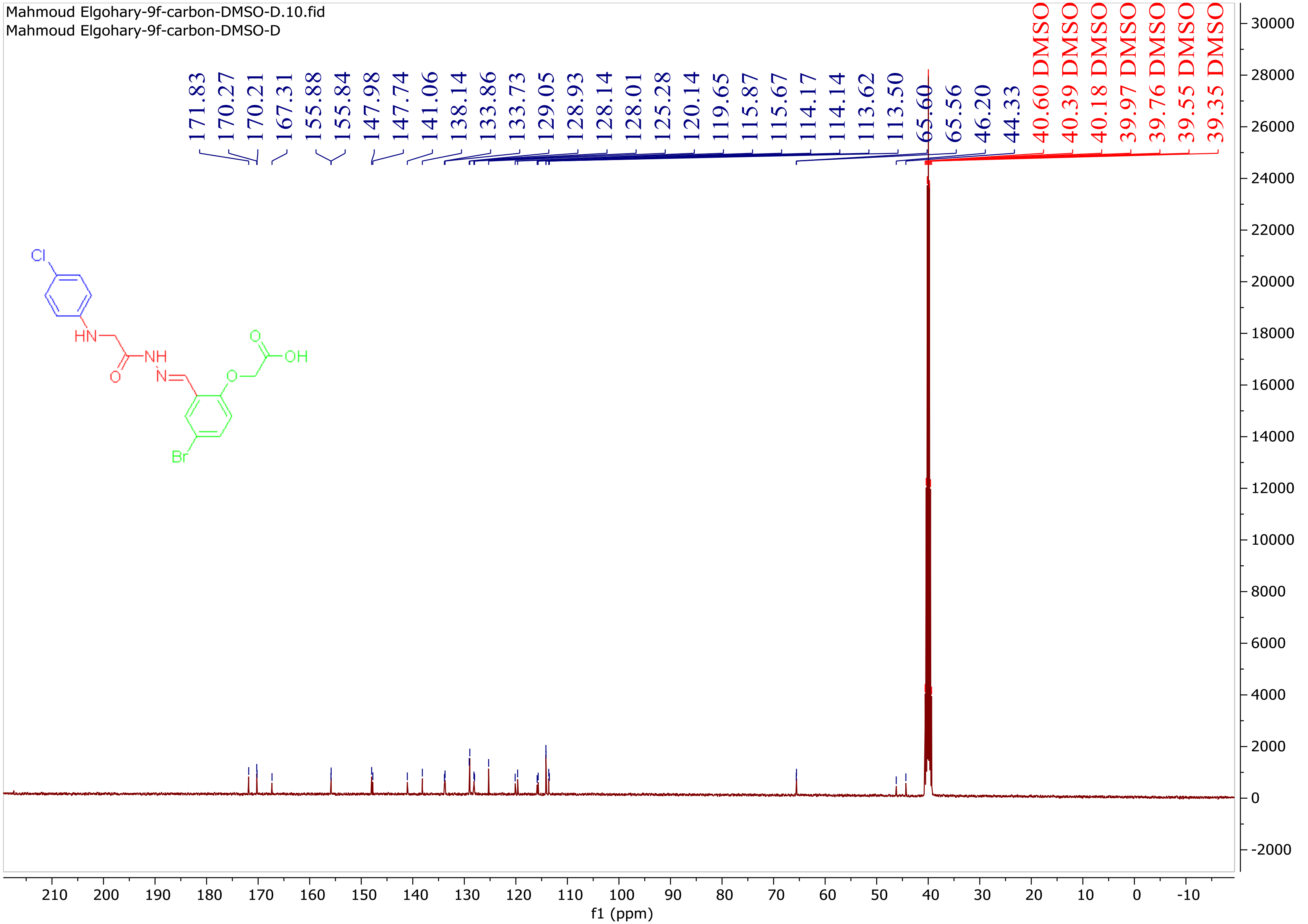

Figure S23. ^{13}C NMR of compound **10f**

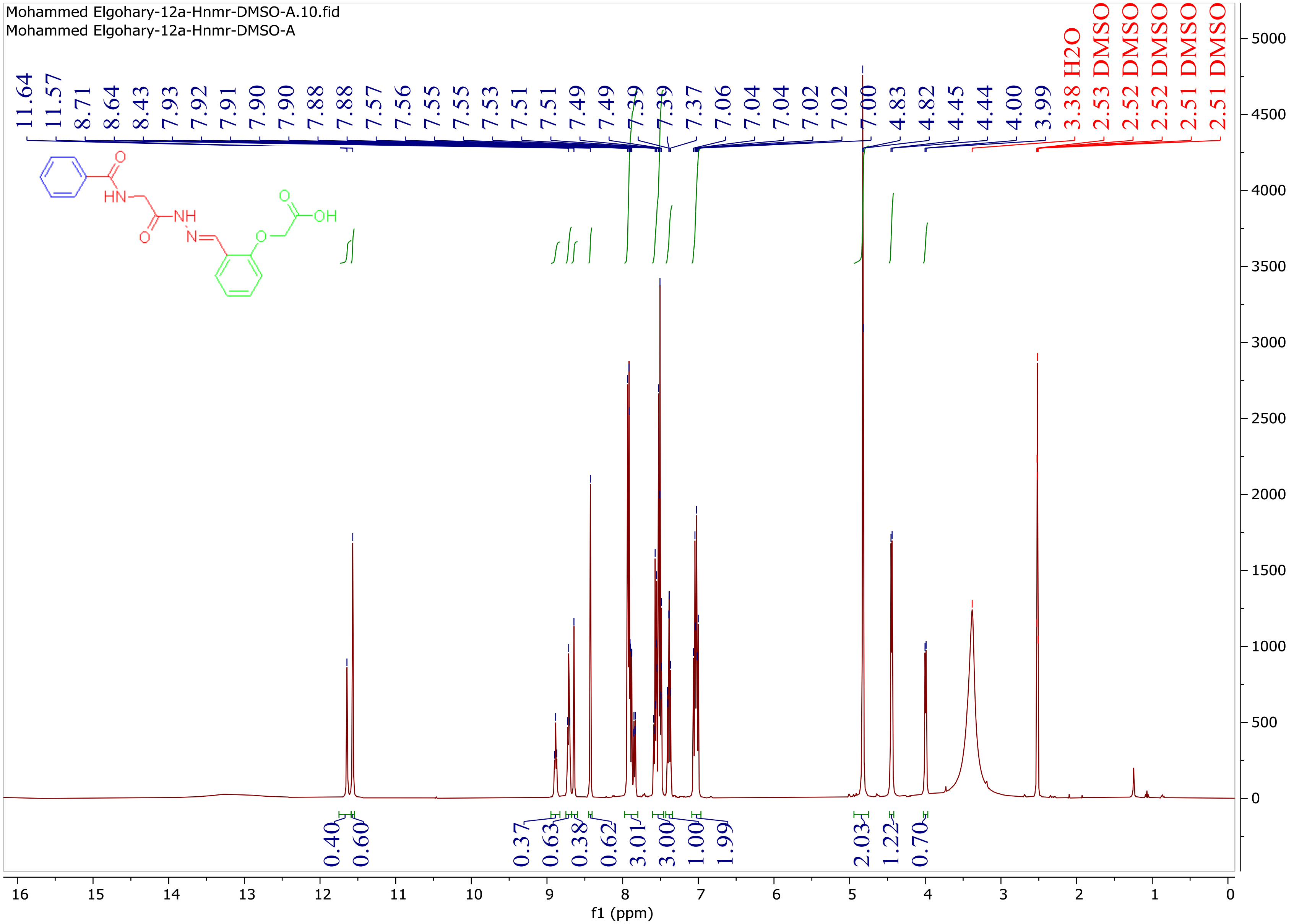

Figure S24. ^{1}H NMR of compound **13a**

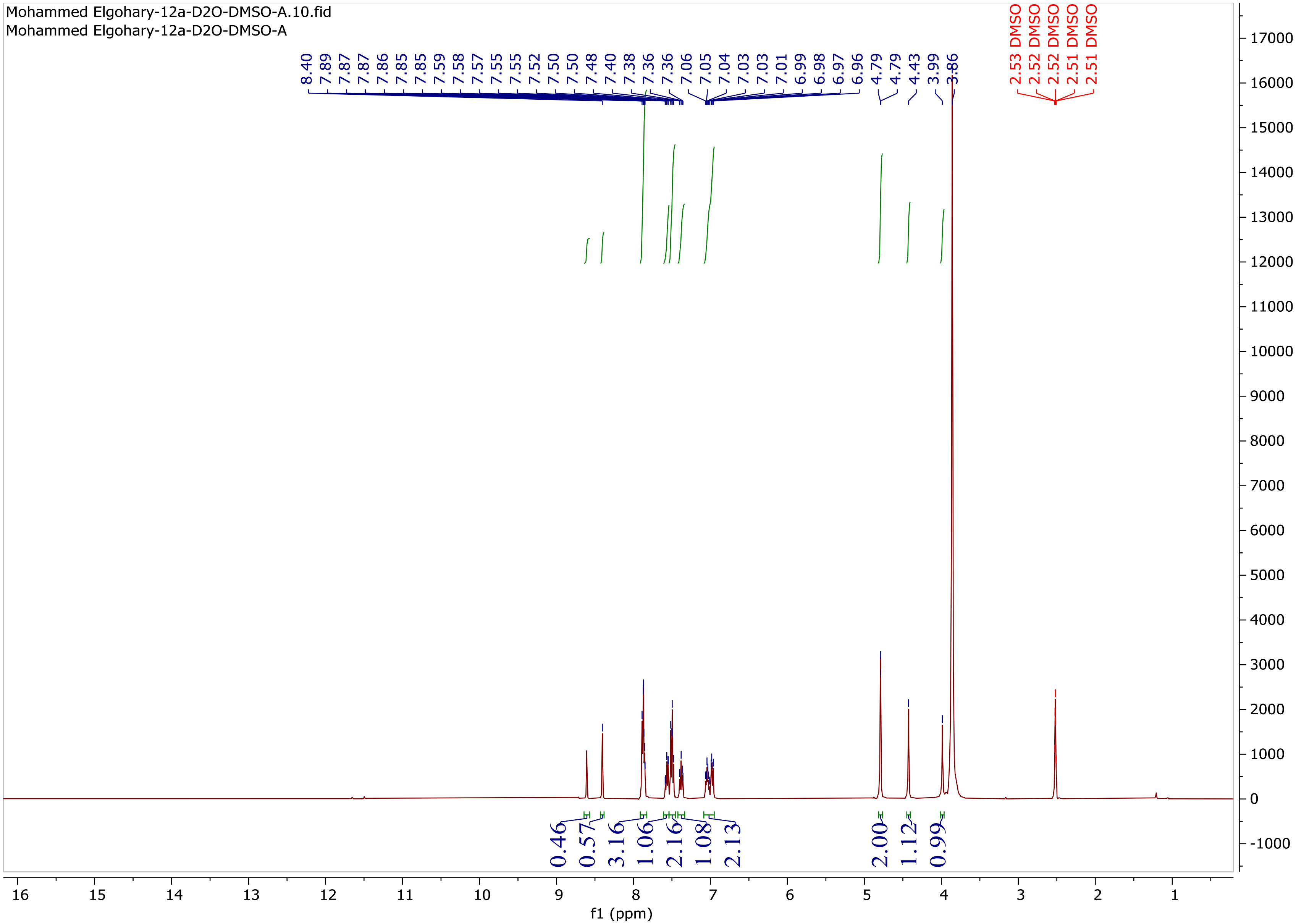

Figure S24. D_2O of compound **13a**

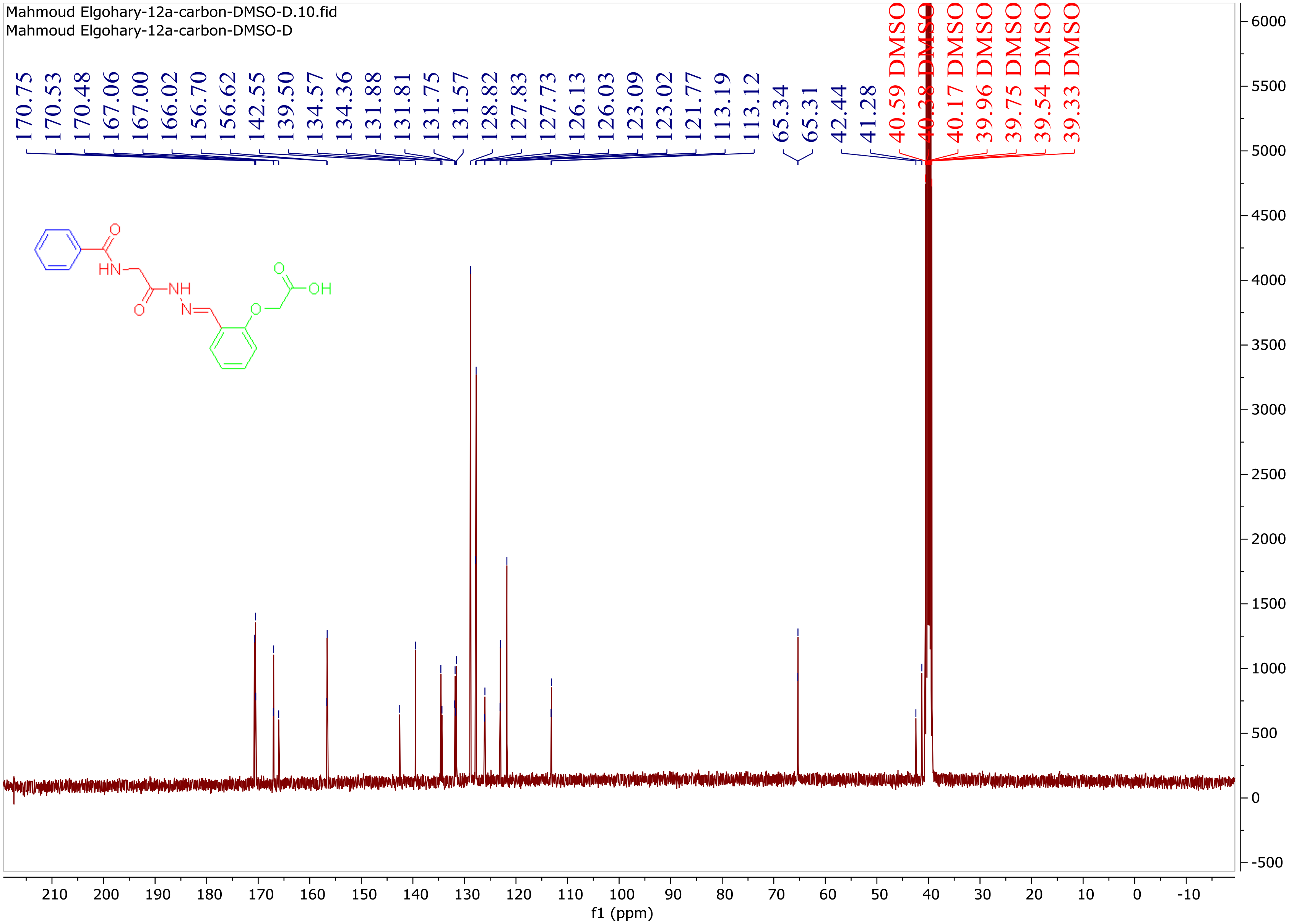

Figure S25. ^{13}C NMR of compound **13a**

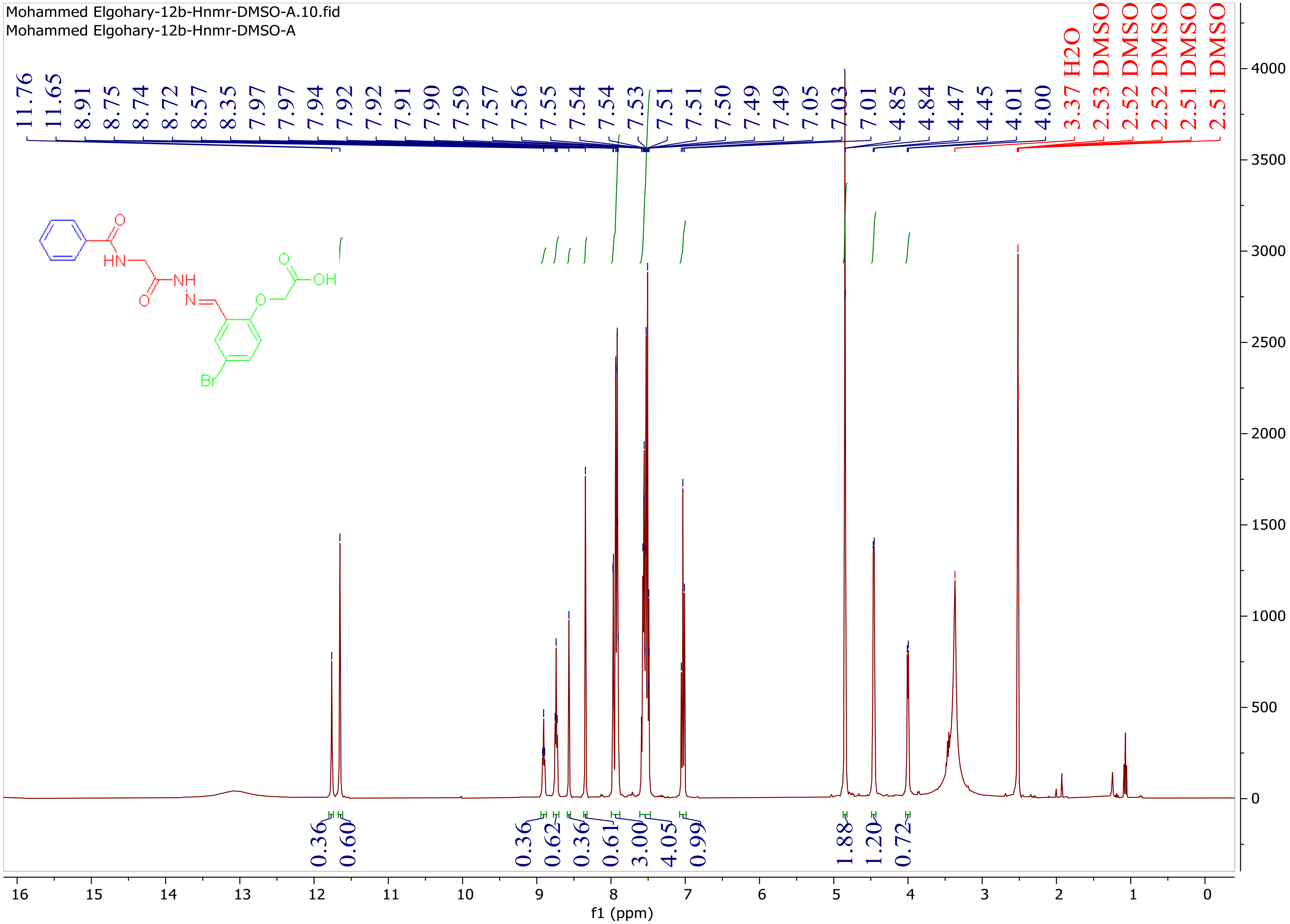

Figure S26. ^{1}H NMR of compound **13b**

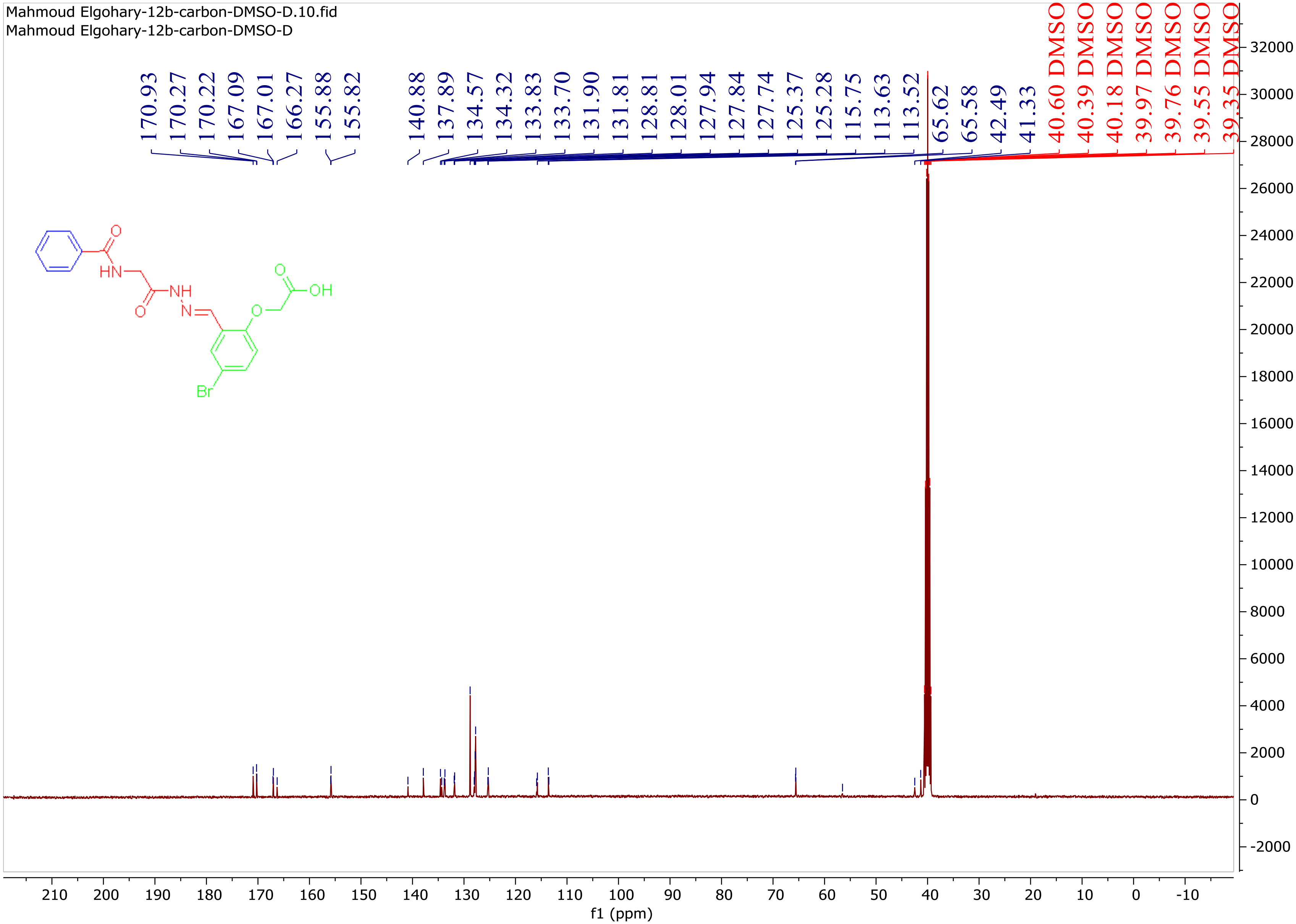

Figure S27. ^{13}C NMR of compound **13b**

Experimental procedures and instruments

Chemistry instruments

A Stuart digital apparatus was used for determining melting points and were uncorrected. Shimadzu FT-IR 8400S infrared spectrophotometer was used for recording infrared spectra. Both ^{1}HNMR and ^{13}CNMR were measured using deuterated di-methyl sulfoxide (DMSO) as solvent but with variable frequency (100MHZ for ^{13}CNMR, 400MHZ for ^{1}HNMR) *via* Bruker spectrophotometer, at the Faculty of Pharmacy, Ain-shams, Mansoura and Beni-suef Universities. Mass spectra were recorded by TLC-MS Advion compact mass spectrometer (CMS). Elemental analyses were implemented at the regional renter for microbiology and Biotechnology, Al-Azhar University, Cairo, Egypt.

In vitro cyclooxygenase (COX-1/COX-2) inhibition assay

The *in vitro* enzyme assays were performed at the Biochemistry Department, Cairo General Hospital, Egypt. Cayman® colorimetric COX (ovine) inhibitor screening assay kit (Item No. 560131 – Michigan, USA) was used for monitoring the activity of COX-1 and COX-2 enzymes in presence of all newly compounds. Generally, this kit is dependent on the reductive reaction of COX. A specific antibody binds to PGH_2 produced by COX and is further quantified by the ELISA technique as previously reported. After that, the selectivity index (SI) was calculated as the following equation:

$$\mathbf{Selectivity\ index\ (}\boldsymbol{SI}\mathbf{)} = \frac{\mathbf{IC_{50} of\ COX-1}}{\mathbf{IC_{50}\ of\ COX-2}}$$

Animals

Sprague Dawley rats, ranging in weight from 150 to 200 g, were obtained from the animal facility at the Egyptian Russian University Faculty of Pharmacy. The rats underwent a minimum of one week of acclimatization in the animal house prior to the initiation of the experiment.

Chemicals

Celecoxib and mefenamic acid were acquired in tablet form bearing the respective brand names. Before oral administration, the fragments were suspended in 1% carboxymethyl cellulose (CMC) after being ground in a porcelain mortar. Carrageenan was obtained from Sigma Aldrich Company (St. Louis, MO, USA) and diluted in 0.9% normal saline before paw edema induction.

In-vivo anti-inflammatory activity

The carrageenan paw edema test was conducted following established procedures. In summary, seventy-two rats were randomly assigned to twelve groups, each comprising six rats. These groups included normal controls, carrageenan controls (both receiving the vehicle; 1% CMC), reference drugs (celecoxib and mefenamic acid), and various test compounds (**5d-f, 7b, 10c-f**). Acute inflammation was induced in the right hind paws of all rats one hour after oral administration of the respective compounds or vehicle. This was achieved by injecting a freshly prepared 0.1 mL carrageenan suspension (1% in normal saline) subplantarly into the right hind paw, except for the normal control group, which received a saline injection. Paw thickness was measured hourly (Ct) until the fifth hour (C5) post-carrageenan injection (C0). The hourly increase in paw thickness (paw thickness difference) was calculated by subtracting the initial measurement (C0) from the thickness at each hour (Ct), and the results were presented as the mean increase in paw thickness (mm). To evaluate anti-inflammatory effects, the percentage of inhibition for each standard and test compound was determined using the formula: percentage of inhibition (%) = [(Paw thickness difference of carrageenan group - Paw thickness difference of treated)/ Paw thickness difference of carrageenan group] × 100. Following the experimental period, the animals were euthanized using pentobarbital sodium (100 mg/kg), and blood was collected via jugular vein exsanguination for later evaluation of liver and kidney function, and subsequently sacrificed by cervical dislocation. The paws were then excised and weighed (both left and right). The percentage of weight increase between the left and right paws of each animal was calculated using the equation: Percentage of weight increase = [(Weight of the right paw - Weight of the left paw) / Weight of the left paw] ×100. Following this, the animals' stomachs were removed, inflated, and opened along the greater curvature to evaluate ulcerogenic effects.

Assessment of inflammatory biomarkers using ELISA

After conducting the *in-vivo* anti-inflammatory test, the paws from various groups, including control, carrageenan, celecoxib, mefenamic acid, and selected test compounds (**5f** and **7b**), were reserved. These test compounds were chosen based on their favorable characteristics, including lower IC_{50}, the highest percentage of inhibition of paw thickness, and the lowest increase in paw weight. The exudates from the right paws were then evaluated for inflammatory biomarkers, specifically tumor necrosis factor-alpha (TNF-α) and prostaglandin E2 (PGE2)

content, using enzyme-linked immunosorbent assay (ELISA). The ELISA kits were purchased from CLOUD-CLONE CORP (CCC, USA) (Cat# SEA133Ra) and MyBioSource company (British Columbia, Canada) (Cat# MBS730592) respectively, and the assays were performed following the manufacturer's instructions. Additionally, the protein content of the exudates was determined using the Bradford method.

Analgesic activity

The analgesic efficacy of the most potent compounds identified in prior tests was determined using the hot plate latency test, following established protocols. In summary, thirty rats were randomly allocated into five groups: normal control, celecoxib, mefenamic acid, **5f** and **7b**. The normal control group received 1% CMC, while the remaining groups were administered their respective reference or test compounds. Each rat was gently positioned on a heated plate set at 50 ◦C. The latency period for nociceptive responses, such as paw licking or jumping off the hot plate, was recorded at 30-, 60-, 90-, and 120 minutes post-administration of the reference drugs, test compounds, or CMC. A 30 second cutoff latency was implemented to prevent heat-induced tissue damage.

Histopathological examination

Thirty-six rats were separated into six groups of six rats each for histological assessment of the most active compounds' effects on the carrageenan paw edema inflammatory model. The groupings consisted of a control group, a carrageenan group, two groups for the reference drugs celecoxib and mefenamic acid, and two groups for the test compounds **5f** and **7b**. The same protocols that were utilized in the *in-vivo* anti-inflammatory activity test were used. After a duration of 5 hours, the animals were euthanized, and their left paws were amputated. The paws were preserved in 10% neutral buffered formalin, trimmed, washed, dehydrated in increasing concentrations of ethyl alcohol, clarified in xylene, and ultimately embedded in paraffin. They were then sliced into 4-6µ thick slices, stained with Hematoxylin and Eosin, and examined under a light microscope. A scoring system was used to evaluate the degree of inflammation, dividing it into six categories ranging from 0 indicating no inflammation to 5 indicating severe inflammation. Each score corresponded to a certain level of inflammation: 0 for no inflammation, 1 for mild inflammation, 2 for mild to moderate inflammation, 3 for moderate inflammation, 4 for moderate to severe inflammation, and 5 for severe inflammation.

Toxicity assessment

Assessment of liver and kidney function

Toxicity assessment was performed on test compounds **5f** and **7b**, as well as the reference drugs celecoxib and mefenamic acid. Blood samples obtained from the animals used in the *in-vivo* anti-inflammatory activity test were centrifuged, and the resulting sera were collected for the assessment of liver and kidney function. Liver function was evaluated by measuring the alanine aminotransferase (ALT) and aspartate aminotransferase (AST) activities, while kidney function was assessed through the determination of serum creatinine and blood urea nitrogen (BUN) levels. These assessments were conducted using kits procured from Spectrum Diagnostics Company (Cairo, Egypt), following the manufacturer's instructions.

Evaluation of ulcerogenic effects

The assessment of ulcerogenic effects was conducted following established procedures. Examination under 3X magnification enabled the scoring of the number and severity of lesions in the glandular mucosa. The Lesion number ranged from 0 to 4, as previously mentioned **(A)**. While lesion severity scores ranged from 0 to 5, as previously described (**B)**. The samples were then fixed in 10% neutral buffered formalin, then trimmed, rinsed in water, dehydrated in escalating levels of ethyl alcohol, clarified in xylene, and finally embedded in paraffin. Thin sections measuring 4-6μ were subsequently made and stained with Hematoxylin and Eosin

A: Ulcer lesion number scoring system

**Score**	**Description**
0	No lesions
1	1-2 localized lesions
2	3-5 localized lesions
3	6-10 lesions
4	More than 10 lesions or diffuse (or very large) lesions

B: Ulcer lesion severity scoring system

Score	Description
0	No lesions
0.5	Diffuse hyperemia
1	1 to 2 small ulcers
1.5	3 to 6 small ulcers
2	7 to 10 small ulcers
2.5	More than 10 small ulcers
3	1 marked ulcer plus 0 to 4 small ulcers
3.5	1 marked ulcer plus 5 or more small ulcers
4	2 marked ulcers plus 0 to 4 small ulcers
4.5	2 marked ulcers plus 5 or more small ulcers
5	3 or more marked ulcers

Statistical analysis

The statistical analysis of the data was conducted using GraphPad Prism 9.5.1 Demo (GraphPad Software, San Diego, CA). One-way analysis of variance (ANOVA) was utilized to evaluate differences among the various groups, except for paw edema volume, which underwent analysis using two-way ANOVA. Post-hoc analysis was performed using Tukey's multiple comparisons test. The results are presented as mean ± standard deviation (SD), with statistical significance set at $P < 0.05$

Molecular Modeling study

Materials and methods

The 3D structure of human COX-2 receptor binding sites (PDB: 1CX2) was obtained from the Protein Data Bank (http://www.rcsb.org).

Docking Protocol

Docking studies with COX-2 candidate compounds were carried out using AutoDockVina 1.5.7. This software was run with a searching grid stretched over ligand molecules, box spacing of 20×20×20, and COX-2 x, y, z coordinates of 29, 25 and 13, with all other parameters set to default. The highest scoring site for each compound was chosen after docking into the crystal structures of COX-2. The ideal docking posture is believed to be the most stable conformation of each compound for binding to the protein-active site, the selection of the candidates was based on

docking energies. The Discovery Studio application v21.1.0.20298 was used to investigate the interactions between ligands and receptors based on the type and distance of interactions.

In silico **Drug-Likeness and ADMET Prediction**

The SWISSADME server, a free online program developed by the Swiss Institute of Bioinformatics, was used to compute physicochemical descriptors and anticipate ADME parameters, pharmacokinetic properties, druglike nature, and medicinal chemistry friendliness of our target compounds, as measuring pharmacokinetic properties and drug probability aspects is critical for the development of new compounds.
